# Resting cells rely on the DNA helicase component MCM2 to build cilia

**DOI:** 10.1093/nar/gky945

**Published:** 2018-10-17

**Authors:** Teresa Casar Tena, Lars D Maerz, Karol Szafranski, Marco Groth, Tamara J Blätte, Cornelia Donow, Sabrina Matysik, Paul Walther, Penelope A Jeggo, Martin D Burkhalter, Melanie Philipp

**Affiliations:** 1 Institute of Biochemistry and Molecular Biology, Ulm University, 89081 Ulm, Germany; 2 Leibniz Institute on Aging, Fritz Lipmann Institute, 07745 Jena, Germany; 3 Central Facility for Electron Microscopy, Ulm University, 89081 Ulm, Germany; 4 Genome Damage and Stability Centre, University of Sussex, Brighton BN1 9RQ, UK

## Abstract

Minichromosome maintenance (MCM) proteins facilitate replication by licensing origins and unwinding the DNA double strand. Interestingly, the number of MCM hexamers greatly exceeds the number of firing origins suggesting additional roles of MCMs. Here we show a hitherto unanticipated function of MCM2 in cilia formation in human cells and zebrafish that is uncoupled from replication. Zebrafish depleted of MCM2 develop ciliopathy-phenotypes including microcephaly and aberrant heart looping due to malformed cilia. In non-cycling human fibroblasts, loss of MCM2 promotes transcription of a subset of genes, which cause cilia shortening and centriole overduplication. Chromatin immunoprecipitation experiments show that MCM2 binds to transcription start sites of cilia inhibiting genes. We propose that such binding may block RNA polymerase II-mediated transcription. Depletion of a second MCM (MCM7), which functions in complex with MCM2 during its canonical functions, reveals an overlapping cilia-deficiency phenotype likely unconnected to replication, although MCM7 appears to regulate a distinct subset of genes and pathways. Our data suggests that MCM2 and 7 exert a role in ciliogenesis in post-mitotic tissues.

## INTRODUCTION

Cilia are small, membrane enclosed organelles, which emanate from the surface of postmitotic cells. They form at the onset of G0 phase of the cell cycle when the mother centriole of the centrosome attaches to the inside of the plasma membrane using distally positioned appendages. The molecular scaffold of cilia is built by microtubule doublets, which extend from the mother centriole into the extracellular matrix. The microtubule framework is ensheathed by a membrane that is distinct from the rest of the plasma membrane (1) and enriched for a plethora of signalling molecules (2). Functionally, cilia orchestrate a large part of the cell's signal transduction. Cilia sense chemical as well as mechanical signals and recruit receptors and second messengers to transmit the received signal into the cytoplasm. In addition, a subset of cilia is capable of propelling body fluids such as cerebrospinal fluid in the brain vesicles (3). During development, ciliary beating further produces the counter-clockwise flow of a yet undefined fluid in a temporal organizer of left-right asymmetry triggering the expression of genes exclusively on the left side of the body. As a consequence internal organs such as the heart, liver and spleen are arranged in their typical, asymmetrical fashion (4,5). Unfaithful ciliogenesis can cause a number of human disorders. These belong to the family of ciliopathies and include phenotypes such as nephronophthisis, microcephaly and situs defects. The latter are characterized by oftentimes very complex congenital malformations that arise from a failure to establish internal body asymmetry during development (2,4,6).

MCM proteins are a family of proteins that were identified as essential factors in minichromosome maintenance (MCM) in yeast (7). Six of these MCMs, namely MCM2–7 can be grouped further. They form a ring-shaped complex that is loaded onto DNA at the end of G1 and finishes origin licensing, which is initiated by origin recognition complex (ORC) proteins. At the onset of S phase, the DNA-loaded MCM complex becomes activated and serves as the unwinding helicase during replication (8,9). However, since there are many more MCM hexamers than firing origins it has long been suspected that MCM proteins including MCM2 have additional functions beyond promoting DNA synthesis (10). Consistent with this MCM paradox an unanticipated function of MCM4 had been reported, where MCM4 is causative for adrenal failure, potentially independently of its function during replication (11,12). For other MCMs and particularly MCM2, no additional function has been reported. Here we show a so far unanticipated function of MCM2 and MCM7 in cilium formation.

## MATERIALS AND METHODS

### Cloning and capped RNA synthesis

All PCRs for cloning of expression constructs were performed using Phusion polymerase (NEB). Full length zebrafish Mcm2 was amplified from 24 hours post fertilization (hpf) zebrafish cDNA and cloned by TOPO directional cloning (Invitrogen) into pcDNA3.1 with C-terminal His and V5 tags. To facilitate rescue constructs after antisense morpholino oligonucleotide (MO) injection the MO binding site was silently mutated. To generate the open reading frame (ORF) of zebrafish Mcm7, 5′-RACE PCRs (FirstChoice™ RLM-RACE Kit, Thermo Fisher) based on ENSDART00000159300.2 were performed on RNA from 13 somites stage (ss) embryos. The ORF (Genbank accession no. MH746781) was amplified from 24 hpf zebrafish cDNA and cloned by directional TOPO cloning as described for Mcm2. The ORF of human MCM7 was then amplified from cDNA of human fibroblasts (described in cell culture section). Capped RNA was transcribed from these plasmids after linearization using PmeI and using the T7 mMessage mMachine Kit (Ambion).

### Zebrafish husbandry and manipulation

Zebrafish were maintained in a circulating water system and a 14 h light and 10 h dark cycle. Eggs were generated by natural matings and allowed to develop until the desired stage in an incubator set to 28.5°C. Microinjections into the yolk were performed at the one cell stage for ubiquitous administration or at the 1000 cells stage to target dorsal forerunner cells (13). Antisense MOs were designed and synthesized by Gene Tools Inc (Oregon USA) based on submitted sequences or previous publications. MOs used were Mcm2 MO: 5′- CGACTCTGAGGAATCCGCCATTTTC, Mcm2 CTRL: 5′-CCACTCTCAGGCATCCTCCATTATC, Mcm2 splMO: 5′-GAAAAGTGCATCTCTCTCACCTCTC, Mcm2 splCTRL: 5′- GAATACTGGATCTCTGTGACCTCTC, Mcm7 MO: 5′-CCCGGAGTCATCCTCAGTGTAGAAC, and the standard control MO: 5′- CCTCTTACCTCAGTTACAATTTATA. Drug treatments of fertilized eggs were from the tailbud stage on with the concentrations as indicated. 1% DMSO in egg water served as vehicle control. The Bloom helicase inhibitor ML216 was purchased from Sigma-Aldrich (Germany) and dissolved in DMSO. Husbandry of and experiments with zebrafish were approved by the local animal welfare authority in accordance with Ulm University.

### MO efficiency analysis

The efficiency of the Mcm2 MO targeting the start codon of Mcm2 was assessed in an in vitro translation assay as described in Burkhalter *et al.* (14). Splice blocking of the second Mcm2 MO was tested by RT-PCR with primers flanking the exon intron boundary at the 3′ end of exon 2. The sequences of the primers were: Mcm2 splFw: 5′-ATG GCG GAT TCC TCA GAG T, Mcm2 splRev: 5′-GTT CTC TGT CGC GTC TCC TC, Gapdh Fw: 5′-ACA TTA AGT GGG GTG ATG CAG, Gapdh Rev: 5′-CCA TCA ACG GTC TTC TGT GTT. The resulting higher band was extracted, cloned into pCRII by TOPO TA cloning (Life technologies, Germany) and sequenced for verification of intron retention and the generation of a premature stop codon. We similarly performed RT-PCRs for the Mcm7 MO using the following primers: Mcm7 Fwd: 5′-CAC CAT GGC CCC GAA GGA TTA TAC, Mcm7 Rev: 5′-GAC CTC TGA CCT CCA TCA TCA. This also resulted in a higher band, which was cloned into pCRII by TOPO TA cloning and sequence verified as a product of splicing blockade resulting in a premature stop codon.

### *In situ* hybridization

Whole mount *in situ* hybridization was carried out as described in the standard protocol by Thisse *et al.* (15). Probes to detect mRNAs of interest were in vitro transcribed from linearized plasmids using SP6 or T7 polymerases (NEB, Germany) and the DIG RNA labelling system (Roche, Germany). The *mcm2* probe was designed based on the Genbank sequence NM_173257. A probe covering the whole open reading frame was used to detect *mcm7*. All other probes have been described before (14,16).

### Cell culture and transfection

hTert immortalized 1BR3 fibroblasts were from the collection of the Genome Damage and Stability Centre at the University of Sussex and have been tested negative for mycoplasma. hTert fibroblasts were cultured in MEM containing 10% heat-inactivated fetal calf serum (FCS), 1% Penicillin/Streptomycin (all Life technologies, Germany) and 1% non-essential amino acids (Sigma-Aldrich, Germany) at 37°C in a humidified atmosphere containing 5% CO_2_. HEK293T cells were maintained in DMEM with 10% FCS, 1% Penicillin and 0.75% sodium bicarbonate (all Life technologies, Germany). Transfection with SMART Pool siRNAs (Dharmacon, Germany) was achieved using Lipofectamine RNAiMax reagent (Life technologies, Germany). In brief, cells were transfected with the siRNA-Lipofectamine complex during seeding. 48 hours post transfection cells were serum starved in culture medium containing 0.1% FCS for another 48 h to induce cilia formation (with optional α-amanitin treatment (0.1 μg/ml; Sigma)). Subsequently, cells were fixed in either 4% PBS-buffered paraformaldehyde or ice-cold methanol. For MCM2 knockdown in non-replicating cells, cells were seeded at a density of 150,000 cells per well in 6-well plates and allowed to grow for one day before medium was changed to starvation medium. Transfection was carried out 24 h post onset of serum starvation. Cilia were analysed 2 days after transfection in G0, while qPCR analysis was performed 4 days post transfection. During the whole time, cells were serum starved to maintain quiescence. For rescue experiments nucleoporation of siRNAs along with plasmids encoding either zebrafish Mcm2 or 7 were performed using the Amaxa nucleofector II, the Amaxa Cell Line Nucleofector Kit R (both Lonza) and program U-023. To ensure that all nucleofection conditions contained the same amount of plasmid DNA, siCTRL and siMCM2/7 were substituted with empty vector. Two days after nucleofection cells were changed to serum starvation and processed as described after Lipofectamine transfection.

### qPCR

For qPCR analysis total RNA was isolated from transfected cells using Qiagens’ RNeasy mini spin columns (Qiagen, Germany) or using Zymo's Quick-RNA MiniPrep columns, both with an additional DNaseI digest. Equal amounts of RNA were reversely transcribed into cDNA using oligodTTPs and Superscript II or III reverse transcriptase (Life technologies, Germany), respectively. qPCR was performed with Absolute QPCR ROX Master Mix (Thermo Fisher, Germany) or Luna Universal probe qPCR Master Mix (NEB) and the Roche Universal Probe System on a Roche LightCycler 480 using primers spanning introns whenever possible. Supplementary Table S1 lists all primers and probes used. Further information on qPCR is also given in the MIQE protocol in the supplement.

### Western blotting

Transfected HEK cells as well as asynchronous and serum starved 1BR3 cells were lysed in SDS lysis buffer (2% SDS and 50 mM Tris pH 6.8) containing protease and phosphatase inhibitors (Roche, Germany). Lysates were cleared by nuclease treatment (Thermo Fisher) and centrifugation. Equal protein amounts were separated on NuPage 4–12% Bis–Tris gels (Life technologies, Germany) and blotted onto nitrocellulose membranes (Bio-Rad Laboratories, Germany). After blocking with 5% skim milk powder in Tris buffered saline containing 0.2% Nonidet P-40 (Sigma Aldrich, Germany), blots were incubated with primary antibody over night at 4°C. Secondary antibodies coupled to near infrared dyes (LICOR Biosciences, Germany) were used for signal detection with a LI-COR Odyssey SA (Germany). Antibodies used for Western blotting were: mouse anti-beta actin (1:10 000, catalog no. A1978, Sigma, Germany), rabbit anti-Cyclin A (1:1000, catalog no. sc-751, SCBT, Germany), rabbit anti-Mcm2 (1:1000, clone D7G11, Cell Signaling, Germany), rabbit anti-PCNA (1:200, catalog no. sc-7907, SCBT, Germany) and mouse anti-Gapdh (1:500, clone 6C5, Acris antibodies, Germany). For cell cycle analysis bands were quantified using the Li-COR Odyssey SA software version 1.1. Cropped images of western blots are shown as representative images.

### Flow cytometry

Cells were loaded with 50 μM BrdU for 1 h at 37°C. Afterwards cells were trypsinized, pelleted by centrifugation and resuspended in PBS. Fixation was done by addition of absolute ethanol while vortexing and incubation at −20°C overnight. Cells were pelleted again, resuspended in 0.5%Tween/4N HCl in PBS and incubated at room temperature for 30 min. After addition of 1 M Tris pH 8 cells were centrifuged and resuspended in PBS containing 0.5% Tween-20 and an AlexaFluor 647 anti-BrdU antibody (catalog no. 364108, BioLegend, Germany, 1:10). RNase A treatment and PI co-staining was done using a commercially available Propidium Iodide (PI)/RNase Staining Solution (Cell Signaling, Germany). Cell cycle profiles were acquired with a FACSCalibur flow cytometer and with CellQuest Software (both BD Biosciences, Germany).

### Immunocytochemistry

Zebrafish embryos were stained as described in Burkhalter *et al.* (14). Antibody staining in fibroblasts was done with cells grown on coverslips. In brief, after fixation with either 4% PFA or ice-cold methanol, cells were permeabilized with 1% Triton X-100 and subsequently blocked with 10% normal goat serum (Vectorlabs, UK) in PBS. Primary antibodies diluted in blocking buffer were incubated over night at 4°C. After three washes in PBS, cells were incubated with secondary antibody diluted in blocking buffer. After three more washes coverslips were mounted onto slides with Vectashield containing Dapi (Vectorlabs, UK). Primary antibodies were mouse anti-acetylated tubulin (1:500, clone 6-11B-1, Sigma-Aldrich, Germany), rabbit anti-PKCζ (1:500, catalog no. sc-216, SCBT, Germany), mouse-anti-γTubulin (1:5000, clone GTU-88, Sigma-Aldrich, Germany), rabbit anti-γTubulin (1:500, cat. no. T5192, Sigma-Aldrich, Germany), rabbit anti-Cenexin 1 (1:100, kind gift of Kyung Lee, CCR), rabbit anti-centrin1 (1:300, catalog no. 12794-1-AP Proteintech, Germany), rabbit anti-pericentrin (1:500, cat. no. HPA019887, Sigma-Aldrich, Germany). Alexa-coupled secondary antibodies (Molecular Probes, Germany) were used at a 1:1000 dilution.

### Cryo-electron microscopy

hTert fibroblast were seeded on sapphire discs and simultaneously transfected with CTRL and MCM2 siRNAs (as described before). Cells were grown to confluence and serum starved for another 48 h. Sapphire discs containing transfected cells were high-pressure frozen without previous chemical fixation using the Wohlwend HPF Compact 01 (Engineering Office M. Wohlwend GmbH, Switzerland) as described in (17). Freeze-substitution was facilitated by immersion in acetone pre-chilled to −90°C containing 0.2% osmium tetroxide, 0.1% uranyl acetate and 5% to enhance membrane contrasting (all reagents: Merck, Germany) in a computer-assisted substitution apparatus. In there the samples were brought to 0°C over a time of 16 h. Thereafter, the samples were acclimatized to room temperature and Epon (Fluka, Germany) embedded. 60 nm sections were cut using a Leica Ultracut UCT ultra-microtome with a diamond knife (Diatome, Switzerland) and collected on copper grids. Finally, sections were post-stained using lead citrate. Imaging of ultra-thin sections was performed on a JEM-1400 transmission electron microscope (Jeol GmbH, Germany) at an acceleration voltage of 120 kV. To record the images a Veleta digital camera (Olympus Soft Imaging Solutions GmbH, Germany) with a resolution of 2024 Å–2024 pixel and the iTEM software (Olympus Soft Imaging Soluions GmbH, Münster, Germany) were used.

### RNA sequencing and bioinformatical analysis

Total RNA from control, siMCM2 and siMCM7 transfected fibroblasts was isolated after cilia induction through 3 days of serum starvation. Quality and quantity of total RNA was checked using the Agilent Bioanalyzer 2100 and the RNA 6000 Nano Kit (Agilent Technologies, Germany). RNA integrity numbers (RIN) varied between 9.7 and 10. Library preparation and sequencing was done using Illumina technology (Illumina) (18). In detail, library preparation was done using TruSeq stranded mRNA sample preparation kit following the manufacturer's description (preparation of the libraries included a purification of polyA+ molecules and each library was indexed by an individual sequence.) The libraries were quality checked and quantified using the Agilent Bioanalyzer 2100 and the Agilent DNA 7500 kit. Sequencing was done using a HiSeq2500 in high-output, 50nt single-end read mode. All samples were sequenced in one lane, which resulted in 25–196 mio reads per sample. Sequence information was extracted in FastQ format using the software bcl2fastq v1.8.4 provided by Illumina. Mapping of reads to the reference was done using TopHat v2.1.0 (19) using parameters -T (map reads to the annotated loci only) and -x 1 (allow only unique mappable reads). As reference the human Ensemble genome version GRCh38 was used taking the gene annotation GRCh38.83 into account. Depending on the sample 87–88% of the reads could be mapped uniquely to the reference sequence. Based on the mapping the reads were assigned to the respective gene annotation with the help of featureCounts (20) using parameter -s 2 for counting strand-specific reads and the annotation as described above. Gene counts were further processed using the programming language R. In order to find differentially expressed genes (DEG) the statistical packages edgeR (21) and DESeq (22) were applied. P-values were adjusted for multiple testing using the Benjamini-Hochberg correction algorithm. Genes were regarded to be differentially expressed when corrected p-values of both tests were <0.05. Finally, DEGs with a fold change of at least 1.5 were classified into pathways using Ingenuity Pathway analysis (Qiagen, CA, USA).

### RNAseq consensus sequence analysis

Gene promoter sequences were extracted from the human genome release GRCh38, using the Ensembl gene annotation v26 and defining the promoter area as 3000 bp upstream plus 1000 bp downstream of the transcription start site. CpG islands were modelled using newcpgreport from the EMBOSS package (-window 400 -shift 1 -minlen 500 -minoe 0.65 -minpc 55) (23). Enrichment analysis was done using a hypergeometric test. Enrichment of sequence motifs was tested using the MEME suite (DREME) (24,25) with default settings.

### ATAC-seq and bioinformatical analysis

Libraries for ATAC-seq were prepared as described by the Greenleaf lab (26). Human fibroblasts were transfected with siRNAs (either targeting MCM2 or a control siRNA) and starved during 3 days as described above (*n* = 3 each). Cells were harvested, counted and aliquots of 50’000 cells washed once with cold PBS. Cell pellets were then resuspended in lysis buffer (10 mM Tris–HCl, pH 7.4; 10 mM NaCl; 3 mM MgCl_2_; 0.1% IGEPAL CA-630), immediately pelleted and further processed with the Nextera Kit (Illumina). To this end, cell pellets were resuspended in 25 μl buffer TD, 2.5 μl TDE1 and 22.5 μl H_2_O, incubated during 30 minutes at 37°C, following purification with the MinElute PCR Purification kit (Qiagen). Transposed DNA fragments were amplified during 5 cycles using the NEBNext High-Fidelity 2× PCR Mix (New England Biolabs) with the forward primer Ad1-noMX and a barcoded reverse primer (see Supplementary Table S1 for sequences). Amplification rates were assessed in a side qPCR using 10% of the previous PCR reaction as template, the previously used primer pair, the iTaq Universal SYBER Green Supermix (Bio Rad) and a Light Cycler LC480 II (Roche). The number of additionally needed amplification cycles was estimated by determining the cycle number at which 30% of the maximal relative fluorescence was reached. The remaining 90% of the original PCR reaction volume was then further amplified with the individually determined cycle number. The amplified products were first purified with the MinElute PCR Purification kit (Qiagen) and small fragments removed using Agencourt AMPure beads (Beckman Coulter). Fragment sizes were then assessed with the Bioanalyzer High-Sensitivity DNA Analysis kit on a Bioanalyzer 2100 (both Agilent).

Sequencing was carried out on a HiSeq2500 in rapid, 50nt paired-end mode. Libraries were pooled and sequenced on two lanes, generating on average 41 million read-pairs per sample. Sequence information was extracted in FastQ format using the software bcl2fastq v1.8.4. Mapping of reads to the reference was done using Bowtie2 (version 2.2.9) (27) using parameters –no-mixed (suppress unpaired alignments for the paired reads) and –fr -X 1000 (forward-reverse read topology with maximum of 1000nt insert size). As reference the human Ensemble genome version GRCh38 was used, later taking the GENCODE gene annotation GRCh38.v26 into account. Using samtools (28), it was verified that mappings contained only consistently paired reads (flag selector -F 0 × 904), and output was filtered for reads mapping outside nuclear chromosomal regions. Differential ATAC peaks were then called using MACS2 (version 2.1.1.20160309 (29), with the data combined per group. Differential peak calling was repeated with the groups swapped in order to find signal changes in both directions, and results were combined. Peak regions were filtered using a blacklist from the ENCODE project (www.encodeproject.org, Accession ENCSR636HFF), and we also excluded regions which formed characteristic clusters (1–10 kb), frequently overlapped blacklist regions and were predominantly gene-free (49% of the called peaks). The resulting candidate list contained 292 peaks with clear chromosomal mapping location, of which 227 (78%) were associated with a gene locus in ≤5 kb distance.

### Chromatin Immunoprecipitation (ChIP)

ChIPs were essentially performed as previously described (30). 1.5 × 10^5^ cells were seeded in 6-well plates in standard medium (see above) and cultured during 24 h. Cells were then kept during 3 days under starving conditions (0.1% FCS) to induce synchronization in G0. Medium was removed and cells were crosslinked during 9 min at room temperature with 1% formaldehyde (Thermo Scientific) in 1× PBS containing 2% FCS. Quenching of formaldehyde was achieved by addition of glycine to a final concentration of 125 mM. Cells were harvested, washed twice with 1x PBS containing 2% FCS and stored at −80°C. Cell pellets were resuspended in sonication buffer (0.1% SDS, 1% Triton X-100, 0.1% Na-deoxycholate, 1 mM EDTA, 140 mM NaCl, 50 mM HEPES pH 7.9, protease inhibitors (Complete Mini EDTA-free (Roche)) and incubated during 10 minutes on ice. Cells were then sonicated to obtain fragments of average length of 500 bp using a Bioruptor (Diagenode) with the setting ‘high’, 3 × 10 cycles of 30 s on and 30 s off. Sheared chromatin was cleared by centrifugation and pre-adsorbed with 5 μl of Protein G Dynabeads (Invitrogen) during 1 h at 4°C under rotation. Beads were pelleted on a DynaMag-2 stand (Invitrogen), 2 × 5 μl of the supernatant removed as input control and fragmentation control, respectively, and the rest transferred to a fresh tube and incubated under rotation at 4°C overnight with antibody (2.5 μl anti-MCM2 (Cell Signaling, catalog no. 3619, Germany), 2.5 µl anti-MCM7 (Cell Signaling, catalog no. 3735) or 3 μl rabbit IgG Santa Cruz (catalog no. sc-2027, Germany)). Protein G Dynabeads (20 μl per reaction) were washed twice with sonication buffer, added to the chromatin samples and incubated during 2–3 h at 4°C under rotation. Beads were subsequently washed twice with sonication buffer, twice with NaCl Buffer (0.1% SDS, 1% Triton X-100, 0.1% Na-deoxycholate, 1 mM EDTA, 500 mM NaCl, 50 mM HEPES pH 7.9), twice with LiCl buffer (350 mM LiCl, 1% IGEPAL CA-630, 1% Na-deoxycholate, 1 mM EDTA, 10 mM Tris–HCl pH 8.0) and twice with TE buffer (1 mM EDTA, 10 mM Tris–HCl pH 8.0). Beads as well as input controls were resuspended in 100 μl elution buffer (1% SDS, 100 mM NaHCO_3_, 250 mM NaCl, 0.5 mg/ml Proteinase K) and incubated during 45 min at 45°C followed by incubation during 3–4 h at 65°C. DNA was purified with the MinElute PCR Purification Kit (Qiagen, Germany). Eluted DNA was used as template to assess enrichment levels of specific genomic regions by qPCR using the Universal Probe system (Roche). Promoter regions of following genes were analysed: AURKA, UBE2C and CDC25C and compared to an upstream region of the gene. Assays are listed in Supplementary Table S1. qPCR reactions were carried out with Absolute qPCR ROX Mix (Thermo Scientific, Germany) in a Lightcycler 480 II (Roche, Germany). Recruitment of MCM2 and MCM7 to transcription start sites of AURKA, UBE2C and CDC25C was assessed by first normalizing results to the respective input controls, followed by normalization to a control region upstream of each gene.

### Imaging

Zebrafish live images and those of whole mount *in situ* hybridizations were acquired using a Leica M125 upright microscope equipped with a Leica IC80 HD or a Leica MC190 HD camera. Confocal images of the ciliated KV, the surrounding tissue and the pronehphros were acquired with a Leica TCS SP5II confocal microscope and a 40x oil immersion objective. Cilia length as well as KV area were measured using ImageJ (31). Cilia counts in cultured cells were obtained with a Zeiss Axiophot and a Hamamatsu ORCA-03G camera.

### Statistical analysis

All statistical analysis was performed with GraphPad Prism 7. Graphs display, if not indicated otherwise, means ± SEM. Data were tested for normality and analysed accordingly by parametric or non-parametric tests.

## RESULTS

### Zebrafish embryos depleted of Mcm2 display ciliopathy-phenotypes

**Figure 1.**
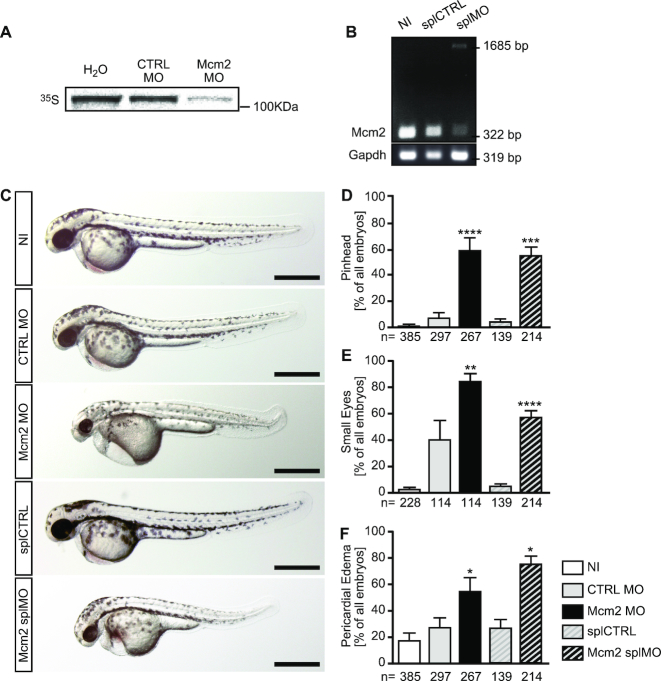
Morphological analysis of zebrafish lacking Mcm2. (**A**) Autoradiogram to show the binding efficiency of the Mcm2 MO. *In vitro* translation of Mcm2 was done in the presence of CTRL (CTRL MO) and translation blocking MO (Mcm2 MO) using a cell-free reticulocyte lysate and 35S-labeled methionine. As template, pCS2+ containing parts of the 5′-UTR fused to the ORF of Mcm2 was used. *n* = 3 MO binding tests (in triplicate). (**B**) RT-PCR of non-injected (NI), control injected (splCTRL) and splice blocking injected (splMO) embryos at 24 hpf. In embryos with Mcm2 splMO the original band at 322 bp partially disappeared. Instead a second band at 1685 bp could be detected, which contained parts of intron 2. *n* = 2 independent experiments. (**C**) Live images of zebrafish at 48 hpf. Scale bars: 500 μm. (**D**) Mcm2 depleted embryos develop smaller anterior structures. Numbers of embryos analysed are given below the bars. 3–10 independent experiments. **** indicates a *P* value ≤0.0001, *** means *P* ≤ 0.001. One-way ANOVA with Sidak's multiple comparison test. (**E**) Loss of Mcm2 impairs eye development. Numbers of embryos analysed are given below the bars. 3–6 independent experiments. **** indicates a *P* value ≤0.0001, ** means *P* ≤ 0.01. One-way ANOVA with Sidak's multiple comparison test. (**F**) Zebrafish lacking Mcm2 show a tendency to develop pericardial edema. Numbers of embryos analysed are given below the bars. 3–11 independent experiments. * indicates a *P* value <0.05. One-way ANOVA with Sidak's multiple comparison test.

**Figure 2.**
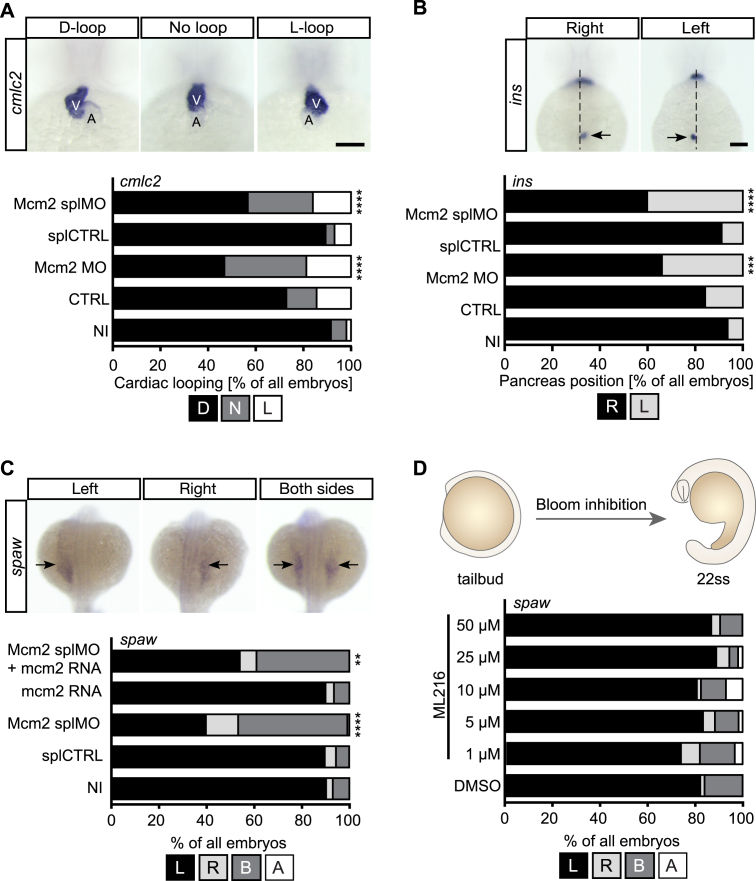
Loss of Mcm2 disrupts left-right asymmetry development. (**A**) Heart looping is randomized upon Mcm2 knockdown. Upper panel shows representative images of correct (D-loop) and aberrant heart looping (no loop, L-loop) at 48 hpf after *in situ* hybridization (ISH) for *cmlc2*. A, atrium; V, ventricle. Scale bar: 100 μm. Stacked bar graph summarizes heart looping experiments. D, D-loop; N, no loop; L, L-loop. *P* = 0.0003 (CTRL vs Mcm2 MO), *P* < 0.0001 (splCTRL vs Mcm2 splMO). *n* = 5–10 experiments. Number of embryos: NI = 309; CTRL = 166; Mcm2 MO = 128; splCTRL = 113; Mcm2 splMO = 194. (**B**) Example images of *insulin* (*ins*) ISH labelling of the endocrine pancreas (arrows). R, right (correct) or L, left (wrong position) of the embryonic midline. Scale bar: 100 μm.Graph displays quantification of aberrant pancreas position upon Mcm2 knockdown. *P* = 0.0052 (CTRL vs Mcm2 MO), *P* < 0.0001 (splCTRL vs Mcm2 splMO). *n* = 4–9 experiments. Number of embryos: NI = 299; CTRL = 146; Mcm2 MO = 109; splCTRL = 113; Mcm2 splMO = 192. (**C**) Loss of Mcm2 results in ambiguous *southpaw* (*spaw*) expression at 22 ss, which can be partially rescued by co-injection of *mcm2* RNA. Upper panel shows examples of correct and aberrant *spaw* distribution. *P* < 0.0001 (splCTRL vs Mcm2 splMO), *P* = 0.0029 (splMO vs Mcm2 splMO + *mcm2* RNA). *n* = 3 experiments. Number of embryos: NI = 156; splCTRL = 166; Mcm2 splMO = 152, *mcm2* RNA = 142; Mcm2 splMO + *mcm2* RNA = 181. (**D**) ML216-mediated inhibition of Bloom and Werner helicases from tailbud stage until 22ss does not affect asymmetry development. *n* = 3 experiments. Number of embryos: DMSO = 56; 1 μM ML216 = 61; 5 μM ML216 = 60; 10 μM ML216 = 57; 25 μM ML216 = 54; 50 μM ML216 = 53. All data analysed using two-tailed Fisher's exact test.

To examine extended functions of MCM proteins *in vivo*, we performed loss-of-function experiments in zebrafish using antisense morpholino oligonucleotides (MO) mediated knockdown against Mcm2. We designed two MOs blocking either translation (Mcm2 MO) or splicing (Mcm2 splMO). Since none of the antibodies tested worked in Western blots on zebrafish lysates, we tested the efficacy of both MOs using an in vitro translation assay for Mcm2 MO (Figure 1A) and RT-PCR to show aberrant splicing (Figure 1B), respectively. This revealed that injection of the Mcm2 splMO resulted in retention of intronic sequences and the insertion of a premature stop codon. Importantly, zebrafish depleted of Mcm2 with either MO developed pinheads and smaller eyes (Figure 1C–E), which mimicked the previously published Mcm2 mutant (32). This phenotype can be caused by faulty centrosomes and/or cilia (33). Additionally, we observed a tendency for pericardial edema (Figure 1F) suggesting a potential defect in heart development, a common feature in patients with situs anomalies caused by cilia dysfunction (34,35). To assess a potential involvement of zebrafish Mcm2 in cilia-dependent processes, we examined the direction of heart looping. Normally, the two-chambered heart of 2 days old zebrafish embryos assumes a S-shape with the ventricle being left from and slightly above the atrium. When bilateral asymmetry due to cilia dysfunction is disturbed, ∼50% of the treated embryos are expected to develop either inversely looped hearts or hearts that fail to undergo looping (Figure 2A). We observed that both MOs targeting Mcm2 rendered embryos with random heart looping (Figure 2A), while injection of five base mismatch control MOs produced embryos similar to uninjected wild-type controls. Moreover, Mcm2 knockdown similarly randomized placement of the pancreas (Figure 2B). Depletion of Mcm2 also yielded a significant number of embryos with ambiguous *southpaw* (*spaw*) expression in the lateral plate mesoderm (Figure 2C). *Spaw* is one of several leftward determining genes of the nodal cascade, which determines the oriented development of internal organs and is commonly used as readout for left-right asymmetry during early stages (36). Since *spaw* distribution was partially rescued by co-injection of capped RNA encoding *mcm2* (Figure 2C), we conclude that Mcm2 helps to control left-right asymmetry development in zebrafish embryos. This function, however, does not apply to DNA helicases in general. Inhibition of Bloom helicase, which is involved in DNA repair processes, by a small molecule inhibitor (37) did not affect lateralization (Figure 2D). These data suggest that loss of Mcm2 likely confers cilia dysfunction in zebrafish.

### Mcm2 facilitates cilium formation and function

**Figure 3.**
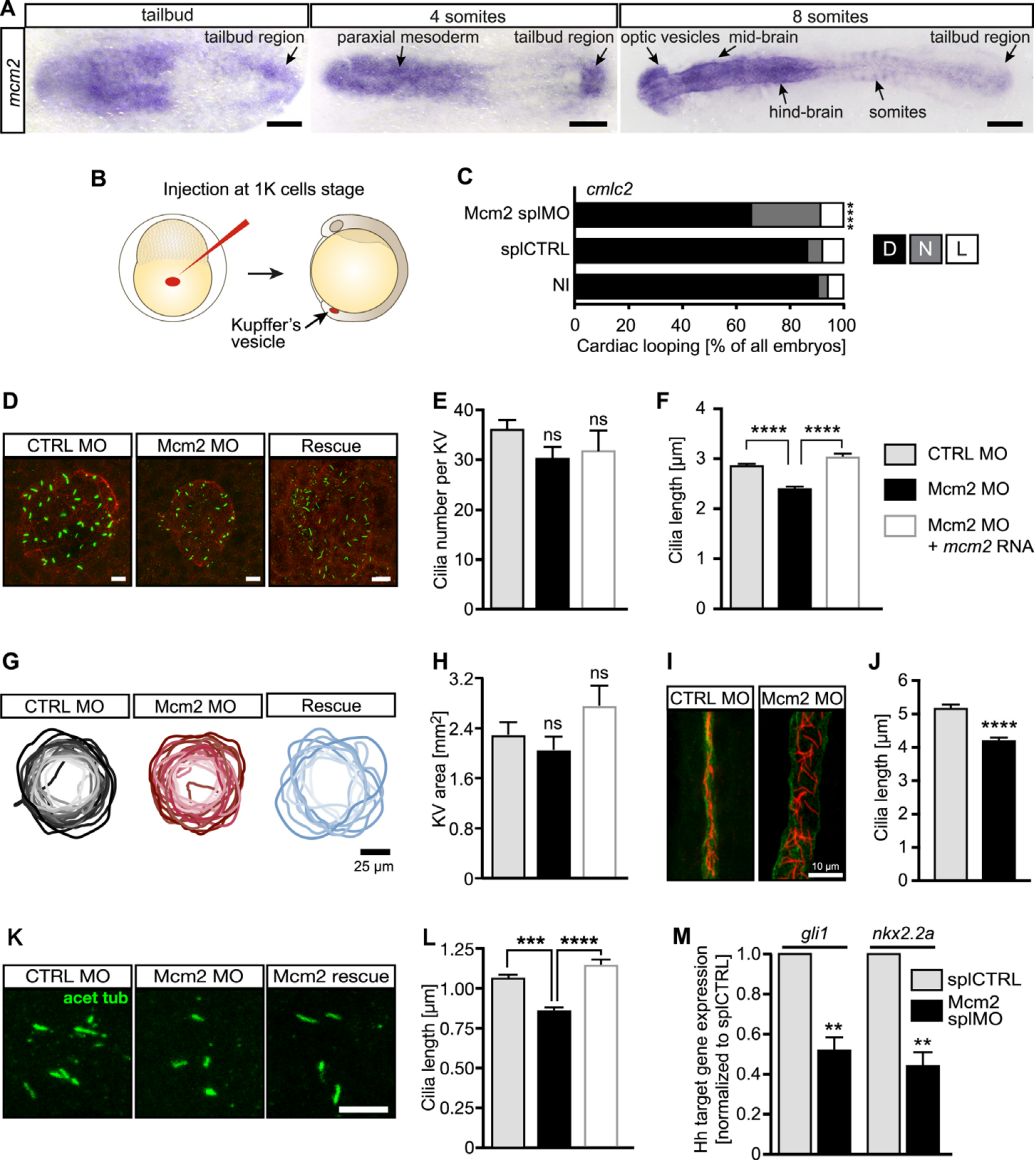
Impaired left-right asymmetry development upon Mcm2 knockdown is accompanied by shorter cilia. (**A**) Expression of *mcm2* in the tailbud of zebrafish embryos. Scale bars: 150 μm (tailbud and 4ss), 200 μm (8 ss). (**B**) Injection strategy to target KV cells. (**C**) Randomized heart looping after KV-specific Mcm2 ablation. *P* = 0.0007, two-tailed Fisher's exact test. n = 4 experiments. Number of embryos: NI = 121; splCTRL = 115; Mcm2 splMO = 118. (**D**) Confocal stacks of motile cilia (green) in the KV of 6–8 ss embryos injected with Mcm2 control or Mcm2 translation blocking MO. Cilia were labelled using an anti-acetylated tubulin antibody. Apical cell borders for visualization of the KV area were stained with an anti-PKCζ antibody (red). Scale bar: 10 μm. (**E**) Cilia numbers are not changed in the KV of Mcm2 morphants. *P* = 0.1403 (CTRL MO vs Mcm2 MO), p>0.9999 (Mcm2 MO/Ctrl Mo vs Rescue). Kruskal–Wallis test with Dunn's multiple comparisons test. In four experiments 29 CTRL MO, 28 Mcm2 MO and 12 Rescue KVs were assessed. (**F**) Depletion of Mcm2 results in shorter KV cilia, which can be rescued by co-injection of MO-insensitive RNA encoding Mcm2. *P* < 0.0001. Kruskal–Wallis test with Dunn's multiple comparisons test. *n* = 4 with 920 CTRL MO and 870 Mcm2 MO and 243 Rescue cilia. (**G**) KV outlines at 6–8 ss. Darker colour reflects larger areas. Scale bar: 25 μm. (**H**) KV is not significantly altered upon Mcm2 knockdown or its rescue. *n* = 19 CTRL MO, 22 Mcm2 MO and 10 Rescue KVs. *P* = 0.7952 (Ctrl MO vs Mcm2 MO), *P* = 0.1425 (Mcm2 MO vs Rescue). Kruskal–Wallis test with Dunn's multiple comparisons test. (**I**) Confocal stacks of immunostained pronephric ducts (cilia: acetylated tubulin, red, duct: PKCζ, green) at 24 hpf. Dilatation of the pronephric duct as well as disorganization of cilia was seen in the majority of Mcm2 depleted zebrafish. Scale bar: 10 μm. (**J**) Distal cilia length in the pronephros is reduced upon Mcm2 knockdown. *n* = 10 CTRL MO and 8 Mcm2 MO embryos with 368 and 299 cilia, respectively. *P* < 0.0001. Two-tailed Mann–Whitney test. (**K**) Primary cilia in the tailbud of 6–8 ss embryos. Scale bar: 5 μm. (**L**) Primary cilia length is reduced in the presence of Mcm2 MO and restored, when co-injected with RNA encoding Mcm2. *n* = 375 (CTRL MO), 413 (Mcm2 MO) and 171 (Mcm2 rescue) cilia. *P* < 0.0001. Kruskal–Wallis test with Dunn's multiple comparisons test. (**M**) Hh pathway activity is dampened in Mcm2 depleted zebrafish as shown for the Hh target genes *gli1* and *nkx2.2a. P* = 0.0018 (*gli1*) and *P* = 0.0013 (*nkx2.2a*), unpaired, two-tailed *t*-test with Welch's correction. *n* = 5 experiments.

Currently, the best-understood and most widely accepted mechanism of situs development relies on the proper formation and function of motile cilia in the Kupffer's vesicle (KV), the temporal organizer of left-right asymmetry in zebrafish (4). Cilia are tubulin-based, hair-like cell organelles that emanate from the surface of most post-mitotic vertebrate cells (2). In the adult organism, though, MCM2 is expressed in cycling rather than quiescent cells (38) which argues against a role of MCM2 in cilia. Yet, during development Mcm2 is also expressed during stages when differentiation and cell migration are more prominent than proliferation (39) (Figure 3A). Moreover, we detected *mcm2* transcripts in ciliated tissues such as the brain, ear and pronephros (Supplementary Figure S1). We thus examined whether the hitherto observed situs anomaly phenotypes are due to a direct function of Mcm2 on cilia. We found *mcm2* was expressed in the tailbud (Figure 3A), where the KV forms and left-right symmetry breaking is controlled by motile cilia (5,40). To specifically target ciliated cells of the KV, we performed tissue-specific knockdown of Mcm2 by MO injection into the yolk at the 1000 cell stage (13) (Figure 3B). This KV-specific knockdown of Mcm2 affected heart looping to a similar extent (Figure 3C) as in the ubiquitous knockdown experiments (Figure 2A). As this suggested a cilium defect in the KV we analysed cilia in zebrafish embryos and observed that depletion of Mcm2 significantly shortened cilia in the KV without affecting the overall number of cilia (Figure 3D–F). Co-injection of capped RNA encoding Mcm2 fully rescued cilium length in Mcm2 morphants (Figure 3F). We also measured the area of the KV as a smaller vesicle could potentially account for smaller cilia. KV size, however was not significantly changed upon Mcm2 knockdown or in the rescue condition (Figure 3G and H).

In addition to KV cilia, we analysed cilia in other tissues of the embryo. Motile cilia in the pronephric duct of Mcm2 knockdown embryos were shorter, although the duct was heavily dilated (Figure 3I and J). Mcm2 knockdown resulted furthermore in shorter primary cilia in the tissue surrounding the KV, which could be rescued by reconstitution with Mcm2 (Figure 3 K and L). Since cilium length determines also the signalling abilities of primary cilia we assessed Hedgehog signalling, which relies on the primary cilium (41). Normally, the amplitude of Hedgehog signalling is directly correlated to cilia assembly, meaning that shorter cilia result in decreased capacity for Hedgehog signalling activity (42). This can be assessed using qPCR for the target genes *nkx2.2* and *gli1* (43). We hence isolated RNA from 24 hpf control and Mcm2 knockdown fish, transcribed equal amounts into cDNA and performed qPCR. Depletion of Mcm2 produces zebrafish embryos with dampened Hedgehog signalling (Figure 3M), consistent with the observed decrease in primary cilium length. These results together suggest that Mcm2 facilitates formation and hence proper function of motile and primary cilia.

### MCM2 prevents cilium dysfunction and centrosome aberrations in human fibroblasts

**Figure 4.**
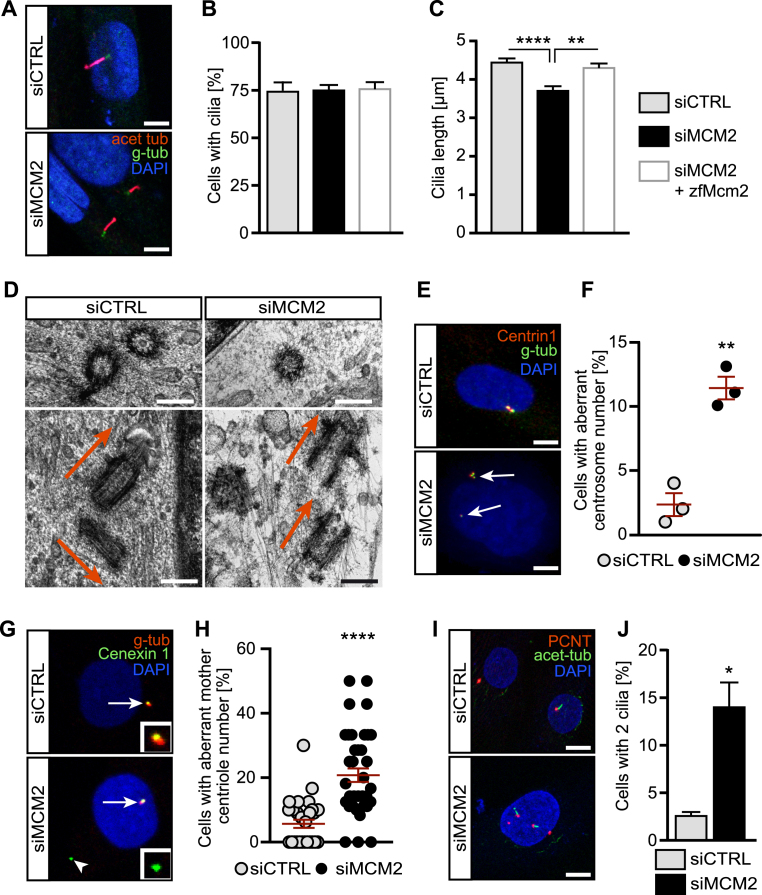
MCM2 controls the formation and function of primary cilia and prevents centrosome amplification. (**A**) Shorter cilia in hTert 1BR3 cells transfected with MCM2 siRNA using liposomes compared to cells transfected with control siRNAs. Scale bar: 5 μm. (**B**) Primary cilia form at similar rates upon nucleofection with CTRL siRNA, MCM2 siRNA or MCM2 siRNA plus a plasmid encoding zebrafish Mcm2. *P* >0.9999, Kruskal–Wallis test. *n* = 3 transfections. 100 cells were counted in each condition per transfection. (**C**) Primary cilia are shorter in the absence of MCM2. This can be rescued by zebrafish Mcm2 co-nucleofection. **P = 0.0069, ****P < 0.0001, Kruskal–Wallis test with Dunn's multiple comparisons test. *n* = 94 (siCTRL+empty vector), 92 (siMCM2+empty vector) and 93 cilia (siMCM2+zfMcm2) cilia from three nucleofections. (**D**) Cryo-EM pictures of centrosomes in control and MCM2 KD cells. Transverse section (upper row) shows a singlet centriole after MCM2 knockdown. Longitudinal sections (lower row) shows correct perpendicular orientation of the centrioles in control cells, while MCM2 knockdown cells show two mother centrioles in parallel to each other. Scale bar: 250 μm. (**E**) Centrin1 staining reveals supernumerary centrosomes after MCM2 knockdown. Scale bar: 10 μm. (**F**) Quantification of aberrant centrosome numbers in control and MCM2 siRNA transfected cycling cells. *n* = 3 three transfections. Per condition and transfection 100 cells were counted. *P* = 0.0019. Unpaired, two-tailed *t*-test with Welch's correction. (**G**) Additional centriolar material (arrowhead) in MCM2 depleted cells is Cenexin 1 positive, which indicates mother centrioles. Gamma-Tubulin was used to counterstain centrioles. Arrow: regular centrosome. Inset: higher magnification of Cenexin1^+^ material. (**H**) Percentage of cells with additional mother centrioles. *P* < 0.0001, unpaired, two-tailed Mann–Whitney test. Number of cells: siCTRL = 325 cells; siMCM2 = 314 cells (three experiments). (**I**) MCM2 depletion induces the formation of more than one primary cilium per cell. Immunofluorescence of cycling cells stained for PCNT and acetylated tubulin. Scale bar: 10 μm. (**J**) Percentage of ciliated cells with two cilia. *P* = 0.0111, unpaired, two-tailed *t*-test with Welch's correction. Number of cells: siCTRL = 625 cells; siMCM2 = 495 cells (*n* = 5).

To test whether this new asset of Mcm2 applied similarly to primary cilia in other organisms we depleted human fibroblasts of MCM2 using RNA interference via transfection of pools of siRNAs. First, we used a liposome-based transfection method. Knockdown efficiency was demonstrated by qPCR and Western blot (Supplementary Figure S2A and B). As a third means of knockdown validation we assessed the cell cycle profile upon MCM2 knockdown. Previously, it had been shown that cells with reduced MCM2 levels have a G2/M arrest with a decrease in S phase cells (44). Consistently, transfection of siRNA against MCM2 resulted in fewer S phase cells and more G2/M phase cells (Supplementary Figure S2C). Three days of serum starvation, which is needed to induce ciliogenesis, efficiently drove the majority of MCM2 knockdown cells into quiescence, although a small percentage of G2/M phase cells resisted (Supplementary Figure S2D). Despite the imperfect synchronization of MCM2 knockdown cells upon serum starvation, ciliation frequency did not differ from control cells (Supplementary Figure S2E). Cilia length, though, was significantly reduced ((Figure 4A, Supplementary Figure S2F) as in the different zebrafish tissues. To make sure that the measured decrease in cilium length was not due to incompletely retracted cilia remaining from the previous G0 phase, we assessed the length distribution of control and siMCM2 transfected cells. Since we did not see two populations of differently sized cilia, but rather a normally distributed, left shifted curve compared to controls (Supplementary Figure S2G) we conclude that MCM2 likely controls cilia length also in G0 phase human fibroblasts. These cilia are furthermore impaired in ciliary Hedgehog signalling. Stimulation of serum starved cells with SAG resulted in robust expression of the Hedgehog target gene *GLI1* in siCTRL cells. SAG is an agonist for Smoothened, which is the signal transducing molecule in Hedgehog signalling. SAG stimulation drives Smoothened translocation into the cilium and hence activates the cascade (41,45). In cells transfected with siMCM2 SAG stimulation failed to induce target gene expression (Supplementary Figure S3).

In order to verify that this effect was specific and due to MCM2 depletion, we performed rescue experiments. Since 1BR3 fibroblasts are difficult to transfect we had to change to a nucleofection-based approach to allow simultaneous transfection of siRNA smartpools and a plasmid encoding zebrafish Mcm2 or empty vector. We achieved robust knockdown of MCM2, albeit to a lesser extent compared to liposome-based transfection (Supplementary Figure S4A). Expression from the nucleofected plasmid was also verified (Supplementary Figure S4B). Here again, we observed no changes in the percent of cells forming cilia (Figure 4B), however cilium length was significantly reduced upon loss of MCM2 and restored upon expression of zebrafish Mcm2 (Figure 4C).

Next, we tested whether MCM2 ablation would affect centrosomes, not least because MCM2 has been detected at centrosomes (46). Moreover, MCM5 and components of the origin recognition complex have also been reported to localize to and control stability of centrosomes (47,48). Centrosomes are tubulin-rich structures that consist of a mother and a daughter centriole (6). In cycling cells centrosomes facilitate mitosis, while in quiescent cells the mother centriole docks to the inside of the plasma membrane using distally located appendages and becomes the basal body from which the cilium extends (49). Thus, proper centriole or centrosome assembly is necessary for nucleation of cilia, which is reflected by the substantial overlap of clinical features of ciliopathies with those of syndromes with centrosomal involvement (6). Using cryo-electron microscopy we found singlet centrioles in siMCM2 transfected cells, which we never observed in control transfected cells (Figure 4D). Control cells furthermore displayed perpendicular orientation of mother and daughter centriole to each other while we found parallel arrangement of centrioles in MCM2 knockdown cells. Moreover, instead of having one mother and one daughter centriole, two mother centrioles apparent by their appendages could be observed (Figure 4D). We therefore assessed mature centriolar material in cycling hTERT-immortalized fibroblasts using Centrin and Pericentrin (PCNT) as markers. Cells depleted of MCM2 displayed an increase in supernumerary centrosomes and pericentriolar material compared with control cells (Figure 4E and F and Supplementary Figure S5). Interestingly, using Cenexin 1 staining that specifically labels mother centrioles (50) we identified the additional centrioles as mother centrioles (Figure 4G and H). We further detected an increase in cells containing two rather than a single cilium when MCM2 was knocked down (Figure 4I and J). Extra cilia have been reported to arise as a consequence of supernumerary centrosomes and were shown to result in cilia dysfunction (51).

### Genes regulating cilia and centrosomes are dysregulated in cells lacking MCM2

**Figure 5.**
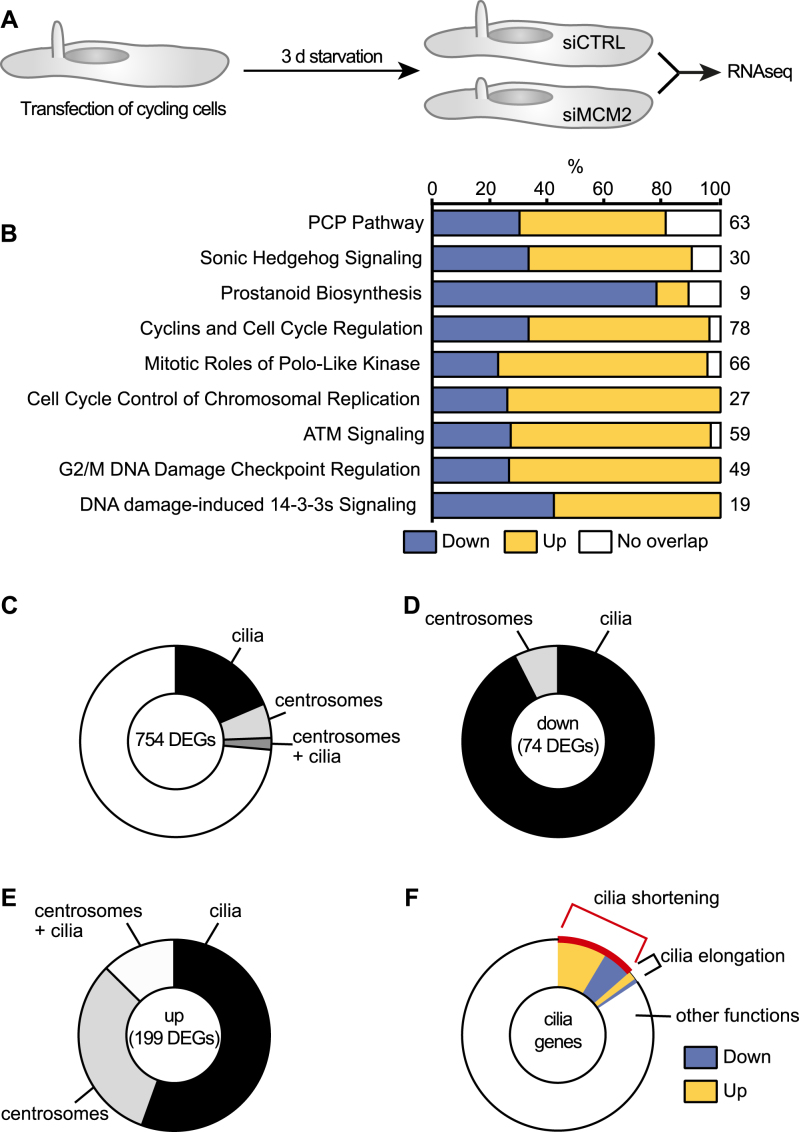
MCM2 knockdown alters expression of genes associated with cilia and centrosomes. (**A**) Cycling hTert fibroblasts were transfected with siRNAs and ciliation was induced by serum starvation. RNAseq was performed as described in the methods section. *n* = 3. (**B**) Pathway analysis for differentially regulated genes (DEGs, cut off: ≥1.5-fold up or down) using Ingenuity Pathway analysis. (**C**) Pie chart showing the percentage of DEGs associated with cilia, centrosomes or both structures according to literature. Only genes with ≥1.5 fold regulation and *P* < 0.05 were considered. (**D**) The majority of downregulated DEGs associated with cilia or centrosomes are cilia genes. (**E**) The majority of upregulated DEGs associated with cilia or centrosomes are cilia genes. (**F**) Of all 199 DEGs linked to cilia, around 15% have been reported to regulate cilia length. Here, the red-labelled portion indicates genes, which have been reported to shorten cilia, when regulated as in this RNAseq analysis.

MCM2 is primarily a replication protein, but it also binds to RNA polymerase II (52). We therefore reasoned that MCM2 may have an impact on RNA levels of ciliogenesis genes. Moreover, replication and transcription are often coupled (53). Hence, we performed RNA sequencing on ciliated human fibroblasts that were either transfected with control siRNAs or siMCM2 and subsequently serum-starved for three days (Figure 5A, Supplementary Figure S2A, B and D). This resulted in 2360 differentially expressed genes (DEGs; Supplementary Figure S3B), of which 754 were at least 1.5-fold up- or downregulated. Pathway analysis of DEGs revealed changes in ciliary signalling cascades such as the planar cell polarity pathway (PCP), prostanoid signalling or Hedgehog signalling (Figure 5B). Additionally, cell cycle control as well as DNA damage response pathways were altered. The latter has been linked to centrosomes and with that to cilia (54), although the underlying mechanism has remained elusive. In addition, recently a role for the MCM complex in DNA repair has been identified (55,56).

We searched these 754 genes one-by-one against the cilia database Cildb and used PubMed, Genecards and Google to identify links to cilia or centrosomes (57,58). We found that more than 26% of the DEGs associated with cilia and/or centrosomes (Figure 5C, Supplementary Tables S2–S4). Cilia genes were more represented than centrosome associated genes or genes that could be linked to cilia and centrosomes (Figure 5D and E). We next classified all regulated cilia genes according to their function. For the majority only expression data (i.e. the presence in cilium proteomes) could be found, but for some cilia genes we found a correlation to cilium length, for example some upregulated genes functioned in cilium disassembly. Similarly, for many downregulated DEGs, reports could be found that loss of these DEGs results in shorter cilia (Figure 5F), which was consistent with our observation of shorter cilia upon MCM2 knockdown. Furthermore, centrosomal DEGs indicated upregulation of genes involved in centrosome duplication or centrosome splitting, which supports our observation of additional centriolar and pericentriolar material in MCM2 knockdown cells. For instance, upregulation of PLK1 has been shown to induce mother centriole duplication (59) and could account for the presence of extra cilia in fibroblasts. Hence, RNAseq suggests that MCM2 influences the expression of genes involved in ciliogenesis and centrosome duplication.

### MCM2 functions in non-replicating cells

**Figure 6.**
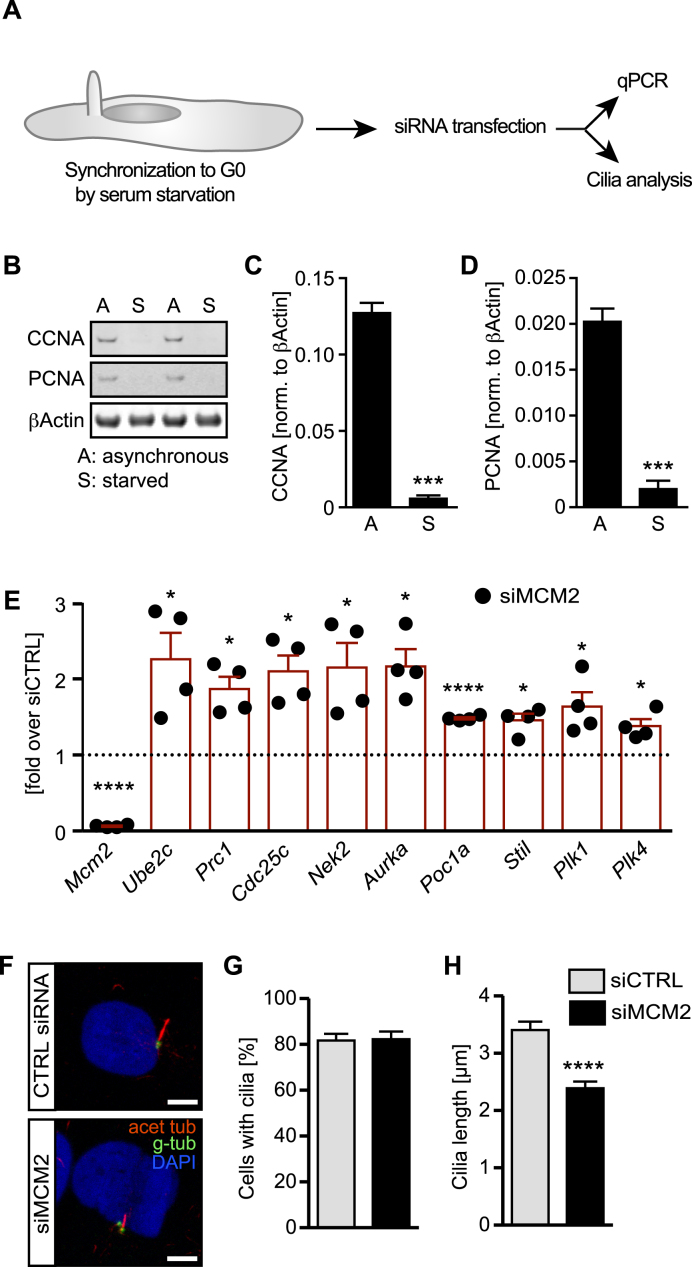
The impact of MCM2 on cilia occurs also in non-replicating cells. (**A**) siRNA transfections were carried out after 1 day or serum starvation. Then, transcriptional changes indicative of cilia and centrosome impairment were monitored and cilia length was measured. (**B**) Human fibroblasts serum starved for 24 hours have ceased to express markers indicative of S-phase (Cyclin A, CCNA) or cycling cells (PCNA). Western blot of cells cultured in regular serum-containing medium (A) and of cells starved for 24 h. (**C**) Quantification of CCNA expression (normalized to βActin) in cells cultured either in full (A) or serum-free medium (S). *n* = 4. (**D**) Quantification of PCNA expression (normalized to βActin) in cells cultured either in full (A) or serum-free medium (S). *n* = 4. (**E**) MCM2 siRNA transfection in cells serum-starved for 24 hours significantly upregulates genes implicated in cilia shortening and centriole duplication. Expression levels were normalized to levels in siCTRL transfected cells (indicated by dashed line intersecting the y axis at 1). *n* = 4, paired, two-tailed *t*-test with Welch's correction. *Mcm2*: *P* < 0.0001; *Ube2c*: *P* = 0.0357; *Prc1*: *P* = 0.0123; *Cdc25c*: *P* = 0.0134; *Nek2*: *P* = 0.0322; *Aurka*: *P* = 0.0110; *Poc1a*: *P* < 0.0001; *Stil*: *P* = 0.0133; *Plk1*: *P* = 0.0437; *Plk4*: *P* = 0.0246. (**F**) MCM2 knockdown in non-replicating cells shortens cilia. Scale bar: 5 μm. (**G**) Primary cilia form at similar rates upon MCM2 knockdown in non-replicating cells. *P* = 0.8957, unpaired, two-tailed t-test with Welch's correction. Number of cells: siCTRL = 138 cells; siMCM2 = 146 cells (*n* = 3 experiments). (**H**) Primary cilia are shorter upon MCM2 depletion in G0. *P* < 0.0001, unpaired, two-tailed *t*-test with Welch's correction. 3 experiments. *n* = 71 cilia (siCTRL) and 69 cilia (siMCM2) from three transfections.

MCM2 is primarily a replication-associated gene and thus functions canonically in cycling cells. Cilia, however, form only in cells not undergoing division (1). We therefore tested whether MCM2’s impact on ciliogenesis can be uncoupled from its canonical function. To do so, we transfected siRNAs into cells that were serum starved 24 h prior to siRNA pool transfection and hence should have ceased to undergo active replication and proliferation (Figure 6A). This was confirmed by Western blot analysis for the S-phase protein Cyclin A and the interphase marker PCNA, which demonstrated that serum starvation for 24 h efficiently synchronized cells into a non-replicating state (Figure 6B–D). After continuous starvation to reliably induce cilium formation we assessed the expression of selected DEGs causing cilia shortening, cilia disassembly and centrosome overduplication as identified during RNAseq and cilia length. qPCR analysis revealed the same upregulation as after MCM2 knockdown in interphase cells (Figure 6E). Furthermore, knockdown of MCM2 under these conditions efficiently reduced cilia length demonstrating that any potential impairments arising during replication are unlikely to be the sole cause of the cilia defects observed upon MCM2 depletion (Figure 6F–H).

### MCM2 occupies transcription start sites of genes regulating centrosomes and ciliogenesis

**Figure 7.**
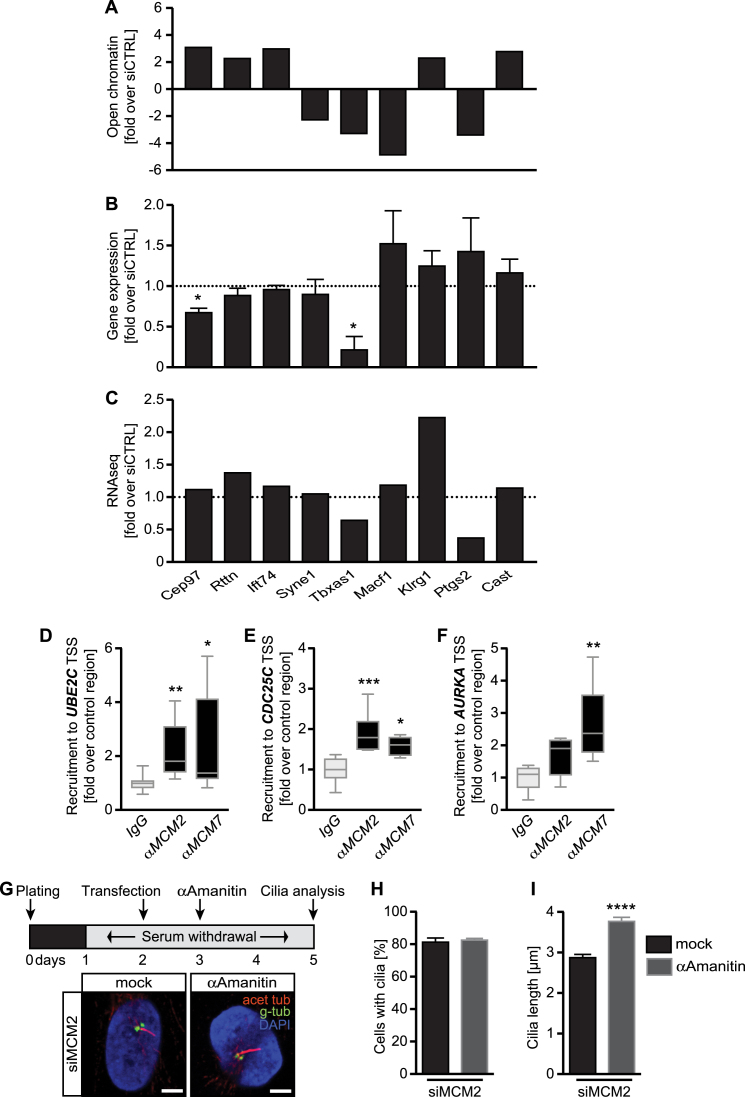
MCM2 promotes ciliogenesis by restraining of transcription. (**A**) ATACseq of G0 cells reveals subtle changes in open chromatin in close proximity to genes implicated in ciliogenesis in siMCM2 transfected fibroblasts compared to siCTRL transfected cells *n* = 3 experiments. For identification of bars please refer to labels in C. (**B**) Fibroblasts were transfected with siRNAs and ciliogenesis induced by serum starvation. The qPCR analysis was performed for genes, which appeared to be more or less accessible for transcription (A). *n* = 3–8 experiments. *P* = 0.0199 (*Cep97*) and 0.0156 (*Tbxas1*), paired *t*-test. For identification of bars please refer to labels in **C**. (**C**) Bar graph showing RNAseq results of the same genes as in A and B. (**D–F**) MCM2 and MCM7 are present at transcription start sites (TSS) of genes regulating ciliogenesis. Chromatin immunoprecipitation with antibodies against MCM2 and MCM7 reveal enrichment of DNA fragments corresponding to transcription start sites of genes differentially expressed in our RNAseq analysis as compared to the IgG control. Shown are results normalized to the input fraction and to a control region upstream of the analysed gene. *n* = 5-11, Kruskal-Wallis test with Dunn's multiple comparison test. *Ube2c*: *P* = 0.0079 (MCM2) and 0.0281 (MCM7); *Cdc25c*: *P* = 0.0004 (MCM2) and 0.0246 (MCM7); *Aurka*: *P* = 0.1477 (MCM2) and 0.0034 (MCM7) (MCM2 or 7 ChIP compared to IgG ChIP). (**G**) Unleashed transcription upon MCM2 knockdown in G0 cells can be prevented by inhibition of RNA polymerase II. Transfected cells were treated with the RNA polymerase II inhibitor α-amanitin during ciliogenesis. Confocal analysis reveals longer cilia upon RNA polymerase II inhibition. Scale bar: 5 μm. (**H**) Primary cilia form at similar rates upon α-amanitin treatment in MCM2 knockdown cells. *P* = 0.6023, unpaired, two-tailed t-test with Welch's correction. Number of cells: mock = 324 cells; α-amanitin = 336 cells (*n* = 3 experiments). (**I**) RNA polymerase II inhibition rescues the short cilia phenotype upon loss of MCM2. *P* < 0.0001, unpaired, two-tailed Mann–Whitney test. Three experiments. *n* = 90 cilia (mock) and 93 cilia (α-amanitin) from three transfections.

In order to find out how MCM2 impacts on transcription of genes required for cilia formation we assessed nucleosome repositioning, which can depend on MCM2 (60) and which would make gene loci required for cilia and centrosomes more accessible for transcription. We performed ATAC-seq on serum-starved siCTRL or siMCM2 fibroblasts. Interestingly, we did not observe gross changes in open chromatin but found a small number of loci connected to cilia or centrosomes more accessible (Figure 7A and Supplementary Table S5). Interestingly though, expression of the majority of these genes did not correspond to the changes detected by ATAC-seq (Figure 7B) and neither did we see the same changes when we compared the results to our RNA sequencing (Figure 7C). We therefore examined whether MCM2 might have a more direct influence on transcription of cilia and centrosome-related genes and performed chromatin immunoprecipitation (ChIP) for three of the most upregulated genes according to our RNAseq experiment. As a control we used MCM7 ChIP since MCM7 has recently been shown to occupy genomic regions upstream of transcription start sites of active genes (61). We found MCM2 and MCM7 at transcription start sites of Aurora kinase A, CDC25C and UBE2C (Figure 7D–F). Loss of UBE2C results in longer cilia (62), while excessive Aurora kinase A promotes cilia disassembly as well as centrosome amplification (63,64). Similarly, CDC25C has been shown to localize to centrioles and provide a permissive condition for centrosome overduplication (65). The extent of DNA occupancy correlated with the expression of AURKA, CDC25C and UBE2C upon MCM2 knockdown (Figure 7D–F) suggesting an inhibitory action of MCM2 on the expression of these genes and potentially all other DEGs obtained by RNAseq (Figure 6). Additional consensus analysis of the transcription start sites and promotors found enrichment of CpG islands in cilia- and centrosome-related RNAseq-DEGs compared to non-regulated genes (1.75-fold enrichment, *P* = 6.7e–07) and a seven nucleotide consensus sequence (Supplementary Figure S7), which supports the hypothesis of MCM2 selectively acting on genes hindering cilium extension.

We thus speculate that MCM2 occupies transcription start sites of genes, which (negatively) regulate cilium length. Loss of MCM2 unleashes transcription of these genes resulting in shorter cilia. The prediction of this hypothesis is that mild inhibition of RNA polymerase II-mediated transcription in G0 would rescue the cilia defect. We performed again MCM2 knockdown in cells following 24 h serum starvation and treated with low concentrations of the RNA polymerase II inhibitor α-amanitin during continuous starvation (Figure 7G). While this did not affect ciliation per se (Figure 7H), we observed that such dampening of transcription restored cilia length in MCM2 knockdown cells (Figure 7I).

### Knockdown of Mcm7 produces overlapping cilia dysfunction phenotypes as observed following Mcm2 knockdown

**Figure 8.**
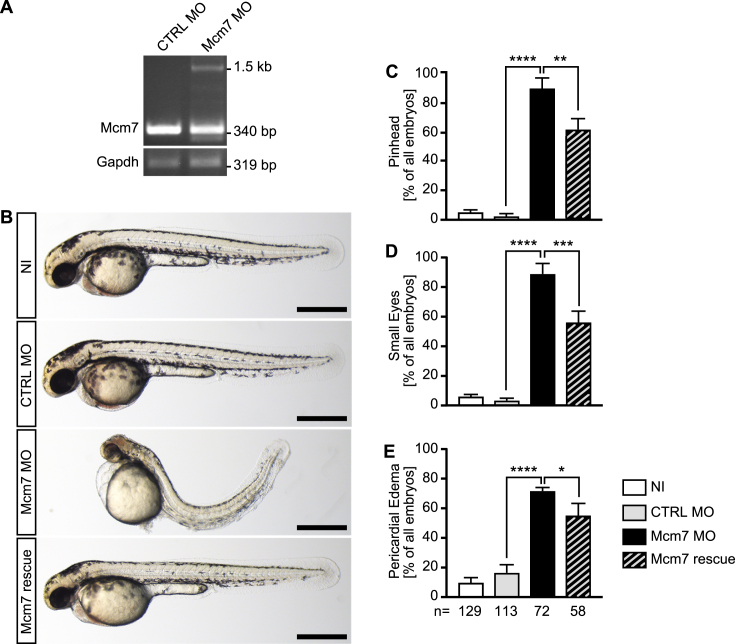
Mcm7 knockdown in zebrafish. (**A**) RT-PCR of control injected (CTRL MO) and Mcm7 MO injected embryos at 24 hpf. In embryos with Mcm7 MO the original band at 340 bp partially disappeared. Instead a second band around 1500 bp could be detected, which contained intronic sequences. *n* = 3 independent experiments. (**B**) Live images of zebrafish at 48 hpf. Scale bars: 500 μm. (**C**) Mcm7 depleted embryos develop smaller anterior structures, which is partially rescued by co-injection of RNA encoding human MCM7. Five independent experiments. **P < 0.01, ****P < 0.0001. One-way ANOVA with Holm–Sidak's multiple comparison test. (**D**) Loss of Mcm2 impairs eye development. Five independent experiments. ***P < 0.001, ****P < 0.0001. One-way ANOVA with Holm–Sidak's multiple comparison test. (**E**) Zebrafish lacking Mcm7 develop pericardial edema. 5 independent experiments. *P < 0.05, ***P < 0.0001. One-way ANOVA with Holm–Sidak's multiple comparison test. (**C–E**) Numbers of embryos analysed are given below the bars in E.

**Figure 9.**
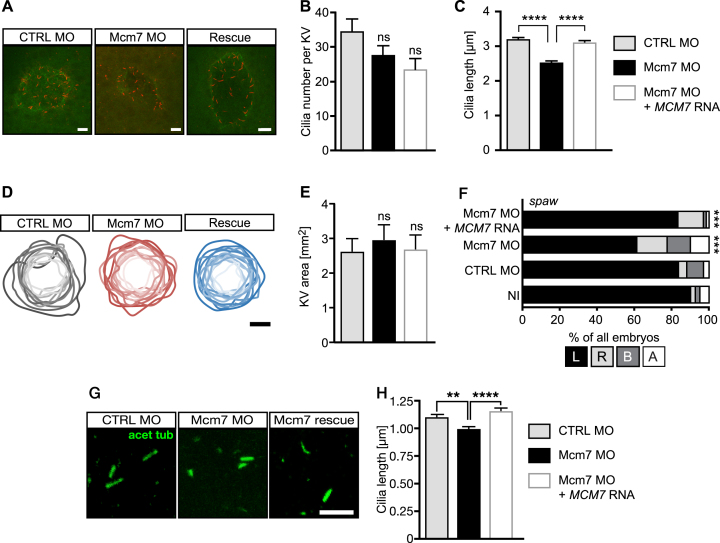
Loss of MCM7 impairs cilia in zebrafish. (**A**) Confocal stacks of zebrafish KVs at 8ss. Cilia in red (acetylated tubulin) and apical borders of KV in green (PKCζ). Scale bar: 5 μm. (**B**) Cilia number is not significantly changed upon loss of Mcm2, but shows a tendency towards fewer cilia. Rescue embryos were consecutively injected with Mcm7 MO and capped RNA for human MCM7, which is insensitive to the MO. N = 14 (CTRL MO), 18 (Mcm7 MO) and 16 (Mcm7 MO+MCM7 RNA) KVs. *P* = 0.4035 (CTRL MO vs Mcm7 MO), 0.7541 (Mcm7 MO vs Rescue) and 0.0873 (CTRL MO vs Mcm7 Rescue). One-way ANOVA with Sidak's multiple comparison test. (**C**) Mcm7 knockdown leads to shorter KV cilia. Co-injection of RNA for human MCM7 rescues cilium length. *P* < 0.0001. Kruskal–Wallis test with Dunn's multiple comparisons test. *n* = 482 CTRL MO, 480 Mcm2 MO and 355 Rescue cilia. (**D**) Manipulation of Mcm7 levels does not change the area of the KV. Cartoon displays outlines of individual KVs with darker shading indicating larger areas. Scale bar: 25 μm. (**E**) Bar graph summarizing KV area measurements in control conditions and upon Mcm7 knockdown and rescue thereof. N = 14 (CTRL MO), 11 (Mcm7 MO) and 11 (Mcm7 MO+MCM7 RNA) KVs. *P* = 0.9269 (CTRL MO vs Mcm7 MO or Rescue). One-way ANOVA with Holm–Sidak's multiple comparison test. (**F**) The laterality gene *southpaw* (*spaw*) loses its leftward restriction upon knockdown of Mcm7 (22 ss). Reconstitution with human MCM7 restores expression left from the midline. ***P < 0.001 (CTRL MO vs Mcm7 MO and Mcm7 MO vs Mcm7 MO+*hMCM7*), two-tailed Fisher's exact test. Number of embryos: NI = 81; CTRL MO = 67; Mcm7 MO = 80, Mcm7 MO+*hMCM7* = 66. (**G**) Confocal z-stacks of primary cilia in the tailbud of 8 ss embryos. Scale bar: 5 μm. (**H**) Knockdown of Mcm7 reduces primary cilium length in vivo. Co-injection of RNA for human MCM7 rescues the defect. *n* = 382 (CTRL MO), 334 (Mcm7 MO) and 272 (Mcm7 rescue) cilia. **P=0.0046, ****P < 0.0001. Kruskal–Wallis test with Dunn's multiple comparisons test.

MCMs within the MCM2–7 complex possess distinct activities. MCM2, which we have analysed here, possesses a regulatory role on the DNA unwinding ability of the complex, while MCM4 and 7 are the components with the actual helicase activity (66). To extend our study, we cloned the open reading frame of zebrafish Mcm7 after 5′-RACE PCR from 13 somites stage zebrafish embryos. Using this sequence we generated a DIG-labelled riboprobe and performed *in situ* hybridization on zebrafish embryos. We found that Mcm7 is expressed in the same tissues during development as Mcm2 (Supplementary Figure S8). Injection of a MO against Mcm7, which resulted in retention of intronic sequences and hence a premature stop codon (Figure 8A) rendered embryos with a ciliopathy-like phenotype, namely a distinct body curvature, pinheads, small eyes and pericardial edema (Figure 8B). As for Mcm2, similar defects have been documented for the hi2704 mutant of Mcm7 (32). Co-injection of RNA encoding human MCM7 partially rescued these phenotypes (Figure 8C–E). Surprisingly, analysis of KVs revealed a tendency towards fewer cilia upon loss of Mcm7 and even fewer upon reconstitution with MCM7, although not in a statistically significant manner (Figure 9A and B). Similar to Mcm2 knockdown embryos, Mcm7 depletion significantly reduced cilium length in the KV, which could be rescued by human MCM7 (Figure 9C). The size of the KV was not changed (Figure 9D and E).

Consistent with the change in cilium length we also observed randomization in the expression domain of *spaw*, which should be exclusively in the left side of the body. Upon knockdown of Mcm7 many embryos expressed *spaw* on the right side or ambiguously. Again, RNA for human MCM7 was able to rescue this laterality defect (Figure 9F). In addition, we measured primary cilia length in the tailbud of 8 somites stage embryos and observed a reduction in the absence of Mcm7 and complete rescue upon co-injection of *MCM7* RNA (Figure 9G and H). Taken together, embryos depleted of Mcm7 develop cilia defects, similar to that observed following Mcm2 depletion.

### MCM7 is required for cilium formation in human fibroblasts

**Figure 10.**
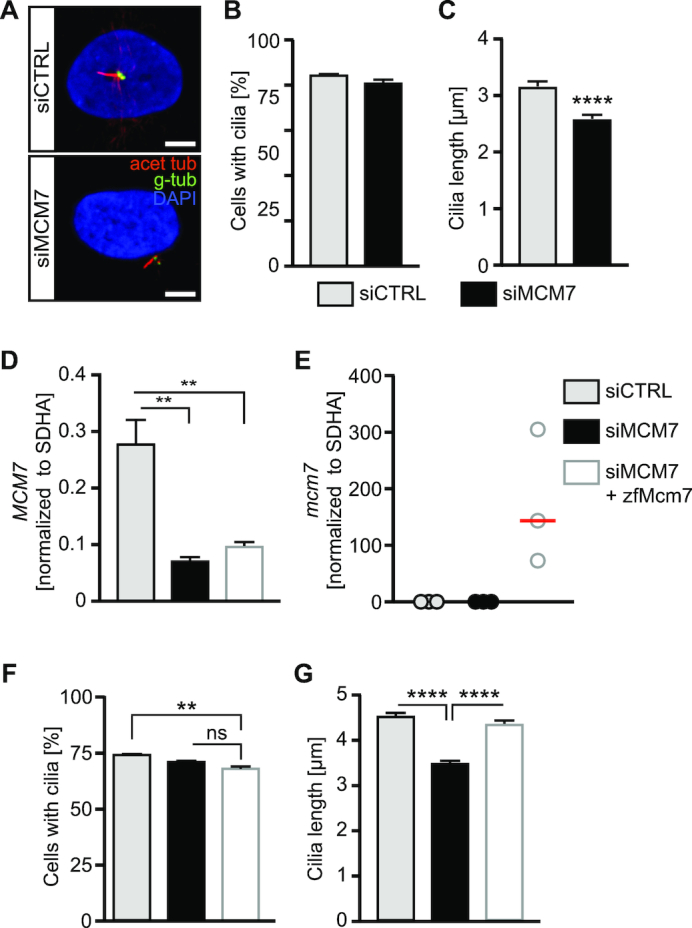
Knockdown of MCM7 in human fibroblast reduces cilium length. (**A**) Confocal stacks of cilia (red, acetylated tubulin; basal body and daughter centriole in green, gamma tubulin) in hTert 1BR3 cells transfected with control siRNA and MCM7 siRNA, respectively. Cells were transfected under cycling conditions and after 48 h serum starved for another three days. Scale bar: 5 μm. (**B**) Similar ciliation frequency in siCTRL and siMCM7 transfected fibroblasts. *n* = 3 transfections. Per experiment and condition 100 cells were counted. (**C**), Shorter cilia upon siRNA-mediated loss of MCM7. *n* = 97 cilia. *P* < 0.0001, unpaired *t*-test with Welch's correction. (**D**) qPCR to show MCM7 depletion from human fibroblasts upon siRNA and plasmid nucleofection. *P* = 0.0021, One way ANOVA with Holm-Sidak's multiple comparison test. *n* = 3. (**E**) qPCR to show Mcm7 expression upon nucleofection with a plasmid encoding for zebrafish Mcm7 (concomitant with MCM7 siRNA). *P* = 0.0417, One way ANOVA with Sidak's multiple comparison test. *n* = 3. (**F**) Primary cilia form at similar rates upon nucleofection with CTRL siRNA, MCM7 siRNA or MCM7 siRNA plus a plasmid encoding zebrafish Mcm7. *P* = 0.0799 (siCTRL+empty vector vs siMCM7+empty vector as well as siMCM7+empty vector vs siMCM7+zfMcm7), *P* = 0.0061 (siCTRL+empty vector vs siMCM7+zfMcm7), One-way ANOVA with Holm–Sidak's multiple comparison test. *n* = 3 transfections. One hundred cells were counted in each condition per transfection. (**G**) Primary cilia are shorter in the absence of MCM7. This can be rescued by zebrafish Mcm7 co-nucleofection. ****P < 0.0001, Kruskal–Wallis test with Dunn's multiple comparisons test. *n* = 92 (siCTRL+empty vector), 93 (siMCM7+empty vector) and 95 cilia (siMCM7+zfMcm7) cilia from three nucleofections.

The fact that RNA encoding for human MCM7 rescued the phenotype of Mcm7 MO injected zebrafish embryos suggested conservation of function between the low vertebrate zebrafish and humans. Hence, we performed siRNA-mediated knockdown experiments in 1BR3 hTert-immortalized fibroblasts using a smartpool of siRNAs and induced cilium formation after 3 days of serum starvation. Confocal analysis of immunostained cells indicated the same frequency of ciliation but a reduced length upon loss of MCM7 (Figure 10A–C). To test whether this was specific we repeated these experiments using nucleofection of siRNAs along with either empty vector or a plasmid encoding zebrafish MCM7. Although we did not achieve the same extent of knockdown using nucleofection we saw a robust reduction of human *MCM7* (Figure 10D, Supplementary Figure S9A). Co-nucleofection of zebrafish Mcm7 resulted in varying, but consistent expression of *mcm7* (Figure 10E). Interestingly, we observed a reduction of ciliated cells except under rescue conditions. Nevertheless, expression of zebrafish Mcm7 was able to restore cilium length upon siRNA-mediated downregulation of MCM7 (Figure 10F and G) suggesting that MCM7 is involved in the regulation of ciliogenesis in a conserved manner.

### Loss of MCM7 results in transcriptional changes distinct to MCM2

**Figure 11.**
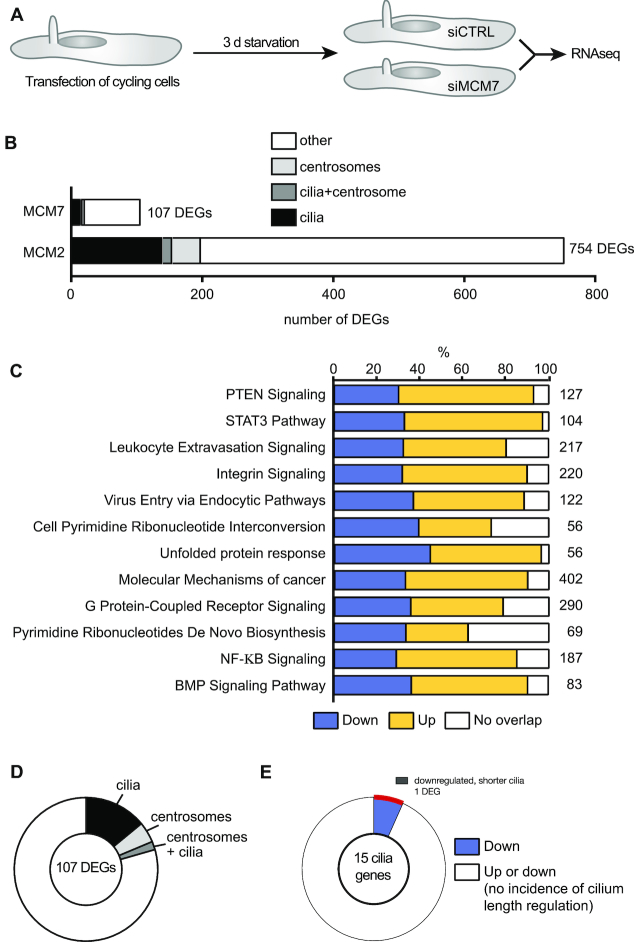
Knockdown of MCM7 in human fibroblasts does not mimic transcriptional changes upon MCM2 knockdown. (**A**) Cycling hTert fibroblasts were transfected with siRNAs and ciliation was induced by serum starvation. Afterwards RNAseq was performed. *n* = 3. (**B)** Bar graph displaying the number of DEGs upon MCM7 knockdown versus MCM2 knockdown in human fibroblasts. DEGs were classified as linked to cilia or centrosomes as described in the methods section. (**C**) Pathways changed upon MCM7 knockdown (cut off: ≥1.5-fold up or down). (**D**) Pie chart showing the percentage of DEGs associated with cilia, centrosomes or both structures according to literature. Only genes with ≥1.5-fold regulation and *P* < 0.05 were considered. (**E**) Only 1 of the 15 DEGs linked to cilia has been reported to alter cilium length (shorter cilia upon knockdown).

These findings suggested that MCM7 might act in concert with MCM2 during ciliogenesis and prompted the prediction that MCM2 and 7 together trigger specific gene expression for faithful ciliogenesis. RNAseq of cells, which were siRNA-transfected during interphase and analysed after three days of starvation (Figure 11A), however, resulted in a smaller number of DEGs than MCM2 knockdown (Figure 11B). Moreover, pathway analysis revealed deregulation of a different set of signalling pathways and processes in MCM7 knockdown cells compared to MCM2 knockdown cells (Figure 11C, compare to Figure 5B). We also assessed which DEGs were related to cilia or centrosomes based on literature and database searches. Approximately one quarter of the 107 DEGs were classified as cilia or centrosome-related genes (Figure 11D and Supplementary Tables S6–S8), however, only a single downregulated DEG has been reported to regulate cilium length (Figure 11E). Moreover, the list of DEGs linked to cilia upon MCM7 depletion showed only minimal overlap with the DEGs of the MCM2 RNAseq. These results suggest that although Mcm7 depletion confers a similar cilia dysfunction phenotype to Mcm2 depletion, its impact on gene expression appears very distinct.

## DISCUSSION

Here, we found that depletion of MCM2 impairs cilia and hence left-right asymmetry development in zebrafish and causes centrosome aberrations as well as shorter cilia in human fibroblasts. Notably, we provide evidence to dissociate this phenotype from the well characterised role of MCM2 during replication since we observed shorter cilia and centrosome abnormalities in fibroblasts when cells have ceased to replicate prior to knockdown. This represents a non-canonical function for MCM2 with direct implications for potential roles in embryonic development and adult physiology. A similar phenotype was also observed following depletion of MCM7. The finding that two components of the MCM complex, which co-ordinate to function as a helicase, share this phenotype suggests that cilia development possibly requires the several components or even the whole MCM complex.

To gain mechanistic insight, we examined the impact of MCM2 or 7 loss on transcription. MCM2 has been reported to bind RNA polymerase II and associate with the transcription machinery (52,55). In addition, transcriptional events are controlled by histone modifications, which can be symmetrically inherited during cell division (67). MCM2 not only associates with histones (52), it also governs equal distribution of modified histones during sister chromatid segregation (68) and helps to maintain silent chromatin during chromosome duplication (69), providing further mechanisms by which MCM2 might influence gene expression. In fact, we found that loss of MCM2 conferred an increase in transcription of a subset of cilia and/or centrosome regulating genes. Importantly, as for the cilia abnormality, this phenotype is also observed after MCM2 knockdown in non-cycling cells. We found MCM2 at transcription start sites of genes upregulated upon MCM2 knockdown and achieved rescue through concomitant inhibition of transcription. Collectively, these findings provide support for a model in which MCM2 has a role in regulating the transcription of genes regulating ciliogenesis or centrosome/centrioles. Surprisingly, however, MCM7 knockdown, whilst conferring a similar phenotype for cilia formation and signalling, had a less marked impact on transcription with fewer DEGs and no overt impact on genes regulating ciliogenesis or centrosomes. Nevertheless, MCM7 was found at the promoter regions of genes upregulated upon MCM2 knockdown.

This similarity in the cilia dysfunction phenotype caused by depletion of MCM2 and 7 but distinction in specific gene regulation could be interpreted in several ways. Firstly, since the impact of MCM2 on genes regulating cilia/centrosome number is striking and observed in distinct assays, it is possible that loss of MCM7 function confers a more subtle impact on transcription, which is indeed observed, and that the specific transcript(s) affecting cilia/centrosome regulation have not yet been identified. Thus as a working model we suggest that the MCM complex impacts on the activity of the transcription machinery of genes controlling centrosome number and cilium elongation. However, it is also possible that there are MCM sub-complexes that have related but distinct functions during transcription, cilia formation and replication. Even during replication, two subsets of MCMs exist, with MCM4–6 serving as the actual helical component and the other MCMs as regulatory measures of the helicase activity (66). Analysis of additional MCM components as well as simultaneous knockdown of two or more MCMs could hence provide interesting insights into the biology of MCMs, not least as it has long been hypothesized that MCMs possess non-canonical (10) and potentially individual functions (11,12). Finally, it is possible that the transcriptional changes are not causal for the cilia dysfunction phenotype. We further note that defects in other components of the replication machinery, such as ATR and ORC1, also confer cilia dysfunction (70,71). Strikingly, moreover, knockdown of ATR in resting cells also triggered similar cilia defects to those observed in this study (71). This, together with our own data provides strong evidence to dissociate the cilia aberration from S phase replication. Whilst this does not eliminate any model involving a transcriptional regulation, it perhaps strengthens the possibility for a potential model involving some aspect of replication such as DNA repair synthesis in resting cells. Thus, although there are several potential mechanisms underlying the ciliogenesis defects in cells deficient in MCM2 and 7, we can conclude that despite the tremendous abundance of MCMs a delicate and potentially spatial control of MCM proteins is likely required for normal development and maintenance of organs. It is furthermore important to point out that some of the deviations from normal embryonic development (i.e. smaller heads), which we used as readouts in our zebrafish experiments could in principle also arise from replication defects and subsequent genomic instability. Mice with reduced MCM2 levels, for instance harbour small genetic deletions as well as progenitor and stem cell deficiencies (72,73), which in turn could contribute to the MCM2 phenotype in zebrafish.

The establishment of internal asymmetry is prerequisite for oriented organ development and placement. It is controlled by cilia in a temporal organizer of laterality (4,13,74,75). Many other processes during development as well as in the adult body depend on cilia. Ciliopathies range from congenital heart defects, asplenia, microcephaly and male infertility to recurrent respiratory infections, retinal degeneration, obesity and even neurological afflictions such as autism (6). Understanding how cilia are built has thus become a forefront matter in molecular medicine. Interestingly, proteins guiding DNA damage recognition and repair have been repeatedly implicated in centrosome and cilia biology (33,54). Initial hypotheses in the 1960s suggested that centrosomes contain DNA. Current studies have shown, however, that centrosomes are solely protein-based organelles and do not contain DNA (76), which raises the question how such nuclear proteins could control centrosomes and cilia. One possibility could be an extra-nuclear function of the proteins and several studies have linked replication proteins such as ORCs or MCMs spatially to the centrosome, and in some cases such proteins exert an impact on centrosomal integrity (46–48,77,78). Here, for MCM2 at least, we propose as a working model that it has a function in transcriptional regulation of a subset of genes including those functioning in cilia/centrosome biology, providing a potential alternative explanation for the unexpected function in cilia development.

MCM2 represents an essential component of the MCM2–7 complex that is loaded onto the DNA double strand to license origins of replication and unwinds the DNA double strand to allow for duplication (8). Not surprisingly, it is used as a marker for proliferating cells (38). Interestingly though, MCM2 as well as MCM7 remain expressed, albeit at low levels, in tissues that undergo differentiation rather than genome duplication and proliferation (39) as evident in the tailbud of zebrafish embryos and in G0 human fibroblasts. MCM complexes are furthermore incredibly abundant and outnumber origins of replication. Therefore, additional functions have been proposed such as that reported for MCM4 (11,12). One alternative function of MCM2 may be the activation of dormant origins under conditions of replication stress (79) suggesting the sole purpose of MCMs, including MCM2, consists of facilitating genome duplication and maintenance (80,81). A recent paper aiming to identify mammalian origins reported that MCM7, and potentially other MCM proteins, reside at and upstream of transcription start sites of firing and dormant origins, arguing against a role for MCMs making the choice of which origins should fire (61). However, the presence of loaded MCMs upstream of promoter and transcription start sites, could serve additional purposes without an essential link to replication.

It is also unclear whether binding of the origin licensing complex precedes MCM2 and 7 binding in G0 cells. Other studies have shown co-localisation of transcription start sites and origins of replication (82,83). This might be significant since we have observed overlapping impacts on cilia formation following depletion of ORC1 in zebrafish and in Meier–Gorlin syndrome patients with mutations in ORC1 (70,84). Further work is required to determine if MCM2 binding is related to origin licensing or origin usage at all, and whether loss of ORC1 causes a similar impact on transcription. A model of this nature is possible since MCM2 deficiency particularly affects replication of genomic regions, which are enriched in expressed genes (80).

Intrigued by the transcriptional changes upon MCM2 knockdown in cells we mined the upstream sequences of the genes regulated by MCM2 knockdown for enriched sequences. This revealed a 7 nucleotide element, highly homologous to the DNA binding motif of KAISO (also known as ZBTB33). KAISO can repress transcription in a DNA methylation dependent manner (85). One speculative possibility is that KAISO can recruit MCM2 to transcription start sites of certain genes. Significantly, Kaiso knockdown causes microcephaly in zebrafish (86) and premature transcription activation upon knockdown in Xenopus (86,87). Loss of KAISO has further been associated with upregulation of Cyclin B2, which we see in our MCM2 RNAseq (87). KAISO, however, was not identified in a recent MCM interaction study although other interactions with the transcription machinery were observed (55). Some interactions in this study were exclusive for MCM2, which included some centrosome (Ninein, CEP89) or cilia (Rotatin, DYNC1H1 and DNAH8) proteins (55). Although these interactions cannot explain why loss of MCM7 did not result in the same transcriptional changes despite producing similar cilia phenotypes, they may hint towards an extra-nuclear function of MCM2, potentially directly at the cilium.

Another point to consider is that in principle all members of the MCM complex have AAA+ ATPase domains (88) and it remains to be tested whether this feature is required or dispensable for ciliogenesis. Protein modification could present a way to modulate and hence activate MCMs. MCMs undergo multiple posttranslational modifications including phosphorylation, *O*-GlcNAcylation, sumoylation as well as ubiquitination, which exert regulatory activity on MCMs in the course of replication initiation and efficiency (89–96). To which extent such modifications also influence ciliogenesis or transcription control can only be speculated at this point, but remains an interesting avenue to pursue in the future.

In summary, we provide evidence for a novel role of MCM2 and MCM7 in cilia formation and an unanticipated impact of MCM2 on gene expression. Although we cannot fully exclude other modes of action at this stage, we propose that MCM2 functions as a gate-keeper of transcription in quiescent cells. MCM2 occupies transcription start sites of cilia genes in G0 cells, which may present a sterical hindrance for the transcription machinery. It is known that MCM complex proteins interact with RNA polymerase II and many other proteins involved in transcription or RNA processing (52,55). Thus, as a consequence of MCM2 deficiency, RNA polymerase II experiences no obstacle resulting in sustained or upregulated transcription of certain genes, including those that negatively influence cilia length. With our data we hence expand the mechanistic repertoire of vertebrate MCM2. We find a similar function for MCM7 in cilia formation but a milder and distinct impact on gene expression. Further studies will be needed to clarify the discrepancy in gene regulation and the extent of cooperativity between MCM2 and MCM7 loss-of-function conditions and whether the two novel functions for MCM2 are causally related.

## DATA AVAILABILITY

All data are provided in full in the results section of this paper or in the supplementary material except for sequencing data deposited at NCBI-GEO for RNAseq (accession no. GSE120768) and NCBI-SRA for ATACseq (accession no. PRJNA494819).

## Supplementary Material

Supplementary Data
