# Supplementary material for: Resting cells rely on the DNA helicase component MCM2 to build cilia
Source: Nucleic Acids Res. 2018 Oct 17;47(1):134–51. doi: 10.1093/nar/gky945 (PMC6326816; doi:10.1093/nar/gky945)
Supplement: Supplementary Data [file gky945_supplemental_files.pdf]

Supplemental material

Figure S1: Expression of *mcm2* during zebrafish development.
Representative images of in situ hybridization against *mcm2* at different stages are shown.
A, Ubiquitous expression at shield stage. Dorsal to the right.
B, Enrichment of *mcm2* transcripts in the posterior region of the embryo (arrow, tailbud stage).
C, Strong expression of *mcm2* at 8 ss. Arrow indicates tailbud region.
D-F, *mcm2* is enriched in ciliated tissues such as the neural tube (arrow), pronephric duct, eyes (arrow in **E**) and the ear (arrow in **F**)
G, **H**, Expression of *mcm2* at 48 hpf (**G**, lateral view; **H**, top view)

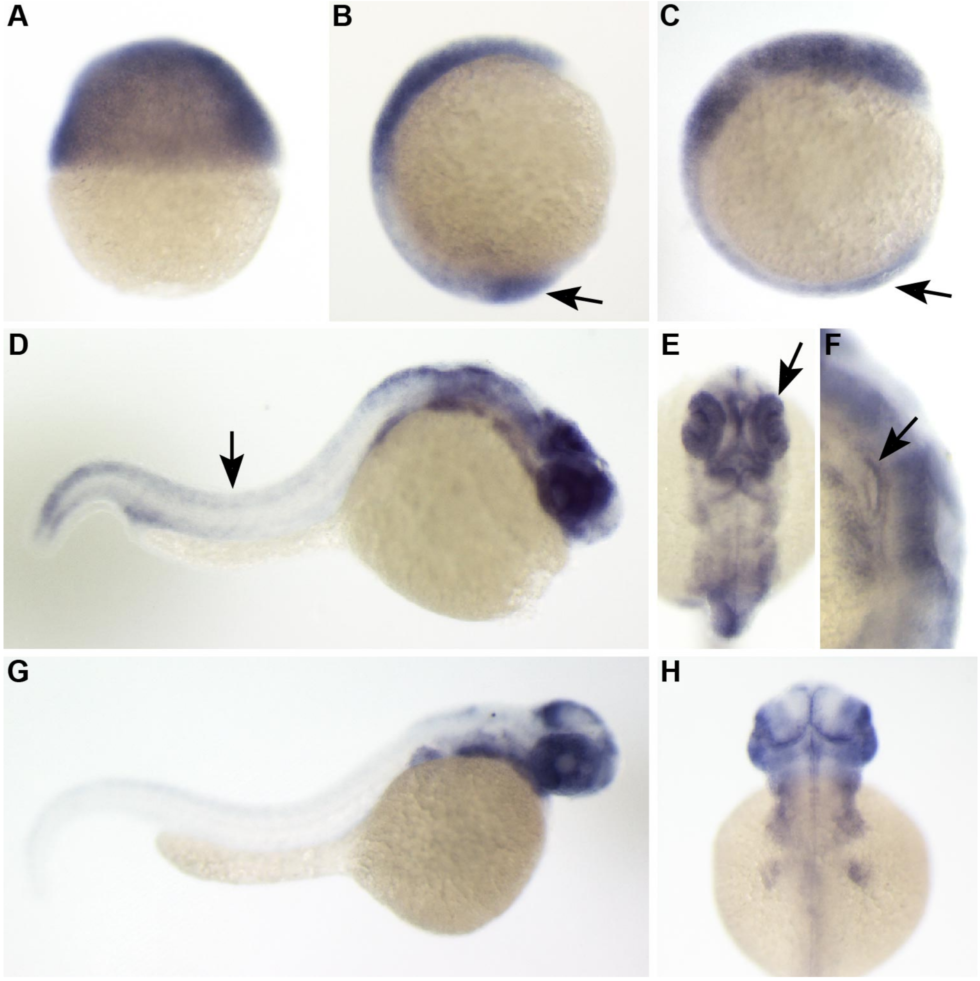

Figure S2: Depletion of MCM2 in human Tert-immortalized 1BR3 fibroblasts.
A, qPCR to show knockdown of *Mcm2* in cells transfected with siRNAs against MCM2. Liposome-based transfection. $p<0.0001$, paired, one-tailed t-test. n=4 experiments.
B, Western blot demonstrating efficient knockdown of MCM2 also on protein level. Representative blots of one of 3 individual transfections.
C, Flow cytometry plots and quantification of cell populations in distinct stages of the cell cycle in non-starved cells. n=4 experiments. ***p=0.0005 (S) and 0.0007 (G2/M), Two-way ANOVA with Sidaks' correction.
D, Flow cytometry plots and quantification of cell populations in distinct stages of the cell cycle in cells synchronized by three days of serum starvation. n=3 experiments. ***p=0.0001 (G1) and 0.0003 (G2/M), Two-way ANOVA with Sidaks' correction.
E, siMCM2 transfected cells form cilia at the same rate as control-transfected cells. p=0.8613, unpaired, two-tailed t-test with Welch's correction. Number of cells: siCTRL=887 cells/17 fields; siMCM2=842 cells/18 fields (n=3 experiments).
F, Primary cilia of MCM2 knockdown cells are shorter. p=0.0005, paired, one-tailed t-test with Welch's correction. 3 experiments. n=41 cilia (siCTRL) and 40 cilia (siMCM2).
G, Analysis of length distribution shows a single population of cilia with a left-shift of cilia length upon depletion of MCM2. n=3 experiments with 109 (siCTRL) and 112(siMCM2) cilia in total.

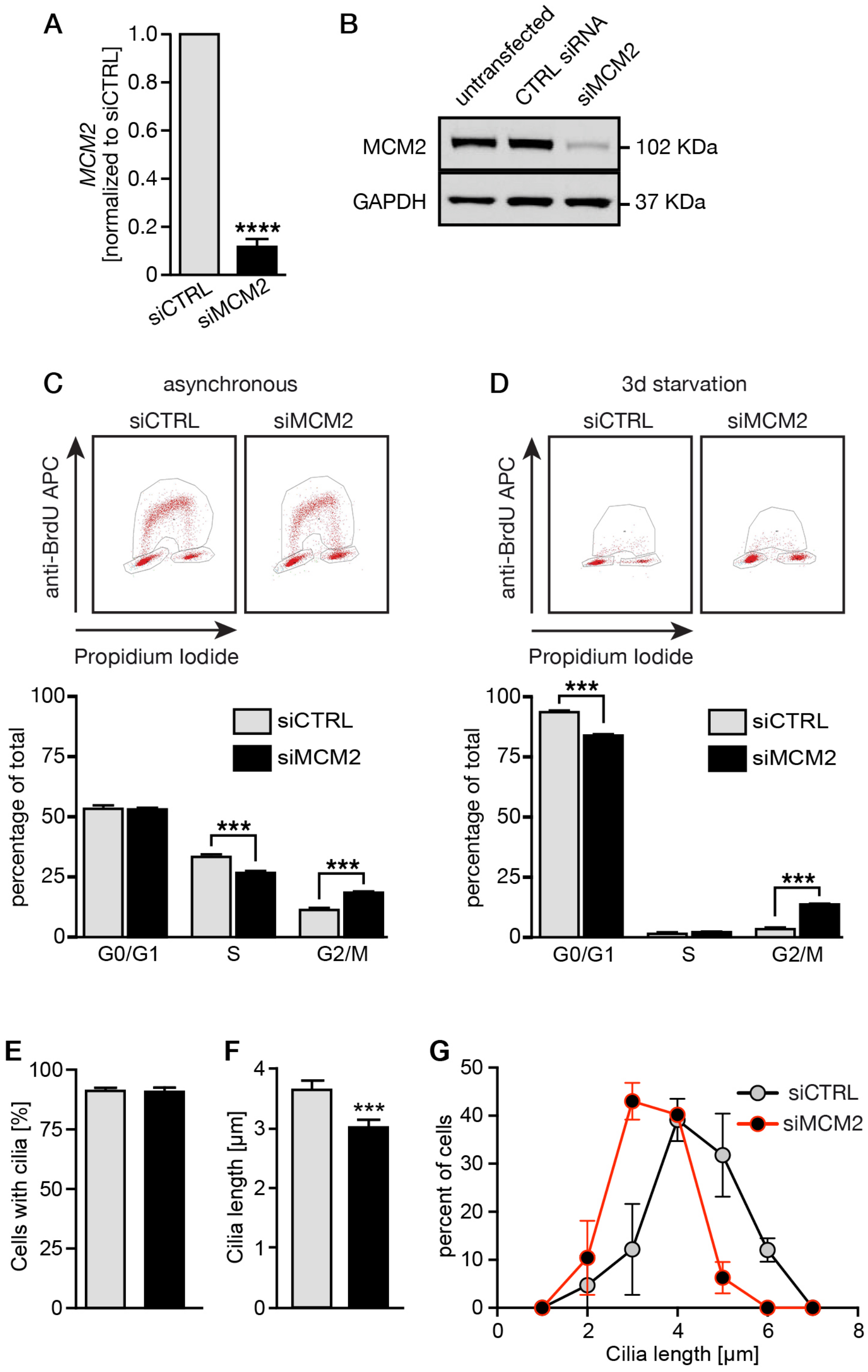

A
MCM2 [normalized to siCTRL]
1.0
0.8
0.6
0.4
0.2
0
****
siCTRL
siMCM2
B
untransfected
CTRL siRNA
siMCM2
MCM2
GAPDH
102 KDa
37 KDa
C
asynchronous
siCTRL
siMCM2
anti-BrdU APC
Propidium Iodide
percentage of total
100
75
50
25
0
***
G0/G1
S
G2/M
D
3d starvation
E
Cells with cilia [%]
F
Cilia length [µm]
G
percent of cells
Cilia length [µm]

Figure S3: Impaired Hedgehog signalling upon Mcm2 knockdown in 1BR3 hTerts.
Cells were transfected with siCTRL and siMCM2. 48 hours post transfection, serum was withdrawn. After another 48 hours cells were stimulated for 24 hours with 200 nM SAG under continuing serum starvation. Hh pathway activity was assessed on the level of target gene expression (*GLI1*). p=0.05, paired, two-tailed Friedman test with Dunn's post-test. n=5 experiments.

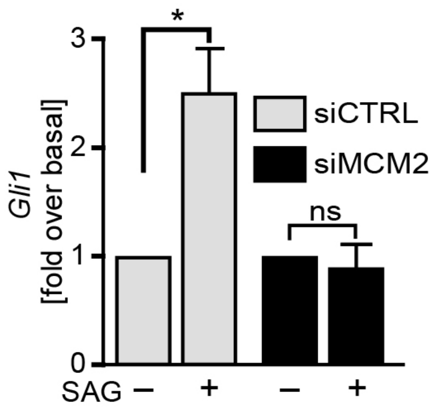

Figure S4: Verification of Mcm2 depletion from human fibroblasts upon nucleofection and expression of a zebrafish rescue construct.

A, qPCR to show MCM2 depletion from human fibroblasts upon siRNA and plasmid nucleofection. p=0.0016, One way ANOVA with Holm-Sidak's multiple comparison test. n=3.

B, qPCR to show Mcm2 expression upon nucleofection with a plasmid encoding for zebrafish Mcm2 (concomitant with human MCM2 siRNA). p=0.0399, One way ANOVA with Sidak's multiple comparison test. n=3.

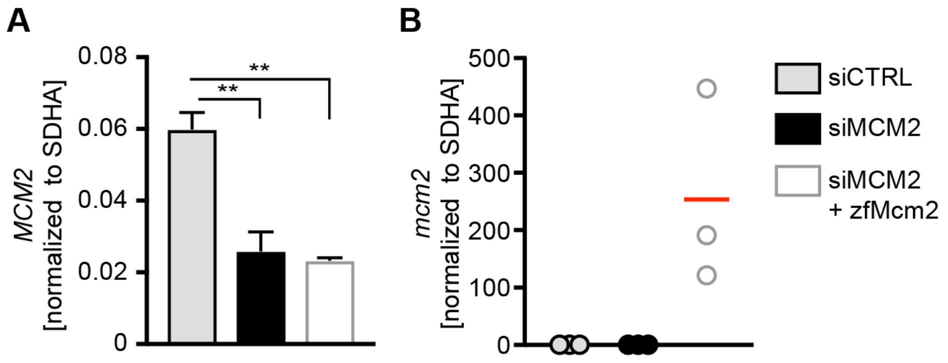

Figure S5:

A, Pericentrin staining (PCNT) reveals excessive pericentriolar material after MCM2 knockdown. Scale bar: 10 µm.

B, Quantification of aberrant PCNT accumulation in control and MCM2 siRNA transfected cycling cells. n=15 fields from three transfections. p<0.0001. Unpaired, two-tailed t-test with Welch's correction.

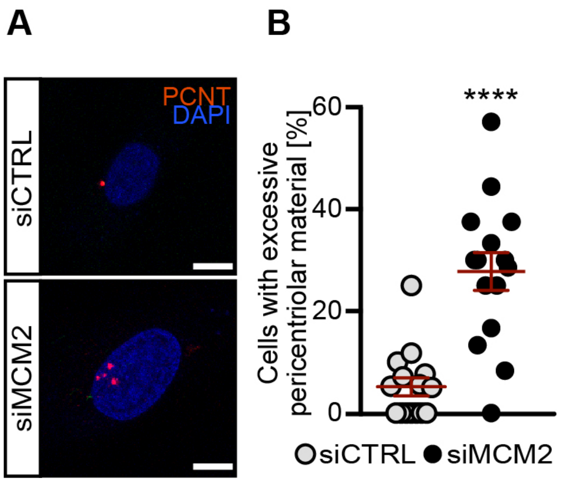

Figure S6: Validation of RNAseq results.

A, qPCR to validate the knockdown of MCM2 in ciliated cells used for RNAseq. p=0.001, paired, two-tailed t-test. n=3 experiments.

B, MA plot showing the overlap of differentially expressed genes (DEG) identified by DESeq and edgeR.

C, Validation of *Aurora kinase A* (*Aurka*) expression after MCM2 knockdown. p=0.0052, paired, two-tailed t-test. n=3 experiments.

D, Validation of *MOK kinase* (*Mok*) expression after MCM2 knockdown. p=0.0197, paired, two-tailed t-test. n=3 experiments.

E, Validation of *Cep152* expression after MCM2 knockdown. p=0.012, paired, two-tailed t-test. n=6 experiments.

F, Validation of *Polo-like kinase 4* (*Plk4*) expression after MCM2 knockdown. p=0.0085, paired, two-tailed t-test. n=3 experiments.

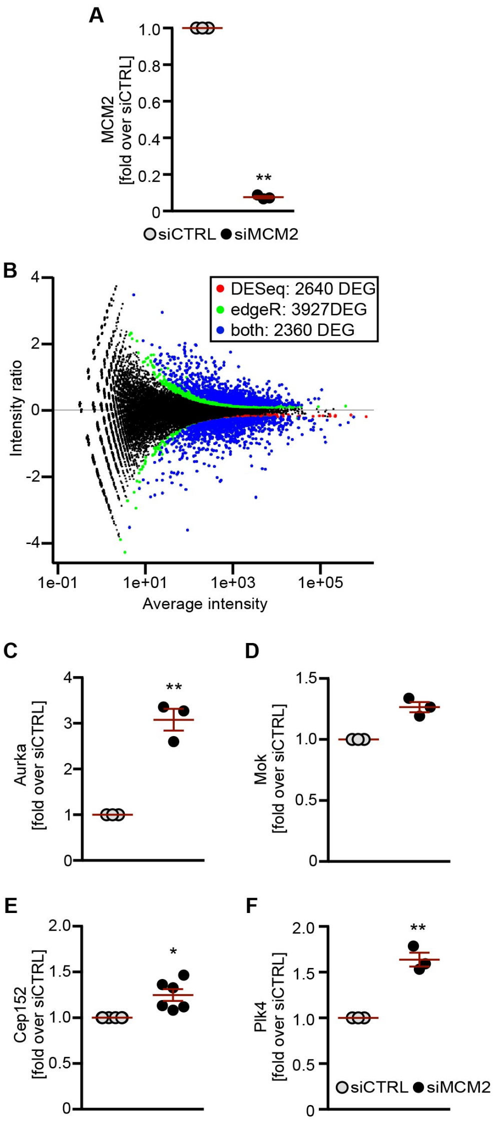

Figure S7: Consensus sequence
Sequence motif enrichment analysis revealed a consensus sequence in promoters of DEGs in MCM2 knockdown cells associated with cilia and centrosomes.

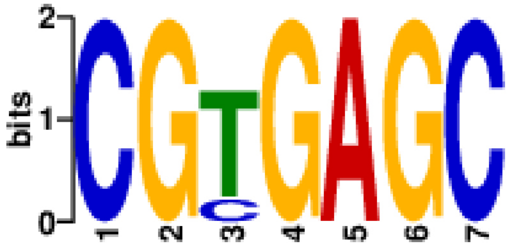

Figure S8: Expression of *mcm7* during zebrafish development.
Representative images of in situ hybridization against *mcm7* at different stages are shown.
A, Ubiquitous expression at shield stage. Dorsal to the right.
B, Enrichment of *mcm7* transcripts in the posterior region of the embryo (arrow, tailbud stage).
C, Strong expression of *mcm7* at 8 ss. Arrow indicates tailbud region.
D-F, *mcm7* is enriched in ciliated tissues such as the neural tube (arrow), pronephric duct, eyes (arrow in **E**) and the ear (arrow in **F**)
G, **H**, Expression of *mcm7* at 48 hpf (**G**, lateral view; **H**, top view)

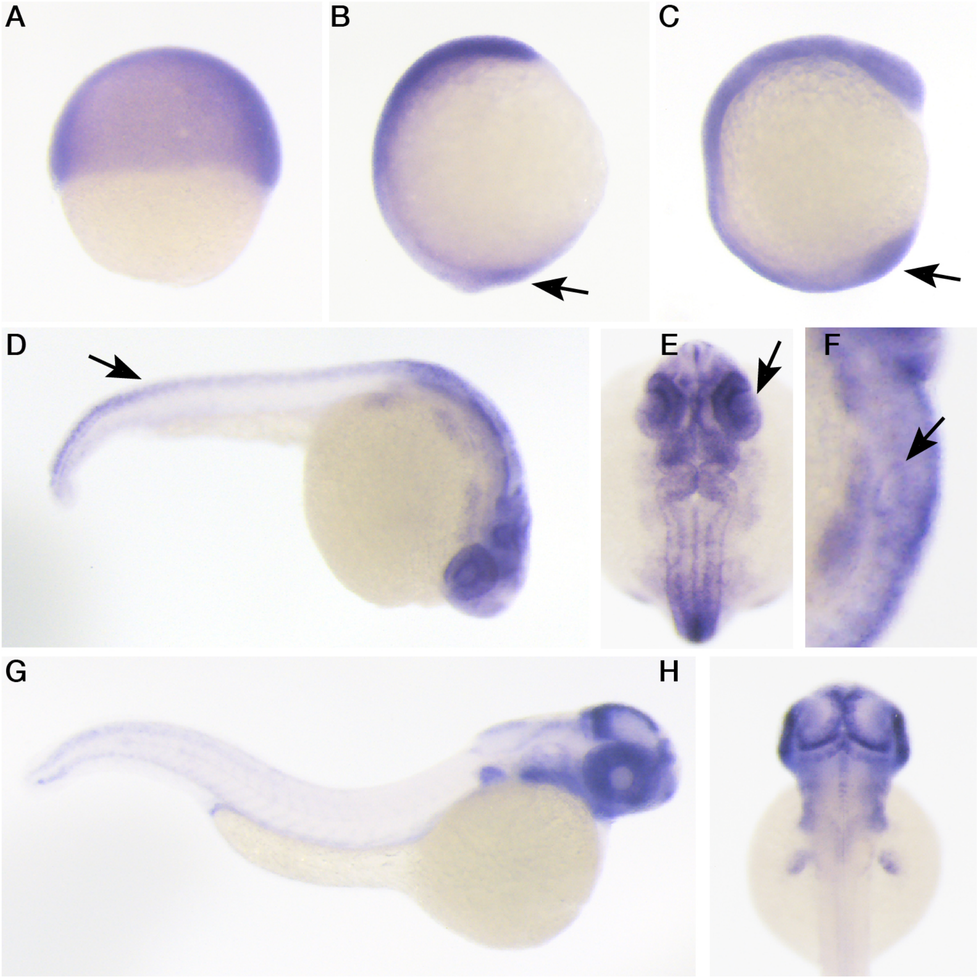

Figure S9: Validation of MCM7 RNAseq results.
A, qPCR showing the knockdown efficiency upon liposome-based transfection of siMCM7 compared to siCTRL. p=0.0082, paired, two-tailed t-test. n=3 experiments.
B, Verification of PTGDS regulation. p=0.0444, paired, two-tailed t-test. n=5 experiments.
C, MCM7 knockdown reduces *NEK7* levels. p=0.0358, paired, two-tailed t-test. n=4 experiments.
D, *RAB39B* reduction upon MCM7 knockdown can be verified. p=0.0054, paired, two-tailed t-test. n=4 experiments.

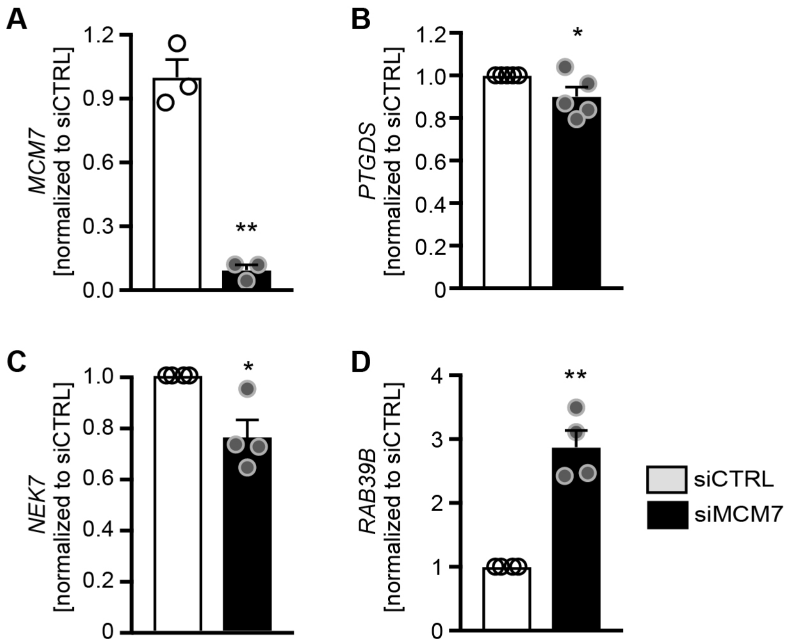

*

Table S1: Primers and probes used in this study

Gene	Species	UP	left primer	right primer
**Assays to assess gene expression**				
AURKA	homo sapiens	61	cgccctgtaggatactgctt	caaatatccccgcactctg
beta2-microglobulin	danio rerio	65	acatcactgtacaggggaaagtc	tccgttcttcagcagttcaa
CAST	homo sapiens	31	gcagtcaatcctccagaacc	caccagaggaagctgacacc
CDC25C	homo sapiens	65	tgggcaaatttcttggtgat	aagatcgaggcaacgttttg
CEP97	homo sapiens	34	agaagattttgagcaaacagaggt	ttcattttggctttggttca
CEP152	homo sapiens	2	gaccactgaaaaggagcaaca	ccttttcaagctgttgaatgagt
GLI1	homo sapiens	7	ccagccagagagaccaacag	cccgcttcttggtcaactt
gli1	danio rerio	5	ggtctcgatgccagtgga	cactgacggagccagtcc
IFT74	homo sapiens	12	tgggcctcagaaagaagttaag	atttgtgattgctggccatt
KLRG1	homo sapiens	10	aacggacaatcaggaaatgag	ccttgagaagtttagaggtgatcc
MACF1	homo sapiens	21	cctttgtgatggttctgcaa	tgttgtttctgcagggtcttt
MCM2	homo sapiens	27	gccaagatgtacagtgacctga	gatgtgccgcaccgtaat
MOK	homo sapiens	51	ttcacagagatgtaaaaccagaaaa	cggctgcttggaatagacac
NEK2	homo sapiens	69	cattggcacaggctcctac	tggagccatagtcaagttctttc
nkx2.2a	danio rerio	89	actagatggctcgcaaccac	cttgagagttcgcggacag
PLK1	homo sapiens	30	cacagtgtcaatgcctcca	ttgctgacccagaagatgg
PLK4	homo sapiens	82	gaaaaccaaaaaggctgtggt	tgagatgcatactcctttacaagc
POC1A	homo sapiens	42	agcacgctggagcacatt	gctgctccagaatggagact
PRC1	homo sapiens	50	tttacaaaccgaggaggaaatc	tcgtgccttcaactcttcttc
PTGS2	homo sapiens	23	cttcacgcatcagtttttcaag	tcaccgtaaatatgatttaagtccac
RTTN	homo sapiens	39	tcaggatcgagatccaagttc	cagtactccccaatgaaggtg
SDHA	homo sapiens	80	ggacctggttgtctttggtc	ccagcgtttggtttaattgg
STIL	homo sapiens	57	catggagcctatatatccttttgc	catcctgcttgaaggaaacc
SYNE1	homo sapiens	64	gtggcacctgacctcctg	acggtcttcaaaaccagcaa
TBXAS1	homo sapiens	30	ggagaccttcaaccctgaaa	aagggcaggtacgtgaagg
UB2C	homo sapiens	8	catgatgtctggcgataaagg	cgagagcttatacctcaggtcttc
**Assays against promotor regions**				
AURKA	homo sapiens	22	tgggactgccacaggtct	tgggactgccacaggtct
BCL-2	homo sapiens	27	tttcaatggaaacctttgagatt	caagtaacacggctaaaaagaatg
CDC25C	homo sapiens	64	cgtcggtgaattcaggttcta	acactggaagaggggcaac
UB2C	homo sapiens	10	gtcgagggcggagacttc	gtcccccatctctcgaatc
**ATAC-seq primers**				
Ad1-noMX	AAT GAT ACG GCG ACC ACC GAG ATC TAC ACT CGT CGG CAG CGT CAG ATG TG			
Ad2.1_TAAGGCGA	CAA GCA GAA GAC GGC ATA CGA GAT TCG CCT TAG TCT CGT GGG CTC GGA GAT GT			
Ad2.2_CGTACTAG	CAA GCA GAA GAC GGC ATA CGA GAT CTA GTA CGG TCT CGT GGG CTC GGA GAT GT			

Ad2.3_AGGCAGAA	CAA GCA GAA GAC GGC ATA CGA GAT TTC TGC CTG TCT CGT GGG CTC GGA GAT GT
Ad2.4_TCCTGAGC	CAA GCA GAA GAC GGC ATA CGA GAT GCT CAG GAG TCT CGT GGG CTC GGA GAT GT
Ad2.5_GGACTCCT	CAA GCA GAA GAC GGC ATA CGA GAT AGG AGT CCG TCT CGT GGG CTC GGA GAT GT
Ad2.6_TAGGCATG	CAA GCA GAA GAC GGC ATA CGA GAT CAT GCC TAG TCT CGT GGG CTC GGA GAT GT

Sequences are given from 5’ to 3’. UP, Universal probe.

Table S2
Differentially expressed genes (DEGs) after MCM2 KD connected to cilia

Ensembl Gene ID	Gene Name	Fold change	Link to cilia	Reference
ENSG00000196616	ADH1B	0.169744523	in Paramecium cilia proteome	(1)
ENSG00000187758	ADH1A	0.199706154	in Paramecium cilia proteome	(1)
ENSG00000087111	PIGS	0.243030101	in mouse cilia proteome	(2)
ENSG00000196344	ADH7	0.245819495	in Paramecium cilia proteome	(1)
ENSG00000248144	ADH1C	0.27389239	in Paramecium cilia proteome	(1)
ENSG00000172020	GAP43	0.282782581	in proteome of rat olfactory cilia	(3)
ENSG00000114115	RBP1	0.318766364	in mouse cilia proteome	(2)
ENSG00000120162	MOB3B	0.380273768	enriched in ciliated cells	(4)
ENSG00000073756	PTGS2	0.382297887	fewer kinocilia in PTGS2 morphant zebrafish	(5)
ENSG00000241553	ARPC4	0.389752864	in mouse cilia proteome	(2)
ENSG00000133083	DCLK1	0.400836314	in mouse cilia proteome	(2)
ENSG00000082458	DLG3	0.4051885	more cilia in node of DLG3 genetrap mice	(6)
ENSG00000103710	RASL12	0.427391311	enriched in ciliated cells	(4)
ENSG00000198959	TGM2	0.432658817	in mouse cilia proteome	(2)
ENSG00000066629	EML1	0.439793959	fewer cilia in EML1 KO mice	(7)
ENSG00000005471	ABCB4	0.441831643	x-box containing genes in C elegans	(8,9)
ENSG00000107882	SUFU	0.452085885	localizes to cilia	(10,11)
ENSG00000042317	SPATA7	0.457689568	recruits other proteins to cilia	(12)
ENSG00000058404	CAMK2B	0.464112881	in flagellum proteome of C. reinhardtii, x-box containing genes in C elegans	(13)
ENSG00000119782	FKBP1B	0.476961389	in flagellum proteome of C. reinhardtii, x-box containing genes in C elegans	(13)
ENSG00000102935	ZNF423	0.484575284	mutated in ciliopathy	(14)
ENSG00000107317	PTGDS	0.501952534	localizes to cilia	(15)
ENSG00000152953	STK32B	0.504274749	enriched in ciliated cells	(4)
ENSG00000135472	FAIM2	0.509828503	in cilia proteome	(16)
ENSG00000131016	AKAP12	0.517180209	in mouse cilia proteome	(2)
ENSG00000087116	ADAMTS2	0.51726918	enriched in ciliated cells	(4)
ENSG00000196776	CD47	0.527025477	in mouse cilia proteome	(2)
ENSG00000143819	EPHX1	0.533215674	in proteome of rat olfactory cilia	(3)
ENSG00000060982	BCAT1	0.53522729	in mouse cilia proteome	(2)
ENSG00000153395	LPCAT1	0.544257035	in mouse cilia proteome	(2)
ENSG00000251258	RFPL4B	0.557650735	x-box containing genes in C elegans	(8)
ENSG00000146676	PURB	0.558654659	in mouse cilia proteome	(2)
ENSG00000183508	FAM46C	0.562562218	enriched in ciliated cells	(4)
ENSG00000133110	POSTN	0.566031854	in flagellum proteome of C. reinhardtii, x-box containing genes in C elegans	(13)
ENSG00000153310	FAM49B	0.570250548	in mouse primary cilia proteome	(17)
ENSG00000198848	CES1	0.573144446	in proteome of rat olfactory cilia	(3)
ENSG00000135636	DYSF	0.579879305	enriched in ciliated cells	(4)

ENSG00000116711	PLA2G4A	0.591474294	localizes to base of cilium and may promote Smo ciliary localization	(18)
ENSG00000006283	CACNA1G	0.598897157	x-box containing genes in C elegans	(8)
ENSG00000182463	TSHZ2	0.603111779	enriched in ciliated cells	(4)
ENSG00000181982	CCDC149	0.607827502	Cilium protein	(19)
ENSG00000129467	ADCY4	0.608601306	localizes to cilia	(20)
ENSG00000100077	ADRBK2	0.609483791	required for odorant receptor signaling	(21)
ENSG00000187134	AKR1C1	0.609671479	basal body proteome Giardia lamblia	(22)
ENSG00000156171	DRAM2	0.612365275	enriched in ciliated cells	(4)
ENSG00000105245	NUMBL	0.612547178	enriched in ciliated cells	(4)
ENSG00000183597	TANGO2	0.619469727	enriched in ciliated cells	(4)
ENSG00000100979	PLTP	0.624600083	in mouse cilia proteome	(2)
ENSG00000100379	KCTD17	0.624887143	fewer and shorter cilia upon knockdown of Kctd17	(23)
ENSG00000108511	HOXB6	0.626687249	enriched in ciliated cells	(4)
ENSG00000149054	ZNF215	0.629519529	enriched in ciliated cells	(4)
ENSG00000168067	MAP4K2	0.630945067	x-box containing genes in C elegans	(8)
ENSG00000160703	NLRX1	0.634936227	localizes to cilia	(24)
ENSG00000134569	LRP4	0.640222649	shorter cilia upon knockdown of LRP4	(25)
ENSG00000134201	GSTM5	0.642123446	in Paramecium cilia proteome	(9)
ENSG00000204084	INPP5B	0.643878109	fewer and shorter cilia in Inpp5b morphant zebrafish	(26)
ENSG00000276600	RAB7B	0.647358203	in flagellum proteome of C. reinhardtii, x-box containing genes in C elegans	(13)
ENSG00000104043	ATP8B4	0.64786358	in Paramecium cilium membrane proteome	(9)
ENSG00000103126	AXIN1	0.652315899	shorter cilia upon overexpression of axin1	(27)
ENSG00000130227	XPO7	0.653542399	in mouse primary cilia proteome	(17)
ENSG00000008300	CELSR3	0.655320535	misoriented and shorter cilia in Celsr3 mutant	(28)
ENSG00000248487	ABHD14A	0.655652478	expressed in ciliated neurons	(29)
ENSG00000250722	SEPP1	0.659785327	affects sperm structure	(30)
ENSG00000124766	SOX4	0.662643638	fewer and shorter cilia in Sox4 KO mice	(31)
ENSG00000174963	ZIC4	0.667256106	likely cilia defect, impaired Hh signaling in Zic4 KO mice	(32)
ENSG00000004139	SARM1	0.6698550	enriched in ciliated cells	(4)
ENSG00000264364	DYNLL2	0.670641861	localizes to cilia	(33)
ENSG00000154258	ABCA9	0.681924484	basal body proteome Giardia lamblia	(22)
ENSG00000169047	IRS1	0.686324141	localizes to ciliary transition zone	(34)
ENSG00000140718	FTO	0.687134826	fewer and shorter cilia in Fto KO mouse	(35)
ENSG00000174705	SH3PXD2B	0.689005982	fewer cilia in SH3PXD2B mutant mouse	(36)
ENSG00000148926	ADM	0.692057587	regulates ciliary beating frequency	(37)
ENSG00000071054	MAP4K4	0.693741161	x-box containing genes in C elegans	(8)
ENSG00000107581	EIF3A	0.693995024	in mouse cilia proteome	(2)
ENSG00000203667	COX20	1.500296763	in mouse cilia proteome	(2)
ENSG00000162616	DNAJB4	1.506497653	basal body proteome of Giardia lamblia	

ENSG00000265972	TXNIP	1.507661812	Chlamydomonas cilia gene	(38)
ENSG00000122861	PLAU	1.510428465	Chlamydomonas cilia gene	(38)
ENSG00000113368	LMNB1	1.513598216	in mouse cilia proteome	(2)
ENSG00000065491	TBC1D22B	1.515190875	enriched in ciliated cells	(4)
ENSG00000052795	FNIP2	1.517145314	enriched in ciliated cells	(4)
ENSG00000196937	FAM3C	1.523564815	in mouse cilia proteome	(2)
ENSG00000164051	CCDC51	1.526591943	enriched in ciliated cells	(4)
ENSG00000101935	AMMECR1	1.527670288	enriched in ciliated cells	(4)
ENSG00000221818	EBF2	1.527680329	enriched in ciliated cells	(4)
ENSG00000108506	INTS2	1.53069183	enriched in ciliated cells	(4)
ENSG00000070087	PFN2	1.531155055	in Paramecium cilia proteome	(9)
ENSG00000197594	ENPP1	1.533159217	in proteome of rat olfactory cilia	(3)
ENSG00000116675	DNAJC6	1.538345345	in mouse cilia proteome	(2)
ENSG00000103642	LACTB	1.541320641	x-box containing genes in C elegans	(8)
ENSG00000107020	PLGRKT	1.56014379	in mouse cilia proteome	(2)
ENSG00000105854	PON2	1.567020748	in mouse cilia proteome	(2)
ENSG00000118503	TNFAIP3	1.568619964	in mouse cilia proteome	(2)
ENSG00000148842	CNNM2	1.584471057	in mouse cilia proteome	(2)
ENSG00000107984	DKK1	1.586292679	shorter cilia upon overexpression of Dkk1	(27)
ENSG00000121898	CPXM2	1.608856339	enriched in ciliated cells	(4)
ENSG00000136867	SLC31A2	1.620576369	longer cilia upon knockdown of Slc31A2	(25)
ENSG00000171848	RRM2	1.630938806	enriched in ciliated cells	(4)
ENSG00000185924	RTN4RL1	1.639085991	enriched in ciliated cells	(4)
ENSG00000161800	RACGAP1	1.66083422	longer cilia upon knockdown of RACGAP1	(25)
ENSG00000049759	NEDD4L	1.662035718	enriched in ciliated cells	(4)
ENSG00000121769	FABP3	1.666438206	in mouse cilia proteome	(2)
ENSG00000182612	TSPAN10	1.677778348	localizes to base of cilium	
ENSG00000090339	ICAM1	1.680537441	longer cilia upon knockdown of Icam1	(25)
ENSG00000138190	EXOC6	1.684537101	enriched in ciliated cells	(4)
ENSG00000131737	KRT34	1.691964477	cilia membrane proteome	(39)
ENSG00000198018	ENTPD7	1.69830505	enriched in ciliated cells	(4)
ENSG00000182481	KPNA2	1.704716204	in cilium proteome	(2,17)
ENSG00000140450	ARRDC4	1.705152245	in Paramecium cilia proteome	(1)
ENSG00000172164	SNTB1	1.708233237	longer cilia upon knockdown of Sntb1	(25)
ENSG00000171097	CCBL1	1.730136994	x-box containing genes in C elegans	(8)
ENSG00000166603	MC4R	1.770363825	localizes to cilia of neurons	(40)
ENSG00000091409	ITGA6	1.782963047	in proteome of rat olfactory cilia	(3)
ENSG00000163738	MTHFD2L	1.79164263	enriched in ciliated cells	(4)
ENSG00000158246	FAM46B	1.795400938	enriched in ciliated cells	(4)
ENSG00000171617	ENC1	1.797297238	more and shorter cilia in Enc1 knockdown zebrafish	(41)
ENSG00000108602	ALDH3A1	1.804668984	enriched in ciliated cells	(4)

ENSG00000169252	ADRB2	1.806024544	regulates ciliary beating frequency	(42)
ENSG00000157542	KCNJ6	1.819188251	chlamydomonas cilia gene	(43)
ENSG00000023445	BIRC3	1.852270653	elongated cilia upon Birc3 knockdown	(44)
ENSG00000169213	RAB3B	1.863110881	enriched in ciliated cells	(4)
ENSG00000106366	SERPINE1	1.914434501	in flagellum proteome of C. reinhardtii	(13)
ENSG00000166562	SEC11C	1.919398348	in mouse cilia proteome	(2)
ENSG00000080823	MOK	1.957920343	longer cilia upon knockdown of Mok	(45)
ENSG00000170540	ARL6IP1	1.967774064	shorter cilia in Arl6IP1 knockdown zebrafish	(46)
ENSG00000106484	MEST	2.067447136	in mouse cilia proteome	(2)
ENSG00000130702	LAMA5	2.17560701	shorter cilia in Lama5 KO mice	(47)
ENSG00000117399	CDC20	2.383269934	longer cilia upon knockdown of CDC20	(48)
ENSG00000175591	P2RY2	2.404177479	regulates ciliary beating	(49)
ENSG00000144959	NCEH1	2.53050724	in mouse cilia proteome	(2)
ENSG00000198901	PRC1	2.669464664	longer cilia upon knockdown of Prc1	(25)
ENSG00000183856	IQGAP3	2.755777894	x-box containing genes in C elegans	(8)
ENSG00000099998	GGT5	2.774407556	longer cilia upon knockdown of Ggt5	(25)
ENSG00000105650	PDE4C	3.009566601	localizes to cilia	(50)
ENSG00000134057	CCNB1	3.143056581	localizes to cilia	(51)
ENSG00000137642	SORL1	3.161819054	in proteome of rat olfactory cilia	(3)
ENSG00000118193	KIF14	3.570943341	Kif14 loss-of-function leads to lethal ciliopathy phenotype	(52)
ENSG00000175063	UBE2C	3.696583571	longer cilia upon knockdown of Ube2c	(25)
ENSG00000162975	KCNF1	5.149065891	localizes to base of cilium, fewer cilia upon Kcnf1 knockdown	(53)
ENSG00000160870	CYP3A7	11.4483856	in Tetrahymena basal body	(54)

Table S3
Differentially expressed genes (DEGs) after MCM2 KD connected to centrosomes

Ensembl Gene ID	Gene Name	Fold change	Link to centrosomes	Reference
ENSG00000185379	RAD51D	0.5163870	centrosome fragmentation in Rad51D KO mouse	(55)
ENSG00000102760	RGCC	0.5445294	localizes to centrosome	(56)
ENSG00000100034	PPM1F	0.5457965	regulates centrosome placement	(57)
ENSG00000071655	MBD3	0.5658023	localizes to centrosome	(58)
ENSG00000184900	SUMO3	0.6907896	localizes to centrosome	(59)
ENSG00000221869	CEBPD	0.6994844	centrosome amplification in Cebpd KO	(60)
ENSG00000003393	ALS2	1.5218121	localizes to centrosome	(61)
ENSG00000111057	KRT18	1.5867981	localizes to centrosome	(62)
ENSG00000135069	PSAT1	1.5975973	in centrosome proteome	(63)
ENSG00000111206	FOXM1	1.6081531	supernumerary centrosomes upon knockdown of Foxm1	(64)

ENSG00000159792	PSKH1	1.6446089	localizes to centrosome	(65)
ENSG00000173848	NET1	1.6548582	required for centrosomal activation of Aurka	(66)
ENSG00000105810	CDK6	1.7213735	supernumerary centrosomes in patient cells	(67)
ENSG00000143476	DTL	1.7449420	localizes to centrosome	(68)
ENSG00000004864	SLC25A13	1.7477434	in Drosophila centrosome proteome	(69)
ENSG00000165304	MELK	1.7895999	localizes to centrosome in C. elegans	(70)
ENSG00000204580	DDR1	1.8178080	prevents centrosome clustering	(71)
ENSG00000113645	WWC1	1.8252957	supernumerary centrosomes upon knockdown of Wwc1	(72)
ENSG00000138160	KIF11	1.9076332	monopolar spindles upon knockdown of Kif11	(73)
ENSG00000100526	CDKN3	1.9255400	promotes centrosome overduplication	(74)
ENSG00000105976	MET	1.9562550	supernumerary centrosomes upon Met overexpression	(75)
ENSG00000145386	CCNA2	1.9598318	involved in centrosome overduplication	(76)
ENSG00000144554	FANCD2	1.9694022	localizes to centrosome	(77)
ENSG00000128849	CGNL1	1.9836346	in centrosome proteome	(63)
ENSG00000138778	CENPE	2.0776933	localizes to centrosome	(78)
ENSG00000013810	TACC3	2.1164052	required for centrosome dependent microtubule assembly and centrosome clustering	(79,80)
ENSG00000171877	FRMD5	2.1391458	localizes to centrosome	(81)
ENSG00000137812	CASC5	2.2465846	localizes to centrosome	(82)
ENSG00000165480	SKA3	2.2624977	localizes to centrosome	(83)
ENSG00000137804	NUSAP1	2.3530935	Supernumerary centrosomes upon knockdown of Nusap1	(84)
ENSG00000075702	WDR62	2.5262299	localizes to centrosome, supernumerary centrosomes upon knockdown in mice	(85,86)
ENSG00000157456	CCNB2	2.5365449	controls centrosome splitting	(87)
ENSG00000169607	CKAP2L	2.5534307	supernumerary centrosomes in patient cells	(88)
ENSG00000177602	GSG2	2.5564458	Centrosome amplification when inhibited	(89)
ENSG00000129810	SGOL1	2.6786466	facilitates centriole cohesion	(90)
ENSG00000154839	SKA1	2.7253427	centriole splitting upon Ska1 knockdown	(91)
ENSG00000135451	TROAP	2.8241274	localizes to mother centriole	(92)
ENSG00000138180	CEP55	2.8478776	localizes to centrosome	(93)
ENSG00000066279	ASPM	2.8884676	localizes to centrosomes	(94)
ENSG00000126787	DLGAP5	3.1654742	attaches to microtubules of the mother centriole	(95)
ENSG00000131747	TOP2A	3.5113799	localizes to centrosome	(96)
ENSG00000143228	NUF2	3.6246671	localizes to centrosome	(97)
ENSG00000158402	CDC25C	3.8619034	localizes to centrosome	(98)
ENSG00000024526	DEPDC1	4.2117959	involved in centrosome organization	(99)

Table S4
Differentially expressed genes (DEGs) after MCM2 KD connected to cilia and centrosomes

Ensembl Gene ID	Gene Name	Fold change	Link to cilia and centrosomes	Reference
ENSG00000103995	CEP152	1.512970833	fewer cilia in Cep152 morphant zebrafish, loss of centrioles upon Cep152 knockdown	(100,101)
ENSG00000112312	GMNN	1.623392901	fewer cilia in Gmnn morphant zebrafish, centrosome amplification upon Gmnn1 knockdown	(102,103)
ENSG00000123473	STIL	1.741783835	fewer cilia in Stil KO mouse, overexpression causes centriole amplification	(104,105)
ENSG00000142731	PLK4	1.859578533	fewer cilia in Plk4 morphant zebrafish, fewer and longer cilia in mice expressing more Plk4, centriole amplification upon overexpression of Plk4	(106-108)
ENSG00000164087	POC1A	1.963795246	fewer and shorter cilia upon Poc1a knockdown, supernumerary centrosomes in patient cells	(109,110)
ENSG00000156970	BUB1B	2.012013319	fewer cilia in patients and Bub1b morphant medaka, centrosome amplification in patient cells	(111,112)
ENSG00000076382	SPAG5	2.100122344	localizes to photoreceptors and centrosomes	(113,114)
ENSG00000166851	PLK1	2.415426911	cilia disassembly factor, longer cilia upon knockdown, promotes mother centriole duplication	(115-117)
ENSG00000237649	KIFC1	2.602250895	longer cilia upon knockdown of Kifc1, clusters amplified centrosomes	(25,118)
ENSG00000087586	AURKA	2.604312363	promotes cilia disassembly, centrosome amplification upon overexpression	(119,120)
ENSG00000117724	CENPF	2.837427333	shorter and fewer cilia in Cenpf morphant zebrafish, localizes to mother centriole	(121)
ENSG00000138180	CEP55	2.847877639	localizes to centrosome, mutated in ciliopathy Meckel-Gruber Syndrome	(93,122)
ENSG00000112742	TTK	3.236084785	fewer cilia upon overexpression, localizes to centrosome	(123,124)
ENSG00000072571	HMMR	4.033096533	fewer cilia upon knockdown of Hmmr, inhibits centrosome amplification	(125,126)
ENSG00000117650	NEK2	4.05741635	shorter cilia upon Nek2 knockdown, centrosome amplification	(127,128)

Table S5

List of genes with more accessible promoter regions in MCM2 knockdown cells according to ATACseq. Only genes associated with cilia or centrosomes are listed.

Assignment of function was based on literature search using PubMed, Genecards and Google.

Chromosome	Amplicon length	Associated gene loci	Fold enrichment	Link to cilia or centrosome	Refernz
1	157	MACF1 (ENSG00000127603)	-4.92284	required for cilia formation	(129)
1	146	PTGS2 (ENSG00000073756), PACERR (ENSG00000273129)	-3.45809	fewer kinocilia in PTGS2 morphant zebrafish	(5)
1	173	DNAH14 (ENSG00000185842)	4.85111	Force generating protein of respiratory cilia.	(130)
3	145	CEP97 (ENSG00000182504)	3.14044	enforced expression of CP110 in quiescent cells suppresses their ability to assemble cilia	(131)
3	146	ATG3 (ENSG00000144848), SLC35A5 (ENSG00000138459)	3.11412	ATG3: fewer and shorter cilia in KO	(132)
5	173	CAST (ENSG00000153113)	2.83037	may be involved in basal body docking	(133)
5	156	CDC25C (ENSG00000158402), FAM53C (ENSG00000120709)	-3.17018	CDC25C localizes to centrosome	(98)
6	152	RP1-313I6.12 (ENSG00000272009), ZNF165 (ENSG00000197279)	3.27889	ZNF165: The encoded protein may play a role in spermatogenesis.	(134)
6	158	SYNE1 (ENSG00000131018), SYNE1-AS1 (ENSG00000234577)	-2.33784	May be required for centrosome migration to the apical cell surface during early ciliogenesis.	(135)
7	185	CCDC146 (ENSG00000135205)	5.59744	centrosome associated	(136)
7	155	HIPK2 (ENSG00000064393), TBXAS1 (ENSG00000059377)	-3.3497	Thromboxane affects ciliary beating	(137)
9	151	PLAA (ENSG00000137055), IFT74 (ENSG00000096872)	3.03464	IFT74 potentially involved in tubulin dynamics of ciliary axoneme	(138)
12	147	M6PR (ENSG00000003056), KLRG1 (ENSG00000139187)	2.35994	KLRG1: increased cilia length upon knockdown	(25)

18	156	RTTN (ENSG00000176225)	2.32917	cilia and microcephaly gene, KO with asymmetry defects	(139-141)
21	241	TSPEAR (ENSG00000175894), KRTAP10-4 (ENSG00000215454), KRTAP10-6 (ENSG00000188155)	-2.33862	TSPEAR SNP associated with daefness	(142)
22	188	WNT7B (ENSG00000188064)	-3.51387	longer cilia in mutant	(143)

Table S6
Differentially expressed genes (DEGs) connected to cilia upon MCM7 knockdown

Ensembl Gene ID	Gene Name	Fold change	Link to cilia	Reference
ENSG00000107937	GTPBP4	0.495826746	x-box containing genes in C elegans	(8)
ENSG00000107317	PTGDS	0.571306286	in cilia	(15)
ENSG00000158435	CNOT11	0.6107966	expressed during ciliogenesis in Paramecium	(144)
ENSG00000168000	BSCL2	0.632890764	enriched in ciliated tissues	(4)
ENSG00000155100	OTUD6B	0.654191877	x-box containing genes in C elegans	(8)
ENSG00000160200	CBS	0.660651399	in flagellum proteome of C. reinhardtii, x-box containing genes in C elegans	(8,13)
ENSG00000178695	KCTD12	0.672808505	ciliary candidate gene	(145)
ENSG00000136888	ATP6V1G1	0.684927748	in flagellum proteome of C. reinhardtii	(13)
ENSG00000197217	ENTPD4	1.556064197	enriched in ciliated tissues	(4)
ENSG00000023445	BIRC3	1.637324028	elongated cilia upon KD	(44)
ENSG00000165323	FAT3	1.673421753	enriched in ciliated tissues	(4)
ENSG00000140450	ARRDC4	1.679089854	ciliary gene in C. reinhardtii	(38)
ENSG00000171724	VAT1L	1.878520038	in ciliary proteome	(2)
ENSG00000155961	RAB39B	2.420638189	in cilium proteome, x-box containing genes in C elegans	(2,8)
ENSG00000184524	CEND1	3.178870548	in ciliary proteome	(2)

Table S7
Differentially expressed genes (DEGs) after MCM7 KD connected to centrosomes

Ensembl Gene ID	Gene Name	Fold change	Link to centrosome	Reference
ENSG00000166508	MCM7	0.102311203	centrosomal localization and integrity	(146)
ENSG00000169750	RAC3	0.618059341	fewer centrioles upon Rac3 knockdown	(44)
ENSG00000141933	TPGS1	0.655104299	localizes to centrosome	(81)
ENSG00000100297	MCM5	0.684036776	involved in centrosome duplication	(147)
ENSG00000204580	DDR1	1.529451677	prevents centrosome clustering	(71)

Table S8
Differentially expressed genes (DEGs) after MCM7 KD connected to cilia and centrosomes

Ensembl Gene ID	Gene Name	Fold change	Function	Reference
ENSG00000151414	NEK7	0.577154404	KD: cilia induction, required for procentriole formation	(148)
ENSG00000072571	HMMR	1.993318119	fewer cilia upon knockdown of Hmmr, inhibits centrosome amplification	(125,126)

Supplementary References

1. Arnaiz, O., Malinowska, A., Klotz, C., Sperling, L., Dadlez, M., Koll, F. and Cohen, J. (2009) Cildb: a knowledgebase for centrosomes and cilia. *Database (Oxford)*, **2009**, bap022.
2. Liu, Q., Tan, G., Levenkova, N., Li, T., Pugh, E.N., Jr., Rux, J.J., Speicher, D.W. and Pierce, E.A. (2007) The proteome of the mouse photoreceptor sensory cilium complex. *Mol Cell Proteomics*, **6**, 1299-1317.
3. Mayer, U., Kuller, A., Daiber, P.C., Neudorf, I., Warnken, U., Schnolzer, M., Frings, S. and Mohrlen, F. (2009) The proteome of rat olfactory sensory cilia. *Proteomics*, **9**, 322-334.
4. Blacque, O.E., Perens, E.A., Boroevich, K.A., Inglis, P.N., Li, C., Warner, A., Khattra, J., Holt, R.A., Ou, G., Mah, A.K. *et al.* (2005) Functional genomics of the cilium, a sensory organelle. *Curr Biol*, **15**, 935-941.
5. Jin, D., Ni, T.T., Sun, J., Wan, H., Amack, J.D., Yu, G., Fleming, J., Chiang, C., Li, W., Papierniak, A. *et al.* (2014) Prostaglandin signalling regulates ciliogenesis by modulating intraflagellar transport. *Nat Cell Biol*, **16**, 841-851.
6. Van Campenhout, C.A., Eitelhuber, A., Gloeckner, C.J., Giallonardo, P., Gegg, M., Oller, H., Grant, S.G., Krappmann, D., Ueffing, M. and Lickert, H. (2011) Dlg3 trafficking and apical tight junction formation is regulated by nedd4 and nedd4-2 e3 ubiquitin ligases. *Dev Cell*, **21**, 479-491.
7. Bizzotto, S., Uzquiano, A., Dingli, F., Ershov, D., Houllier, A., Arras, G., Richards, M., Loew, D., Minc, N., Croquelois, A. *et al.* (2017) Eml1 loss impairs apical progenitor spindle length and soma shape in the developing cerebral cortex. *Sci Rep*, **7**, 17308.
8. Efimenko, E., Bubb, K., Mak, H.Y., Holzman, T., Leroux, M.R., Ruvkun, G., Thomas, J.H. and Swoboda, P. (2005) Analysis of xbx genes in C. elegans. *Development*, **132**, 1923-1934.
9. Yano, J., Rajendran, A., Valentine, M.S., Saha, M., Ballif, B.A. and Van Houten, J.L. (2013) Proteomic analysis of the cilia membrane of Paramecium tetraurelia. *J Proteomics*, **78**, 113-122.
10. Haycraft, C.J., Banizs, B., Aydin-Son, Y., Zhang, Q., Michaud, E.J. and Yoder, B.K. (2005) Gli2 and Gli3 localize to cilia and require the intraflagellar transport protein polaris for processing and function. *PLoS Genet*, **1**, e53.
11. He, M., Subramanian, R., Bangs, F., Omelchenko, T., Liem, K.F., Jr., Kapoor, T.M. and Anderson, K.V. (2014) The kinesin-4 protein Kif7 regulates mammalian Hedgehog signalling by organizing the cilium tip compartment. *Nat Cell Biol*, **16**, 663-672.
12. Eblimit, A., Nguyen, T.M., Chen, Y., Esteve-Rudd, J., Zhong, H., Letteboer, S., Van Reeuwijk, J., Simons, D.L., Ding, Q., Wu, K.M. *et al.* (2015) Spata7 is a retinal ciliopathy gene critical for correct RPGRIP1 localization and protein trafficking in the retina. *Hum Mol Genet*, **24**, 1584-1601.
13. Pazour, G.J., Agrin, N., Leszyk, J. and Witman, G.B. (2005) Proteomic analysis of a eukaryotic cilium. *J Cell Biol*, **170**, 103-113.
14. Chaki, M., Airik, R., Ghosh, A.K., Giles, R.H., Chen, R., Slaats, G.G., Wang, H., Hurd, T.W., Zhou, W., Cluckey, A. *et al.* (2012) Exome capture reveals ZNF423 and CEP164 mutations, linking renal ciliopathies to DNA damage response signaling. *Cell*, **150**, 533-548.

15. Czub, B., Shah, A., Kruczek, P., Alfano, G., Chakarova, C. and Bhattacharya, S. (2015), *Cilia*, Vol. 4, pp. P15.
16. Kuhlmann, K., Tschapek, A., Wiese, H., Eisenacher, M., Meyer, H.E., Hatt, H.H., Oeljeklaus, S. and Warscheid, B. (2014) The membrane proteome of sensory cilia to the depth of olfactory receptors. *Mol Cell Proteomics*, **13**, 1828-1843.
17. Ishikawa, H., Thompson, J., Yates, J.R., 3rd and Marshall, W.F. (2012) Proteomic analysis of mammalian primary cilia. *Curr Biol*, **22**, 414-419.
18. Arensdorf, A.M., Dillard, M.E., Menke, J.M., Frank, M.W., Rock, C.O. and Ogden, S.K. (2017) Sonic Hedgehog Activates Phospholipase A2 to Enhance Smoothened Ciliary Translocation. *Cell Rep*, **19**, 2074-2087.
19. Jensen, V.L., Carter, S., Sanders, A.A., Li, C., Kennedy, J., Timbers, T.A., Cai, J., Scheidel, N., Kennedy, B.N., Morin, R.D. *et al.* (2016) Whole-Organism Developmental Expression Profiling Identifies RAB-28 as a Novel Ciliary GTPase Associated with the BBSome and Intraflagellar Transport. *PLoS Genet*, **12**, e1006469.
20. Wong, S.T., Trinh, K., Hacker, B., Chan, G.C., Lowe, G., Gaggar, A., Xia, Z., Gold, G.H. and Storm, D.R. (2000) Disruption of the type III adenylyl cyclase gene leads to peripheral and behavioral anosmia in transgenic mice. *Neuron*, **27**, 487-497.
21. Peppel, K., Boekhoff, I., McDonald, P., Breer, H., Caron, M.G. and Lefkowitz, R.J. (1997) G protein-coupled receptor kinase 3 (GRK3) gene disruption leads to loss of odorant receptor desensitization. *J Biol Chem*, **272**, 25425-25428.
22. Lauwaet, T., Smith, A.J., Reiner, D.S., Romijn, E.P., Wong, C.C., Davids, B.J., Shah, S.A., Yates, J.R., 3rd and Gillin, F.D. (2011) Mining the Giardia genome and proteome for conserved and unique basal body proteins. *Int J Parasitol*, **41**, 1079-1092.
23. Kasahara, K., Kawakami, Y., Kiyono, T., Yonemura, S., Kawamura, Y., Era, S., Matsuzaki, F., Goshima, N. and Inagaki, M. (2014) Ubiquitin-proteasome system controls ciliogenesis at the initial step of axoneme extension. *Nat Commun*, **5**, 5081.
24. Yang, Q., Sun, G., Cao, Z., Yin, H., Qi, Q., Wang, J., Liu, W., Bai, X., Wang, H. and Li, J. (2016) The expression of NLRX1 in C57BL/6 mice cochlear hair cells: Possible relation to aging- and neomycin-induced deafness. *Neurosci Lett*, **616**, 138-146.
25. Kim, J., Lee, J.E., Heynen-Genel, S., Suyama, E., Ono, K., Lee, K., Ideker, T., Aza-Blanc, P. and Gleeson, J.G. (2010) Functional genomic screen for modulators of ciliogenesis and cilium length. *Nature*, **464**, 1048-1051.
26. Luo, N., Kumar, A., Conwell, M., Weinreb, R.N., Anderson, R. and Sun, Y. (2013) Compensatory Role of Inositol 5-Phosphatase INPP5B to OCRL in Primary Cilia Formation in Oculocerebrorenal Syndrome of Lowe. *PLoS One*, **8**, e66727.
27. Caron, A., Xu, X. and Lin, X. (2012) Wnt/beta-catenin signaling directly regulates Foxj1 expression and ciliogenesis in zebrafish Kupffer's vesicle. *Development*, **139**, 514-524.
28. Tissir, F., Qu, Y., Montcouquiol, M., Zhou, L., Komatsu, K., Shi, D., Fujimori, T., Labeau, J., Tyteca, D., Courtoy, P. *et al.* (2010) Lack of cadherins Celsr2 and Celsr3 impairs ependymal ciliogenesis, leading to fatal hydrocephalus. *Nat Neurosci*, **13**, 700-707.
29. McClintock, T.S., Glasser, C.E., Bose, S.C. and Bergman, D.A. (2008) Tissue expression patterns identify mouse cilia genes. *Physiol Genomics*, **32**, 198-206.
30. Olson, G.E., Winfrey, V.P., Nagdas, S.K., Hill, K.E. and Burk, R.F. (2005) Selenoprotein P is required for mouse sperm development. *Biol Reprod*, **73**, 201-211.

31. Poncy, A., Antoniou, A., Cordi, S., Pierreux, C.E., Jacquemin, P. and Lemaigre, F.P. (2015) Transcription factors SOX4 and SOX9 cooperatively control development of bile ducts. *Dev Biol*, **404**, 136-148.
32. Blank, M.C., Grinberg, I., Aryee, E., Laliberte, C., Chizhikov, V.V., Henkelman, R.M. and Millen, K.J. (2011) Multiple developmental programs are altered by loss of Zic1 and Zic4 to cause Dandy-Walker malformation cerebellar pathogenesis. *Development*, **138**, 1207-1216.
33. Goggolidou, P., Stevens, J.L., Agueci, F., Keynton, J., Wheway, G., Grimes, D.T., Patel, S.H., Hilton, H., Morthorst, S.K., DiPaolo, A. *et al.* (2014) ATMIN is a transcriptional regulator of both lung morphogenesis and ciliogenesis. *Development*, **141**, 3966-3977.
34. Wang, H., Zou, X., Wei, Z., Wu, Y., Li, R., Zeng, R., Chen, Z. and Liao, K. (2015) Hsp90alpha forms a stable complex at the cilium neck for the interaction of signalling molecules in IGF-1 receptor signalling. *J Cell Sci*, **128**, 100-108.
35. Osborn, D.P., Roccasecca, R.M., McMurray, F., Hernandez-Hernandez, V., Mukherjee, S., Barroso, I., Stemple, D., Cox, R., Beales, P.L. and Christou-Savina, S. (2014) Loss of FTO antagonises Wnt signaling and leads to developmental defects associated with ciliopathies. *PLoS One*, **9**, e87662.
36. Yang, B., Tian, C., Zhang, Z.G., Han, F.C., Azem, R., Yu, H., Zheng, Y., Jin, G., Arnold, J.E. and Zheng, Q.Y. (2011) Sh3pxd2b mice are a model for craniofacial dysmorphology and otitis media. *PLoS One*, **6**, e22622.
37. Chiu, P.C., Liao, S., Lam, K.K., Tang, F., Ho, J.C., Ho, P.C., O, W.S., Yao, Y.Q. and Yeung, W.S. (2010) Adrenomedullin regulates sperm motility and oviductal ciliary beat via cyclic adenosine 5'-monophosphate/protein kinase A and nitric oxide. *Endocrinology*, **151**, 3336-3347.
38. Merchant, S.S., Prochnik, S.E., Vallon, O., Harris, E.H., Karpowicz, S.J., Witman, G.B., Terry, A., Salamov, A., Fritz-Laylin, L.K., Marechal-Drouard, L. *et al.* (2007) The Chlamydomonas genome reveals the evolution of key animal and plant functions. *Science*, **318**, 245-250.
39. Mayer, U., Ungerer, N., Klimmeck, D., Warnken, U., Schnolzer, M., Frings, S. and Mohrlen, F. (2008) Proteomic analysis of a membrane preparation from rat olfactory sensory cilia. *Chem Senses*, **33**, 145-162.
40. Siljee, J.E., Wang, Y., Bernard, A.A., Ersoy, B.A., Zhang, S., Marley, A., Von Zastrow, M., Reiter, J.F. and Vaisse, C. (2018) Subcellular localization of MC4R with ADCY3 at neuronal primary cilia underlies a common pathway for genetic predisposition to obesity. *Nat Genet*, **50**, 180-185.
41. Qian, M., Yao, S., Jing, L., He, J., Xiao, C., Zhang, T., Meng, W., Zhu, H., Xu, H. and Mo, X. (2013) ENC1-like integrates the retinoic acid/FGF signaling pathways to modulate ciliogenesis of Kupffer's Vesicle during zebrafish embryonic development. *Dev Biol*, **374**, 85-95.
42. Shiima-Kinoshita, C., Min, K.Y., Hanafusa, T., Mori, H. and Nakahari, T. (2004) Beta 2-adrenergic regulation of ciliary beat frequency in rat bronchiolar epithelium: potentiation by isosmotic cell shrinkage. *J Physiol*, **554**, 403-416.
43. Li, J.B., Gerdes, J.M., Haycraft, C.J., Fan, Y., Teslovich, T.M., May-Simera, H., Li, H., Blacque, O.E., Li, L., Leitch, C.C. *et al.* (2004) Comparative genomics identifies a flagellar

and basal body proteome that includes the BBS5 human disease gene. *Cell*, **117**, 541-552.

44. Schmidt, E.E., Pelz, O., Buhlmann, S., Kerr, G., Horn, T. and Boutros, M. (2013) GenomeRNAi: a database for cell-based and in vivo RNAi phenotypes, 2013 update. *Nucleic Acids Res*, **41**, D1021-1026.
45. Broekhuis, J.R., Verhey, K.J. and Jansen, G. (2014) Regulation of cilium length and intraflagellar transport by the RCK-kinases ICK and MOK in renal epithelial cells. *PLoS One*, **9**, e108470.
46. Tu, C.T., Yang, T.C., Huang, H.Y. and Tsai, H.J. (2012) Zebrafish arl6ip1 is required for neural crest development during embryogenesis. *PLoS One*, **7**, e32899.
47. Gao, J., DeRouen, M.C., Chen, C.H., Nguyen, M., Nguyen, N.T., Ido, H., Harada, K., Sekiguchi, K., Morgan, B.A., Miner, J.H. *et al.* (2008) Laminin-511 is an epithelial message promoting dermal papilla development and function during early hair morphogenesis. *Genes Dev*, **22**, 2111-2124.
48. Wang, W., Wu, T. and Kirschner, M.W. (2014) The master cell cycle regulator APC-Cdc20 regulates ciliary length and disassembly of the primary cilium. *Elife*, **3**, e03083.
49. Morse, D.M., Smullen, J.L. and Davis, C.W. (2001) Differential effects of UTP, ATP, and adenosine on ciliary activity of human nasal epithelial cells. *Am J Physiol Cell Physiol*, **280**, C1485-1497.
50. Choi, Y.H., Suzuki, A., Hajarnis, S., Ma, Z., Chapin, H.C., Caplan, M.J., Pontoglio, M., Somlo, S. and Igarashi, P. (2011) Polycystin-2 and phosphodiesterase 4C are components of a ciliary A-kinase anchoring protein complex that is disrupted in cystic kidney diseases. *Proc Natl Acad Sci U S A*, **108**, 10679-10684.
51. Spalluto, C., Wilson, D.I. and Hearn, T. (2013) Evidence for reciliation of RPE1 cells in late G1 phase, and ciliary localisation of cyclin B1. *FEBS Open Bio*, **3**, 334-340.
52. Filges, I., Nosova, E., Bruder, E., Tercanli, S., Townsend, K., Gibson, W.T., Rothlisberger, B., Heinimann, K., Hall, J.G., Gregory-Evans, C.Y. *et al.* (2014) Exome sequencing identifies mutations in KIF14 as a novel cause of an autosomal recessive lethal fetal ciliopathy phenotype. *Clin Genet*, **86**, 220-228.
53. Slaats, G.G., Wheway, G., Foletto, V., Szymanska, K., van Balkom, B.W., Logister, I., Den Ouden, K., Keijzer-Veen, M.G., Lilien, M.R., Knoers, N.V. *et al.* (2015) Screen-based identification and validation of four new ion channels as regulators of renal ciliogenesis. *J Cell Sci*, **128**, 4550-4559.
54. Kilburn, C.L., Pearson, C.G., Romijn, E.P., Meehl, J.B., Giddings, T.H., Jr., Culver, B.P., Yates, J.R., 3rd and Winey, M. (2007) New Tetrahymena basal body protein components identify basal body domain structure. *J Cell Biol*, **178**, 905-912.
55. Smiraldo, P.G., Gruver, A.M., Osborn, J.C. and Pittman, D.L. (2005) Extensive chromosomal instability in Rad51d-deficient mouse cells. *Cancer Res*, **65**, 2089-2096.
56. Saigusa, K., Imoto, I., Tanikawa, C., Aoyagi, M., Ohno, K., Nakamura, Y. and Inazawa, J. (2007) RGC32, a novel p53-inducible gene, is located on centrosomes during mitosis and results in G2/M arrest. *Oncogene*, **26**, 1110-1121.
57. Hoon, J.L., Li, H.Y. and Koh, C.G. (2014) POPX2 phosphatase regulates cell polarity and centrosome placement. *Cell Cycle*, **13**, 2459-2468.

58. Sakai, H., Urano, T., Ookata, K., Kim, M.H., Hirai, Y., Saito, M., Nojima, Y. and Ishikawa, F. (2002) MBD3 and HDAC1, two components of the NuRD complex, are localized at Aurora-A-positive centrosomes in M phase. *J Biol Chem*, **277**, 48714-48723.
59. Tanaka, N., Goto, M., Kawasaki, A., Sasano, T., Eto, K., Nishi, R., Sugasawa, K., Abe, S. and Saitoh, H. (2010) An EF-hands protein, centrin-1, is an EGTA-sensitive SUMO-interacting protein in mouse testis. *Cell Biochem Funct*, **28**, 604-612.
60. Huang, A.M., Montagna, C., Sharan, S., Ni, Y., Ried, T. and Sterneck, E. (2004) Loss of CCAAT/enhancer binding protein delta promotes chromosomal instability. *Oncogene*, **23**, 1549-1557.
61. Millecamps, S., Gentil, B.J., Gros-Louis, F., Rouleau, G. and Julien, J.P. (2005) Alsin is partially associated with centrosome in human cells. *Biochim Biophys Acta*, **1745**, 84-100.
62. Oeffner, F., Moch, C., Neundorf, A., Hofmann, J., Koch, M. and Grzeschik, K.H. (2008) Novel interaction partners of Bardet-Biedl syndrome proteins. *Cell Motil Cytoskeleton*, **65**, 143-155.
63. Andersen, J.S., Wilkinson, C.J., Mayor, T., Mortensen, P., Nigg, E.A. and Mann, M. (2003) Proteomic characterization of the human centrosome by protein correlation profiling. *Nature*, **426**, 570-574.
64. Wonsey, D.R. and Follettie, M.T. (2005) Loss of the forkhead transcription factor FoxM1 causes centrosome amplification and mitotic catastrophe. *Cancer Res*, **65**, 5181-5189.
65. Brede, G., Solheim, J., Troen, G. and Prydz, H. (2000) Characterization of PSKH1, a novel human protein serine kinase with centrosomal, golgi, and nuclear localization. *Genomics*, **70**, 82-92.
66. Menon, S., Oh, W., Carr, H.S. and Frost, J.A. (2013) Rho GTPase-independent regulation of mitotic progression by the RhoGEF Net1. *Mol Biol Cell*, **24**, 2655-2667.
67. Hussain, M.S., Baig, S.M., Neumann, S., Peche, V.S., Szczepanski, S., Nurnberg, G., Tariq, M., Jameel, M., Khan, T.N., Fatima, A. *et al.* (2013) CDK6 associates with the centrosome during mitosis and is mutated in a large Pakistani family with primary microcephaly. *Hum Mol Genet*, **22**, 5199-5214.
68. Pan, H.W., Chou, H.Y., Liu, S.H., Peng, S.Y., Liu, C.L. and Hsu, H.C. (2006) Role of L2DTL, cell cycle-regulated nuclear and centrosome protein, in aggressive hepatocellular carcinoma. *Cell Cycle*, **5**, 2676-2687.
69. Muller, H., Schmidt, D., Steinbrink, S., Mirgorodskaya, E., Lehmann, V., Habermann, K., Dreher, F., Gustavsson, N., Kessler, T., Lehrach, H. *et al.* (2010) Proteomic and functional analysis of the mitotic Drosophila centrosome. *EMBO J*, **29**, 3344-3357.
70. Chien, S.C., Brinkmann, E.M., Teuliere, J. and Garriga, G. (2013) Caenorhabditis elegans PIG-1/MELK acts in a conserved PAR-4/LKB1 polarity pathway to promote asymmetric neuroblast divisions. *Genetics*, **193**, 897-909.
71. Rhys, A.D., Monteiro, P., Smith, C., Vaghela, M., Arnandis, T., Kato, T., Leitinger, B., Sahai, E., McAinsh, A., Charras, G. *et al.* (2018) Loss of E-cadherin provides tolerance to centrosome amplification in epithelial cancer cells. *J Cell Biol*, **217**, 195-209.
72. Zhang, L., Iyer, J., Chowdhury, A., Ji, M., Xiao, L., Yang, S., Chen, Y., Tsai, M.Y. and Dong, J. (2012) KIBRA regulates aurora kinase activity and is required for precise chromosome alignment during mitosis. *J Biol Chem*, **287**, 34069-34077.

73. Zhu, C., Zhao, J., Bibikova, M., Leverson, J.D., Bossy-Wetzel, E., Fan, J.B., Abraham, R.T. and Jiang, W. (2005) Functional analysis of human microtubule-based motor proteins, the kinesins and dyneins, in mitosis/cytokinesis using RNA interference. *Mol Biol Cell*, **16**, 3187-3199.
74. Srinivas, V., Kitagawa, M., Wong, J., Liao, P.J. and Lee, S.H. (2015) The Tumor Suppressor Cdkn3 Is Required for Maintaining the Proper Number of Centrosomes by Regulating the Centrosomal Stability of Mps1. *Cell Rep*, **13**, 1569-1577.
75. Nam, H.J., Chae, S., Jang, S.H., Cho, H. and Lee, J.H. (2010) The PI3K-Akt mediates oncogenic Met-induced centrosome amplification and chromosome instability. *Carcinogenesis*, **31**, 1531-1540.
76. Faivre, J., Frank-Vaillant, M., Poulhe, R., Mouly, H., Jessus, C., Brechot, C. and Sobczak-Thepot, J. (2002) Centrosome overduplication, increased ploidy and transformation in cells expressing endoplasmic reticulum-associated cyclin A2. *Oncogene*, **21**, 1493-1500.
77. Nalepa, G., Enzor, R., Sun, Z., Marchal, C., Park, S.J., Yang, Y., Tedeschi, L., Kelich, S., Hanenberg, H. and Clapp, D.W. (2013) Fanconi anemia signaling network regulates the spindle assembly checkpoint. *J Clin Invest*, **123**, 3839-3847.
78. Mirzaa, G.M., Vitre, B., Carpenter, G., Abramowicz, I., Gleeson, J.G., Paciorkowski, A.R., Cleveland, D.W., Dobyns, W.B. and O'Driscoll, M. (2014) Mutations in CENPE define a novel kinetochore-centromeric mechanism for microcephalic primordial dwarfism. *Hum Genet*, **133**, 1023-1039.
79. Fielding, A.B., Lim, S., Montgomery, K., Dobreva, I. and Dedhar, S. (2011) A critical role of integrin-linked kinase, ch-TOG and TACC3 in centrosome clustering in cancer cells. *Oncogene*, **30**, 521-534.
80. Kinoshita, K., Noetzel, T.L., Pelletier, L., Mechtler, K., Drechsel, D.N., Schwager, A., Lee, M., Raff, J.W. and Hyman, A.A. (2005) Aurora A phosphorylation of TACC3/maskin is required for centrosome-dependent microtubule assembly in mitosis. *J Cell Biol*, **170**, 1047-1055.
81. Jakobsen, L., Vanselow, K., Skogs, M., Toyoda, Y., Lundberg, E., Poser, I., Falkenby, L.G., Bennetzen, M., Westendorf, J., Nigg, E.A. *et al.* (2011) Novel asymmetrically localizing components of human centrosomes identified by complementary proteomics methods. *EMBO J*, **30**, 1520-1535.
82. Genin, A., Desir, J., Lambert, N., Biervliet, M., Van Der Aa, N., Pierquin, G., Killian, A., Tosi, M., Urbina, M., Lefort, A. *et al.* (2012) Kinetochore KMN network gene CASC5 mutated in primary microcephaly. *Hum Mol Genet*, **21**, 5306-5317.
83. Theis, M., Slabicki, M., Junqueira, M., Paszkowski-Rogacz, M., Sontheimer, J., Kittler, R., Heninger, A.K., Glatter, T., Kruusmaa, K., Poser, I. *et al.* (2009) Comparative profiling identifies C13orf3 as a component of the Ska complex required for mammalian cell division. *EMBO J*, **28**, 1453-1465.
84. Kotian, S., Banerjee, T., Lockhart, A., Huang, K., Catalyurek, U.V. and Parvin, J.D. (2014) NUSAP1 influences the DNA damage response by controlling BRCA1 protein levels. *Cancer Biol Ther*, **15**, 533-543.
85. Bhat, V., Girimaji, S.C., Mohan, G., Arvinda, H.R., Singhmar, P., Duvvari, M.R. and Kumar, A. (2011) Mutations in WDR62, encoding a centrosomal and nuclear protein, in Indian primary microcephaly families with cortical malformations. *Clin Genet*, **80**, 532-540.

86. Xu, D., Zhang, F., Wang, Y., Sun, Y. and Xu, Z. (2014) Microcephaly-associated protein WDR62 regulates neurogenesis through JNK1 in the developing neocortex. *Cell Rep*, **6**, 104-116.
87. Nam, H.J. and van Deursen, J.M. (2014) Cyclin B2 and p53 control proper timing of centrosome separation. *Nat Cell Biol*, **16**, 538-549.
88. Hussain, M.S., Battaglia, A., Szczepanski, S., Kaygusuz, E., Toliat, M.R., Sakakibara, S., Altmuller, J., Thiele, H., Nurnberg, G., Moosa, S. *et al.* (2014) Mutations in CKAP2L, the human homolog of the mouse Radmis gene, cause Filippi syndrome. *Am J Hum Genet*, **95**, 622-632.
89. Huertas, D., Soler, M., Moreto, J., Villanueva, A., Martinez, A., Vidal, A., Charlton, M., Moffat, D., Patel, S., McDermott, J. *et al.* (2012) Antitumor activity of a small-molecule inhibitor of the histone kinase Haspin. *Oncogene*, **31**, 1408-1418.
90. Wang, X., Yang, Y., Duan, Q., Jiang, N., Huang, Y., Darzynkiewicz, Z. and Dai, W. (2008) sSgo1, a major splice variant of Sgo1, functions in centriole cohesion where it is regulated by Plk1. *Dev Cell*, **14**, 331-341.
91. Welburn, J.P., Grishchuk, E.L., Backer, C.B., Wilson-Kubalek, E.M., Yates, J.R., 3rd and Cheeseman, I.M. (2009) The human kinetochore Ska1 complex facilitates microtubule depolymerization-coupled motility. *Dev Cell*, **16**, 374-385.
92. Yang, S., Liu, X., Yin, Y., Fukuda, M.N. and Zhou, J. (2008) Tastin is required for bipolar spindle assembly and centrosome integrity during mitosis. *FASEB J*, **22**, 1960-1972.
93. Martinez-Garay, I., Rustom, A., Gerdes, H.H. and Kutsche, K. (2006) The novel centrosomal associated protein CEP55 is present in the spindle midzone and the midbody. *Genomics*, **87**, 243-253.
94. Zhong, X., Liu, L., Zhao, A., Pfeifer, G.P. and Xu, X. (2005) The abnormal spindle-like, microcephaly-associated (ASPM) gene encodes a centrosomal protein. *Cell Cycle*, **4**, 1227-1229.
95. Wu, J.M., Chen, C.T., Coumar, M.S., Lin, W.H., Chen, Z.J., Hsu, J.T., Peng, Y.H., Shiao, H.Y., Lin, W.H., Chu, C.Y. *et al.* (2013) Aurora kinase inhibitors reveal mechanisms of HURP in nucleation of centrosomal and kinetochore microtubules. *Proc Natl Acad Sci U S A*, **110**, E1779-1787.
96. Barthelmes, H.U., Grue, P., Feineis, S., Straub, T. and Boege, F. (2000) Active DNA topoisomerase IIalpha is a component of the salt-stable centrosome core. *J Biol Chem*, **275**, 38823-38830.
97. Hori, T., Haraguchi, T., Hiraoka, Y., Kimura, H. and Fukagawa, T. (2003) Dynamic behavior of Nuf2-Hec1 complex that localizes to the centrosome and centromere and is essential for mitotic progression in vertebrate cells. *J Cell Sci*, **116**, 3347-3362.
98. Bonnet, J., Coopman, P. and Morris, M.C. (2008) Characterization of centrosomal localization and dynamics of Cdc25C phosphatase in mitosis. *Cell Cycle*, **7**, 1991-1998.
99. Chen, D., Ito, S., Hyodo, T., Asano-Inami, E., Yuan, H. and Senga, T. (2017) Phosphorylation of DEPDC1 at Ser110 is required to maintain centrosome organization during mitosis. *Exp Cell Res*, **358**, 101-110.
100. Blachon, S., Gopalakrishnan, J., Omori, Y., Polyanovsky, A., Church, A., Nicastro, D., Malicki, J. and Avidor-Reiss, T. (2008) Drosophila asterless and vertebrate Cep152 Are orthologs essential for centriole duplication. *Genetics*, **180**, 2081-2094.

101. Cizmecioglu, O., Arnold, M., Bahtz, R., Settele, F., Ehret, L., Haselmann-Weiss, U., Antony, C. and Hoffmann, I. (2010) Cep152 acts as a scaffold for recruitment of Plk4 and CPAP to the centrosome. *J Cell Biol*, **191**, 731-739.
102. Huang, S., Ma, J., Liu, X., Zhang, Y. and Luo, L. (2011) Geminin is required for left-right patterning through regulating Kupffer's vesicle formation and ciliogenesis in zebrafish. *Biochem Biophys Res Commun*, **410**, 164-169.
103. Lu, F., Lan, R., Zhang, H., Jiang, Q. and Zhang, C. (2009) Geminin is partially localized to the centrosome and plays a role in proper centrosome duplication. *Biol Cell*, **101**, 273-285.
104. David, A., Liu, F., Tibelius, A., Vulprecht, J., Wald, D., Rothermel, U., Ohana, R., Seitel, A., Metzger, J., Ashery-Padan, R. *et al.* (2014) Lack of centrioles and primary cilia in STIL(-/-) mouse embryos. *Cell Cycle*, **13**, 2859-2868.
105. Vulprecht, J., David, A., Tibelius, A., Castiel, A., Konotop, G., Liu, F., Bestvater, F., Raab, M.S., Zentgraf, H., Izraeli, S. *et al.* (2012) STIL is required for centriole duplication in human cells. *J Cell Sci*, **125**, 1353-1362.
106. Bettencourt-Dias, M., Rodrigues-Martins, A., Carpenter, L., Riparbelli, M., Lehmann, L., Gatt, M.K., Carmo, N., Balloux, F., Callaini, G. and Glover, D.M. (2005) SAK/PLK4 is required for centriole duplication and flagella development. *Curr Biol*, **15**, 2199-2207.
107. Coelho, P.A., Bury, L., Shahbazi, M.N., Liakath-Ali, K., Tate, P.H., Wormald, S., Hindley, C.J., Huch, M., Archer, J., Skarnes, W.C. *et al.* (2015) Over-expression of Plk4 induces centrosome amplification, loss of primary cilia and associated tissue hyperplasia in the mouse. *Open Biol*, **5**, 150209.
108. Martin, C.A., Ahmad, I., Klingseisen, A., Hussain, M.S., Bicknell, L.S., Leitch, A., Nurnberg, G., Toliat, M.R., Murray, J.E., Hunt, D. *et al.* (2014) Mutations in PLK4, encoding a master regulator of centriole biogenesis, cause microcephaly, growth failure and retinopathy. *Nat Genet*, **46**, 1283-1292.
109. Koparir, A., Karatas, O.F., Yuceturk, B., Yuksel, B., Bayrak, A.O., Gerdan, O.F., Sagiroglu, M.S., Gezdirici, A., Kirimtay, K., Selcuk, E. *et al.* (2015) Novel POC1A mutation in primordial dwarfism reveals new insights for centriole biogenesis. *Hum Mol Genet*, **24**, 5378-5387.
110. Shaheen, R., Faqeih, E., Shamseldin, H.E., Noche, R.R., Sunker, A., Alshammari, M.J., Al-Sheddi, T., Adly, N., Al-Dosari, M.S., Megason, S.G. *et al.* (2012) POC1A truncation mutation causes a ciliopathy in humans characterized by primordial dwarfism. *Am J Hum Genet*, **91**, 330-336.
111. Izumi, H., Matsumoto, Y., Ikeuchi, T., Saya, H., Kajii, T. and Matsuura, S. (2009) BubR1 localizes to centrosomes and suppresses centrosome amplification via regulating Plk1 activity in interphase cells. *Oncogene*, **28**, 2806-2820.
112. Miyamoto, T., Porazinski, S., Wang, H., Borovina, A., Ciruna, B., Shimizu, A., Kajii, T., Kikuchi, A., Furutani-Seiki, M. and Matsuura, S. (2011) Insufficiency of BUBR1, a mitotic spindle checkpoint regulator, causes impaired ciliogenesis in vertebrates. *Hum Mol Genet*, **20**, 2058-2070.
113. Cheng, T.S., Hsiao, Y.L., Lin, C.C., Hsu, C.M., Chang, M.S., Lee, C.I., Yu, R.C., Huang, C.Y., Howng, S.L. and Hong, Y.R. (2007) hNinein is required for targeting spindle-associated

protein Astrin to the centrosome during the S and G2 phases. *Exp Cell Res*, **313**, 1710-1721.
114. Kersten, F.F., van Wijk, E., Hetterschijt, L., Baubeta, K., Peters, T.A., Aslanyan, M.G., van der Zwaag, B., Wolfrum, U., Keunen, J.E., Roepman, R. *et al.* (2012) The mitotic spindle protein SPAG5/Astrin connects to the Usher protein network postmitotically. *Cilia*, **1**, 2.
115. Seeger-Nukpezah, T., Liebau, M.C., Hopker, K., Lamkemeyer, T., Benzing, T., Golemis, E.A. and Schermer, B. (2012) The centrosomal kinase Plk1 localizes to the transition zone of primary cilia and induces phosphorylation of nephrocystin-1. *PLoS One*, **7**, e38838.
116. Shukla, A., Kong, D., Sharma, M., Magidson, V. and Loncarek, J. (2015) Plk1 relieves centriole block to reduplication by promoting daughter centriole maturation. *Nat Commun*, **6**, 8077.
117. Wang, G., Chen, Q., Zhang, X., Zhang, B., Zhuo, X., Liu, J., Jiang, Q. and Zhang, C. (2013) PCM1 recruits Plk1 to the pericentriolar matrix to promote primary cilia disassembly before mitotic entry. *J Cell Sci*, **126**, 1355-1365.
118. Wu, J., Mikule, K., Wang, W., Su, N., Petteruti, P., Gharahdaghi, F., Code, E., Zhu, X., Jacques, K., Lai, Z. *et al.* (2013) Discovery and mechanistic study of a small molecule inhibitor for motor protein KIFC1. *ACS Chem Biol*, **8**, 2201-2208.
119. Pugacheva, E.N., Jablonski, S.A., Hartman, T.R., Henske, E.P. and Golemis, E.A. (2007) HEF1-dependent Aurora A activation induces disassembly of the primary cilium. *Cell*, **129**, 1351-1363.
120. Zhou, H., Kuang, J., Zhong, L., Kuo, W.L., Gray, J.W., Sahin, A., Brinkley, B.R. and Sen, S. (1998) Tumour amplified kinase STK15/BTAK induces centrosome amplification, aneuploidy and transformation. *Nat Genet*, **20**, 189-193.
121. Waters, A.M., Asfahani, R., Carroll, P., Bicknell, L., Lescai, F., Bright, A., Chanudet, E., Brooks, A., Christou-Savina, S., Osman, G. *et al.* (2015) The kinetochore protein, CENPF, is mutated in human ciliopathy and microcephaly phenotypes. *J Med Genet*, **52**, 147-156.
122. Bondeson, M.L., Ericson, K., Gudmundsson, S., Ameur, A., Ponten, F., Wesstrom, J., Frykholm, C. and Wilbe, M. (2017) A nonsense mutation in CEP55 defines a new locus for a Meckel-like syndrome, an autosomal recessive lethal fetal ciliopathy. *Clin Genet*, **92**, 510-516.
123. Dou, Z., Ding, X., Zereshki, A., Zhang, Y., Zhang, J., Wang, F., Sun, J., Huang, H. and Yao, X. (2004) TTK kinase is essential for the centrosomal localization of TACC2. *FEBS Lett*, **572**, 51-56.
124. Majumder, S. and Fisk, H.A. (2013) VDAC3 and Mps1 negatively regulate ciliogenesis. *Cell Cycle*, **12**, 849-858.
125. Huang, T.W., Cheng, P.W., Chan, Y.H., Yeh, T.H., Young, Y.H. and Young, T.H. (2010) Regulation of ciliary differentiation of human respiratory epithelial cells by the receptor for hyaluronan-mediated motility on hyaluronan-based biomaterials. *Biomaterials*, **31**, 6701-6709.
126. Maxwell, C.A., Keats, J.J., Crainie, M., Sun, X., Yen, T., Shibuya, E., Hendzel, M., Chan, G. and Pilarski, L.M. (2003) RHAMM is a centrosomal protein that interacts with dynein and maintains spindle pole stability. *Mol Biol Cell*, **14**, 2262-2276.

127. Fry, A.M., Meraldi, P. and Nigg, E.A. (1998) A centrosomal function for the human Nek2 protein kinase, a member of the NIMA family of cell cycle regulators. *EMBO J*, **17**, 470-481.
128. Spalluto, C., Wilson, D.I. and Hearn, T. (2012) Nek2 localises to the distal portion of the mother centriole/basal body and is required for timely cilium disassembly at the G2/M transition. *Eur J Cell Biol*, **91**, 675-686.
129. May-Simera, H.L., Gumerson, J.D., Gao, C., Campos, M., Cologna, S.M., Beyer, T., Boldt, K., Kaya, K.D., Patel, N., Kretschmer, F. *et al.* (2016) Loss of MACF1 Abolishes Ciliogenesis and Disrupts Apicobasal Polarity Establishment in the Retina. *Cell Rep*, **17**, 1399-1413.
130. Stelzer, G., Rosen, N., Plaschkes, I., Zimmerman, S., Twik, M., Fishilevich, S., Stein, T.I., Nudel, R., Lieder, I., Mazor, Y. *et al.* (2016) The GeneCards Suite: From Gene Data Mining to Disease Genome Sequence Analyses. *Curr Protoc Bioinformatics*, **54**, 1 30 31-31 30 33.
131. Spektor, A., Tsang, W.Y., Khoo, D. and Dynlacht, B.D. (2007) Cep97 and CP110 suppress a cilia assembly program. *Cell*, **130**, 678-690.
132. Tang, Z., Lin, M.G., Stowe, T.R., Chen, S., Zhu, M., Stearns, T., Franco, B. and Zhong, Q. (2013) Autophagy promotes primary ciliogenesis by removing OFD1 from centriolar satellites. *Nature*, **502**, 254-257.
133. Gomperts, B.N., Gong-Cooper, X. and Hackett, B.P. (2004) Foxj1 regulates basal body anchoring to the cytoskeleton of ciliated pulmonary epithelial cells. *J Cell Sci*, **117**, 1329-1337.
134. Paoloni-Giacobino, A., Kern, I., Rumpler, Y., Djlelati, R., Morris, M.A. and Dahoun, S.P. (2000) Familial t(6;21)(p21.1;p13) translocation associated with male-only sterility. *Clin Genet*, **58**, 324-328.
135. Potter, C., Zhu, W., Razafsky, D., Ruzycki, P., Kolesnikov, A.V., Doggett, T., Kefalov, V.J., Betleja, E., Mahjoub, M.R. and Hodzic, D. (2017) Multiple Isoforms of Nesprin1 Are Integral Components of Ciliary Rootlets. *Curr Biol*, **27**, 2014-2022 e2016.
136. Firat-Karalar, E.N., Sante, J., Elliott, S. and Stearns, T. (2014) Proteomic analysis of mammalian sperm cells identifies new components of the centrosome. *J Cell Sci*, **127**, 4128-4133.
137. Chiyotani, A., Tamaoki, J., Sakai, N., Isono, K., Kondo, M. and Konno, K. (1992) Thromboxane A2 mimetic U46619 stimulates ciliary motility of rabbit tracheal epithelial cells. *Prostaglandins*, **43**, 111-120.
138. Bhogaraju, S., Cajanek, L., Fort, C., Blisnick, T., Weber, K., Taschner, M., Mizuno, N., Lamla, S., Bastin, P., Nigg, E.A. *et al.* (2013) Molecular basis of tubulin transport within the cilium by IFT74 and IFT81. *Science*, **341**, 1009-1012.
139. Faisst, A.M., Alvarez-Bolado, G., Treichel, D. and Gruss, P. (2002) Rotatin is a novel gene required for axial rotation and left-right specification in mouse embryos. *Mech Dev*, **113**, 15-28.
140. Kheradmand Kia, S., Verbeek, E., Engelen, E., Schot, R., Poot, R.A., de Coo, I.F., Lequin, M.H., Poulton, C.J., Pourfarzad, F., Grosveld, F.G. *et al.* (2012) RTTN mutations link primary cilia function to organization of the human cerebral cortex. *Am J Hum Genet*, **91**, 533-540.
141. Shamseldin, H., Alazami, A.M., Manning, M., Hashem, A., Caluseiu, O., Tabarki, B., Esplin, E., Schelley, S., Innes, A.M., Parboosingh, J.S. *et al.* (2015) RTTN Mutations Cause

Primary Microcephaly and Primordial Dwarfism in Humans. *Am J Hum Genet*, **97**, 862-868.
142. Delmaghani, S., Aghaie, A., Michalski, N., Bonnet, C., Weil, D. and Petit, C. (2012) Defect in the gene encoding the EAR/EPTP domain-containing protein TSPEAR causes DFNB98 profound deafness. *Hum Mol Genet*, **21**, 3835-3844.
143. Yu, J., Carroll, T.J., Rajagopal, J., Kobayashi, A., Ren, Q. and McMahon, A.P. (2009) A Wnt7b-dependent pathway regulates the orientation of epithelial cell division and establishes the cortico-medullary axis of the mammalian kidney. *Development*, **136**, 161-171.
144. Arnaiz, O., Gout, J.F., Betermier, M., Bouhouche, K., Cohen, J., Duret, L., Kapusta, A., Meyer, E. and Sperling, L. (2010) Gene expression in a paleopolyploid: a transcriptome resource for the ciliate Paramecium tetraurelia. *BMC Genomics*, **11**, 547.
145. Avidor-Reiss, T., Maer, A.M., Koundakjian, E., Polyanovsky, A., Keil, T., Subramaniam, S. and Zuker, C.S. (2004) Decoding cilia function: defining specialized genes required for compartmentalized cilia biogenesis. *Cell*, **117**, 527-539.
146. Kong, L., Yin, H. and Yuan, L. (2017) Centrosomal MCM7 strengthens the Cep68-VHL interaction and excessive MCM7 leads to centrosome splitting resulting from increase in Cep68 ubiquitination and proteasomal degradation. *Biochem Biophys Res Commun*, **489**, 497-502.
147. Ferguson, R.L., Pascreau, G. and Maller, J.L. (2010) The cyclin A centrosomal localization sequence recruits MCM5 and Orc1 to regulate centrosome reduplication. *J Cell Sci*, **123**, 2743-2749.
148. Gupta, A., Tsuchiya, Y., Ohta, M., Shiratsuchi, G. and Kitagawa, D. (2017) NEK7 is required for G1 progression and procentriole formation. *Mol Biol Cell*, **28**, 2123-2134.

ITEM TO CHECK	IMPORTANCE	CHECKLIST	COMMENTS
**EXPERIMENTAL DESIGN**			
Definition of experimental and control groups	E	✔	given in Figures and Figure legends
Number within each group	E	✔	given in Figures and Figure legends
Assay carried out by core lab or investigator's lab?	D	✔	Investigator's lab
Acknowledgement of authors' contributions	D	✔	qPCRs were performed by TCT, CD, MDB and MP
**SAMPLE**			
Description	E	✔	hTert immortalized 1BR3 fibroblasts or zebrafish embryos
Volume/mass of sample processed	D	✔	150,000-180,000 cells or at least 20 embryos per RNA extraction
Microdissection or macrodissection	E	✔	Embryos were microdissected out of their chorion
Processing procedure	E	✔	Not applicable
If frozen - how and how quickly?	E	✔	Not applicable
If fixed - with what, how quickly?	E	✔	Not applicable
Sample storage conditions and duration (especially for FFPE samples)	E	✔	Not applicable
**NUCLEIC ACID EXTRACTION**			
Procedure and/or instrumentation	E	✔	RNA was extracted manually
Name of kit and details of any modifications	E	✔	Qiagen RNeasy Mini or Zymo Research Quick-RNA Miniprep Kit
Source of additional reagents used	D	✔	Applichem (β-Mercaptoethanol for Qiagen Kit), Ethanol (Merck)
Details of DNase or RNAse treatment	E	✔	DNaseI treatment included in Zymo Kit, Qiagen Kit: According to Qiagen's instructions with RNase-free DNAse set
Contamination assessment (DNA or RNA)	E		Not performed
Nucleic acid quantification	E	✔	by photometry
Instrument and method	E	✔	Nanodrop (Thermo Scientific), Absorption at 260 nm
Purity (A260/A280)	D	✔	at least 1.8
Yield	D	✔	Minimum yield obtained was 600 ng/sample
RNA integrity method/instrument	E	✔	Not applicable
RIN/RQI or Cq of 3' and 5' transcripts	E	✔	Not applicable
Electrophoresis traces	D	✔	Not applicable
Inhibition testing (Cq dilutions, spike or other)	E	✔	Not applicable
**REVERSE TRANSCRIPTION**			
Complete reaction conditions	E	✔	Equal amounts of total RNA were reversely transcribed in the presence of RNase inhibitors using the manufacturer's instructions
Amount of RNA and reaction volume	E	✔	at least 250 ng/ 20 µl reaction,
Priming oligonucleotide (if using GSP) and concentration	E	✔	oligodTTP
Reverse transcriptase and concentration	E	✔	SuperScript II or III: 200 U/µl, 1 µl/20 µl reaction
Temperature and time	E	✔	Reverse transcription for 1 hour at 42 ºC (SuperScriptII) or 50 ºC (SuperScript III)
Manufacturer of reagents and catalogue numbers	D	✔	RNAsin Plus (Promega, #N261B), SuperScript II RT (Life technologies, #18064-014), SuperScript III RT (Life technologies, #18080-044)
Cqs with and without RT	D*	✔	Not applicable
Storage conditions of cDNA	D	✔	minus 20 ºC if not used up right away
**qPCR TARGET INFORMATION**			
If multiplex, efficiency and LOD of each assay.	E	✔	Not applicable
Sequence accession number	E		AURKA: NM_198433.1, b2m: BC062841.1, CAST: BC013579.1 , CDC25C: NM_001790.3, CEP97: NM_024548.3, CEP152: NM_001194998.1, GLI1: NM_005269.2, gli1: NM_178296.2, IFT74: NM_025103.2, KLRG1: NM_005810.3, MACF1: NM_012090.5, MCM2: NM_004526.2, MCM7: NM_005916.4, MOK: NM_014226.1, NEK2: NM_002497.3, nkx2.2a: NM_131422.2, PDE4C: NM_000923.5, PTGDS: NM_000954.5, PLK1: NM_005030.3, PLK4: NM_014264.4, POC1A: NM_015426.4, PRC1: NM_003981.2, PTGS2: NM_000963.3, RTTN: NM_173630.3, SDHA: ENST00000264932.6, SLC31A2: NM_001860.2, STIL: NM_001048166.1, SYNE1: NM_182961.3, TBXAS1: NM_001061.4, UBE2C: NM_007019.2
Location of amplicon	D	✔	available upon request
Amplicon length	E	✔	betwee 60 and 200 nt
In silico specificity screen (BLAST, etc)	E		Not performed
Pseudogenes, retropseudogenes or other homologs?	D		Not performed
Sequence alignment	D		Not performed
Secondary structure analysis of amplicon	D		Not performed
Location of each primer by exon or intron (if applicable)	E	✔	Primers were designed to span introns, whenever possible
What splice variants are targeted?	E	✔	Normally the most abundant variant with the longest ORF
**qPCR OLIGONUCLEOTIDES**			
Primer sequences	E	✔	as provided in Table S1
RTPrimerDB Identification Number	D	✔	Not applicable
Probe sequences	D**	✔	not available from manufacturer, but numbers are given in Table S1
Location and identity of any modifications	E	✔	Not modified
Manufacturer of oligonucleotides	D	✔	IDT
Purification method	D	✔	Desalting
**qPCR PROTOCOL**			
Complete reaction conditions	E	✔	qPCRs were performed in white 96-well plates in a LightCycler 480. Each 12 µl reaction consisted of 1-4 µl cDNA, 1 µl of a 10 µM primer mix (consisting of forward and reverse primer), 0.125 µl Universal Probe, 6 µl 2x qPCR mix (containing dNTPs, Magnesium and polymerase) and ultrapure water. Primers were designed with the Roche Assay Design Center.
Reaction volume and amount of cDNA/DNA	E	✔	between 1 and 4 µl cDNA/12 µl reaction
Primer, (probe), Mg++ and dNTP concentrations	E	✔	Primers: 0.83 µM each, Universal Probes: 0.125 µl µl/12 µl reaction, Mg++ and dNTP provided in qPCR kit
Polymerase identity and concentration	E	✔	Manufacturer's proprietary
Buffer/kit identity and manufacturer	E	✔	Absolute QPCR ROX Master Mix (Thermo Fisher) or Luna Universal probe qPCR Master Mix (NEB) with Roche Universal Probe System
Exact chemical constitution of the buffer	D	✔	Manufacturer's proprietary
Additives (SYBR Green I, DMSO, etc.)	E	✔	Universal Probes (Roche)
Manufacturer of plates/tubes and catalog number	D	✔	Biozym, catalog no. 712282

Complete thermocycling parameters	**E**	✔	Absolute QPCR ROX Master Mix: 2 min 50ºC, 15 min 95 ºC, 40-45 cycles with 95 ºC for 10 sec and 60 ºC for 1 min; Luna Universal probe qPCR Master Mix: 1 min at 95 ºC; 40-45 cycles at 95 ºC for 15 sec and 60ºC for 30 sec
Reaction setup (manual/robotic)	D	✔	manual
Manufacturer of qPCR instrument	**E**	✔	Roche
**qPCR VALIDATION**			
Evidence of optimisation (from gradients)	D	✔	None
Specificity (gel, sequence, melt, or digest)	**E**	✔	Specifity given by assay design and Universal Probe
For SYBR Green I, Cq of the NTC	**E**	✔	Not applicable
Standard curves with slope and y-intercept	**E**		Not performed
PCR efficiency calculated from slope	**E**		Not performed
Confidence interval for PCR efficiency or standard error	D		Not performed
r2 of standard curve	**E**		Not performed
Linear dynamic range	**E**		Cq values were within linear range
Cq variation at lower limit	**E**		Not performed
Confidence intervals throughout range	D		Not performed
Evidence for limit of detection	**E**		Not performed
If multiplex, efficiency and LOD of each assay.	**E**	✔	Not applicable
**DATA ANALYSIS**			
qPCR analysis program (source, version)	**E**		LightCycler480 Software version 1.5.0 SP3
Cq method determination	**E**		Abs Quant/Second Derivative Maximum
Outlier identification and disposition	**E**		Not performed
Results of NTCs	**E**	✔	> 45 cycles
Justification of number and choice of reference genes	**E**	✔	Reference genes were chosen to come up after 20 cycles and based on PMID 19014500
Description of normalisation method	**E**	✔	All data normalized to housekeeping gene and control conditions
Number and concordance of biological replicates	D	✔	at least 3 biological replicates
Number and stage (RT or qPCR) of technical replicates	**E**	✔	qPCR reaction in triplicates of each biological replicate
Repeatability (intra-assay variation)	E		Not determined
Reproducibility (inter-assay variation, %CV)	D		Not determined
Power analysis	D	✔	Not applicable
Statistical methods for result significance	**E**	✔	as given in the Figure Legends
Software (source, version)	E	✔	GraphPad Prism7
Cq or raw data submission using RDML	**D**	✔	Not applicable

Table 1. MIQE checklist for authors, reviewers and editors. All essential information (E) must be submitted with the manuscript. Desirable information (D) should be submitted if available. If using primers obtained from RTPrimerDB, information on qPCR target, oligonucleotides, protocols and validation is available from that source.

*: Assessing the absence of DNA using a no RT assay is essential when first extracting RNA. Once the sample has been validated as RDNA-free, inclusion of a no-RT control is desirable, but no longer essential.

**: Disclosure of the probe sequence is highly desirable and strongly encouraged. However, since not all commercial pre-designed assay vendors provide this information, it cannot be an essential requirement. Use of such assays is advised against.
